# Supplementary material for: STAndardised DIagnostic Assessment for children and young people with emotional difficulties (STADIA): protocol for a multicentre randomised controlled trial
Source: BMJ Open. 2022 May 10;12(5):e053043. doi: 10.1136/bmjopen-2021-053043 (PMC9096530; doi:10.1136/bmjopen-2021-053043)
Supplement: Supplementary data [file bmjopen-2021-053043supp001.pdf]

STAndardised DIagnostic Assessment for children and adolescents with emotional difficulties (STADIA): a multi-centre randomised controlled trial

SCREENING

**Site Number:**	☐☐
**Screening Number:**	☐☐☐
**Sponsor:**	**Nottinghamshire Healthcare NHS Foundation Trust**
**CRF Version:**	**Final v1.1 – 30 April 2019**

Site Number: ☐☐

Screening Number: ☐☐☐

REFERRAL SCREENING		
Complete for all referrals screened for eligibility:		
NHS Number<br>*Local use only*	____________________	
Trust Number<br>*Local use only*	____________________	
Date of referral receipt<br>(dd-mmm-yyyy)	☐☐ - ☐☐☐ - ☐☐☐☐	
Date of screening<br>(dd-mmm-yyyy)	☐☐ - ☐☐☐ - ☐☐☐☐	
Young person's sex		Male ☐<br>Female ☐
Young person's age<br>*If <5 or >17 do not proceed*		☐☐
Has the young person been previously enrolled and randomised in the STADIA trial?<br>*If yes, do not proceed*		Yes ☐<br>No ☐
Does the referral mention any of the following Covid-19 related words/phrases?<br>*Tick all that apply.*<br>Covid-19 / Covid<br>Coronavirus<br>Lockdown<br>School closure / exams cancelled		 <br>☐<br>☐<br>☐<br>☐
Does the referral mention emotional difficulties*?<br>*If no, do not proceed*		Yes ☐<br>No ☐
Is this an emergency or urgent referral (according to local CAMHS triage / SPA team risk assessment)?<br>*If yes, do not proceed*		Yes ☐<br>No ☐
Does the young person have severe learning disability (e.g., the referral mentions this or that they attend a special school for children with severe learning difficulties)?<br>*If 'yes' do not proceed*<br>*If not known, confirm during telephone eligibility check at enrolment*		Yes ☐<br>No ☐<br>Not known ☐

Day F, *et al. BMJ Open* 2022; 12:e053043. doi: 10.1136/bmjopen-2021-053043

Site Number:

Screening Number:

REFERRAL SCREENING	
If the young person is <16:<br>Does the referral information include contact details for a named parent/carer?<br>*If 'no' await parent/carer contact details before proceeding*	Yes ☐<br>No ☐<br>N/A ☐
If the young person is <16:<br>Is the named parent/carer a local authority representative designated to care for the child/young person?<br>*If 'yes' do not proceed*<br>*If not known, confirm during telephone eligibility check at enrolment*	Yes ☐<br>No ☐<br>Not known ☐<br>N/A ☐
If the young person is aged 16 or 17:<br>Whose contact details are given on the referral form?<br>*If young person contact details are provided, they should be contacted in the first instance*	Young person ☐<br>Parent/carer ☐<br>Both ☐<br>N/A ☐

EMOTIONAL DIFFICULTIES	
**Emotional difficulties may be indicated by the use of any of the following key words or phrases.*<br>*Tick all that apply. If 'other' record details and seek advice from the PI or NCTU before proceeding.*	
None	☐
Agitated / agitation	☐
Anger	☐
Anxiety / anxious / generalised anxiety	☐
Avoids things/people/places	☐
Can't leave the house	☐
Completing rituals / asking parents to carry out rituals	☐
Compulsions	☐
Depressed / depression / low / low mood / sad	☐
Difficulties sleeping	☐
Distress	☐
Fears and worries / fears relating to safety (germs, fire)	☐
Feeling low	☐
Feels flat / empty / blank	☐

Day F, *et al. BMJ Open* 2022; 12:e053043. doi: 10.1136/bmjopen-2021-053043

Site Number: | | |

Screening Number: | | | |

EMOTIONAL DIFFICULTIES	
**Emotional difficulties may be indicated by the use of any of the following key words or phrases. Tick all that apply. If 'other' record details and seek advice from the PI or NCTU before proceeding.*	
Feels hopeless	☐
Feels worthless / stupid	☐
Flashbacks	☐
Hypervigilance	☐
Irritable	☐
Low motivation	☐
Low self-esteem / Hates self	☐
Mood swings / moody	☐
Negative thoughts	☐
Nightmares (if trauma also present)	☐
No (or loss of) energy	☐
No (or loss of) interest in things / gave up… / lack of wanting to do things	☐
Not going to school / unable to go to school	☐
Not sleeping / poor sleep	☐
Obsessions	☐
OCD	☐
Phobia	☐
Panic / panic attacks	☐
PTSD	☐
Self-harm / DSH / Cutting	☐
Suicidal	☐
Suicidal thoughts / thoughts of ending life / thinks about killing self	☐
Tearful	☐
Thoughts of death	☐

Day F, *et al. BMJ Open* 2022; 12:e053043. doi: 10.1136/bmjopen-2021-053043

Site Number: | | |

Screening Number: | | | |

EMOTIONAL DIFFICULTIES	
**Emotional difficulties may be indicated by the use of any of the following key words or phrases. Tick all that apply. If 'other' record details and seek advice from the PI or NCTU before proceeding.*	
Tiredness / fatigue	☐
Touching objects	☐
Trauma	☐
Weepy	☐
Withdrawal / withdrawn	☐
Worried / worrying (incl. worries/concerns about their appearance	☐
Other (please specify)	☐

FOR ALL REFERRALS SCREENED, ENTER SUMMARY DATA ON THE SCREENING & ENROLMENT LOG.

IF THE YOUNG PERSON APPEARS TO BE ELIGIBLE PROCEED TO THE INVITATION TELEPHONE CALL (CALL 1) AND ENTER DETAILS ON THE TRIAL DATABASE.

SIGN-OFF STATEMENT	
Completed by the researcher conducting the referral screening.	
To the best of my knowledge, I confirm that I have made every reasonable effort to ensure that ALL of the data in this Case Record Form is a true, accurate and complete report.	
Print Name	
Signature	
Date	_ _ - _ _ _ - _ _ _ _

Day F, *et al. BMJ Open* 2022; 12:e053043. doi: 10.1136/bmjopen-2021-053043
