# Supplementary material for: STAndardised DIagnostic Assessment for children and young people with emotional difficulties (STADIA): protocol for a multicentre randomised controlled trial
Source: BMJ Open. 2022 May 10;12(5):e053043. doi: 10.1136/bmjopen-2021-053043 (PMC9096530; doi:10.1136/bmjopen-2021-053043)
Supplement: Supplementary data [file bmjopen-2021-053043supp002.pdf]

ADD LOCAL HEADER

STandardised DIagnostic Assessment for children and young people with emotional difficulties (STADIA)

Informed Consent Form for the Parent/Carer

Final v2.0 13 August 2020

Name of Principal Investigator: [add local PI name]

IRAS Project ID: 255635

Participant Trial ID: | | | | | |
(To be completed after randomisation)

We are doing this research to find out how to make sure children and young people get the help they need when they are referred to CAMHS. We have invited you to take part in this research because a young person you care for has been referred to CAMHS. You can decide whether or not to take part in this research.
If you agree to take part in the STADIA Trial, please read and acknowledge each of the following statements.

A drop-down menu will be provided within the online electronic Informed Consent Form so that the person providing consent has the option to acknowledge/agree to each of the following statements.	
**1.**	I confirm that I have read and understand the Participant Information Sheet, Version <insert current PIS version number and date > for the above research. (*Only for the parent/carer of children/young people aged 11-15*) [My child and] I have had the opportunity to consider the information, ask questions and have had these answered satisfactorily.
**2.**	*Only for the parent/carer of children/young people aged 11-15*<br>I have spoken to my child about the research and they are aware of the study.
**3.**	I understand that mine and my child's participation is voluntary and that I am free to withdraw at any time, without giving any reason, and without my child's medical care or legal rights being affected. I understand that should I withdraw, then the information collected so far cannot be deleted and that this information may still be used in the research.
**4.**	I understand that relevant sections of my child's CAMHS records and data collected in the trial may be looked at by authorised individuals from the Nottingham Clinical Trials Unit (University of Nottingham), the Sponsor (Nottinghamshire Healthcare NHS Foundation Trust), NHS bodies, the trial research group and regulatory authorities where it is relevant to taking part in this study. I give permission for these individuals to have access to these records and for my consent form to be retained by the Nottingham Clinical Trials Unit.
**5.**	I give permission for the Nottingham Clinical Trials Unit, the Sponsor and the trial research group to collect, store, analyse and publish information obtained from mine and my child's participation in this trial. I understand that our personal details will be kept confidential.
**6.**	I understand that the Nottingham Clinical Trials Unit and the trial research group will be provided with mine and my child's personal details to send questionnaires by email and study-related correspondence during the trial. I give my permission for this information to be kept and for these individuals to contact me.
**7.**	I understand that if I fill out the DAWBA, I will receive a copy of the DAWBA report and a copy will also be provided to the CAMHS team and kept in my child's CAMHS records.
**8.**	I agree to my child's GP being informed of their participation in this trial.
**9.**	I understand that the anonymised information collected about me and my child may be used to support other research in the future and may be shared with other researchers.
**10.**	I agree to take part in the above trial.

Day F, *et al. BMJ Open* 2022; 12:e053043. doi: 10.1136/bmjopen-2021-053043

Please also answer yes or no to the following options.

A drop-down menu will be provided within the online electronic Informed Consent Form so that the person providing consent has the option to answer yes or no to each of the following optional statements.			
**1.**	*Interviews about your experiences*<br>I agree to be contacted about the STADIA interview study. I understand that there is no obligation to take part and I will just be informed of what the study will involve.	Yes	No
**2.**	*Future studies*<br>I agree to be contacted about other research studies in the future. I understand that there is no obligation to take part and I will just be informed of what the future research would involve.	Yes	No
**3.**	*Results of the STADIA study*<br>I would like to receive a summary of the results at the end of the STADIA study.	Yes	No
**4.**	*Only for the parent/carer of children/young people aged 11-15*<br>*Questionnaires*<br>I agree to my child being invited to complete questionnaires about their mood and feelings for the research.	Yes	No
**5.**	I consent to [INSERT NHS TRUST NAME] passing identifiable data (my child's NHS number, name and date of birth) to the organisations that are responsible for health information including NHS Digital. This will be used to request data from the Children and Young People's Health Services Data Set and the Mental Health Services Data Set.	Yes	No

Type your name here:

_________________________ _______________

Name of parent/carer Date [system generated]

Type the name of your child here:

_________________________ _______________

Name of child/young person Date [system generated]

System use only:

_________________________ _______________

Name of person taking consent Date [system generated]

(You must be on the delegation log)

NB. Signatures will not be collected as consent will be obtained online. Participants will be asked to complete the eICF and write their name before submitting the online form; the date will be system-generated. The name of the researcher who provided the study information and the date the eICF was generated will also be recorded within the online system.

The online electronic Informed Consent Form (eICF) will be retained within the trial database. Printable (PDF) copies will be generated and retained within the Investigator Site File and CAMHS records. A copy will be sent by email to the participant.

Day F, *et al. BMJ Open* 2022; 12:e053043. doi: 10.1136/bmjopen-2021-053043
