# Supplementary material for: STAndardised DIagnostic Assessment for children and young people with emotional difficulties (STADIA): protocol for a multicentre randomised controlled trial
Source: BMJ Open. 2022 May 10;12(5):e053043. doi: 10.1136/bmjopen-2021-053043 (PMC9096530; doi:10.1136/bmjopen-2021-053043)
Supplement: Supplementary data [file bmjopen-2021-053043supp003.pdf]

ADD LOCAL HEADER

STandardised DIagnostic Assessment for children and young people with emotional difficulties (STADIA)

Assent form for young people aged 11-15

Final v1.0 28-Mar-2019

Name of Principal Investigator: [add local PI name]

IRAS Project ID: 255635

Participant Trial ID: | | | | | |

(To be completed after randomisation)

We are doing this research to find out how to make sure people get the help they need when they are referred to CAMHS. We are asking you to help with this research but you can decide whether or not to take part.
If you agree to help with the STADIA Trial please answer the following questions.

A drop-down menu will be provided within the online electronic Assent Form so that the young person providing assent has the option to acknowledge/agree to each of the following statements.			
**1.**	Have you read the information about the research or has someone explained it to you?	Yes	No
**2.**	Do you understand what the research is about?	Yes	No
**3.**	Have you been able to ask all the questions you want?	Yes	No
**4.**	Do you understand that it's your choice whether or not to take part and it's OK to stop taking part at any time?	Yes	No
**5.**	Do you want to help with the research by completing some questionnaires about your mood and feelings?	Yes	No

Type your name here:

___________________________ ________________

Name of child/young person Date [system generated]

System use only:

___________________________ ________________

Name of person taking consent Date [system generated]
(You must be on the delegation log)

NB. Signatures will not be collected as consent will be obtained online. Participants will be asked to complete the eICF and write their name before submitting the online form; the date will be system-generated. The name of the researcher who provided the study information and the date the eICF was generated will also be recorded within the online system. The online electronic Informed Consent Form (eICF) will be retained within the trial database. Printable (PDF) copies will be generated and retained within the Investigator Site File and CAMHS records. A copy will be sent by email to the participant.

Day F, *et al. BMJ Open* 2022; 12:e053043. doi: 10.1136/bmjopen-2021-053043
