# Supplementary material for: STAndardised DIagnostic Assessment for children and young people with emotional difficulties (STADIA): protocol for a multicentre randomised controlled trial
Source: BMJ Open. 2022 May 10;12(5):e053043. doi: 10.1136/bmjopen-2021-053043 (PMC9096530; doi:10.1136/bmjopen-2021-053043)
Supplement: Supplementary data [file bmjopen-2021-053043supp004.pdf]

DAWBA Report

The DAWBA collects information about a range of common emotional and behavioural difficulties, and uses this information to produce a report to highlight the level of difficulties.

How to understand the ratings

These ratings compare your responses with the responses from large numbers of other parents and young people across the UK. Many parents and young people find this sort of comparison helpful, but it is just a guide and not the same as a face-to-face assessment with a specialist.

To make it easier to read, we have grouped the ratings into four categories. Each category is different. This shows how your [child's] *(delete as appropriate)* difficulties compare with other children / young people:

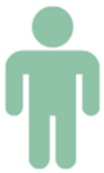

Close to average

In the general population most children/ young people (roughly 80 out of 100) are in the "close to average" category.

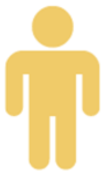

Slightly raised

If the ratings are in the "slightly raised" category this means the difficulties are slightly higher than average. Roughly 10 out of 100 children / young people are in this category.

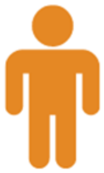

High

Around 5 in 100 children / young people score in the "high" category. This means that the difficulties are more severe than average.

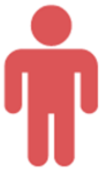

Very high

Around 5 in 100 children score in the "very high" category. This means that the difficulties appear to be more severe than we find in 95 out of every 100 children / young people.

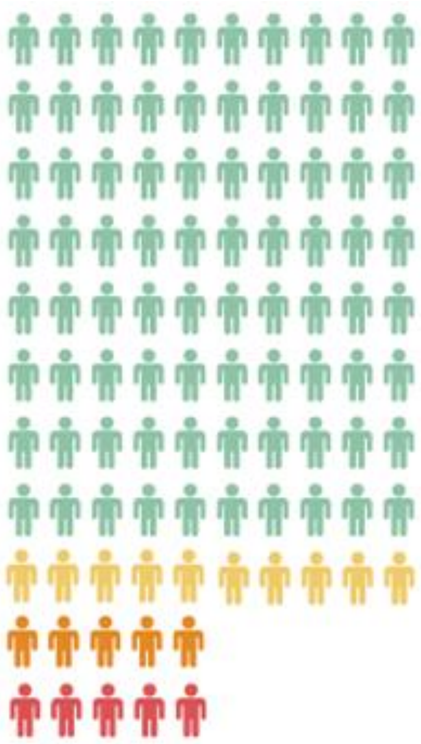

The rating is only a rough guide. As high ratings can be a "false alarm", please use your own judgement. Not all difficulties need treating. Some difficulties get better by themselves, particularly if they are mild or if they have only been there for a short time.

Most strengths and difficulties lie on a scale. There will be children / young people at each end of the scale but most children / young people will fall somewhere in between.

DAWBA report template Final Version 1.1 14 Jun 2019

Your [child's] *(delete as appropriate)* **ratings:**

- **Close to average / Slightly raised / High / Very high** for worrying a lot about different things (general fears and worries)
- **Close to average / Slightly raised / High / Very high** for worries about separation from key "attachment figures" such as parents (separation anxiety)
- **Close to average / Slightly raised / High / Very high** for specific fears (specific phobia)
- **Close to average / Slightly raised / High / Very high** for social fears (social anxiety)
- **Close to average / Slightly raised / High / Very high** for panic attacks
- **Close to average / Slightly raised / High / Very high** for fears of crowds, public places, open spaces etc (agoraphobia)
- **Close to average / Slightly raised / High / Very high** for stress linked to particularly frightening events (post-traumatic stress)
- **Close to average / Slightly raised / High / Very high** for obsessions or compulsions
- **Close to average / Slightly raised / High / Very high** for depression or loss of interest
- **Close to average / Slightly raised / High / Very high** for disruptive and uncooperative behaviours (troublesome behaviour)
- **Close to average / Slightly raised / High / Very high** for antisocial or aggressive behaviours that can get people into serious trouble (troublesome behaviour)

DAWBA report template Final Version 1.1 14 Jun 2019
