# Supplementary material for: STAndardised DIagnostic Assessment for children and young people with emotional difficulties (STADIA): protocol for a multicentre randomised controlled trial
Source: BMJ Open. 2022 May 10;12(5):e053043. doi: 10.1136/bmjopen-2021-053043 (PMC9096530; doi:10.1136/bmjopen-2021-053043)
Supplement: Supplementary data [file bmjopen-2021-053043supp005.pdf]

Appendix 5. Eligible emotional disorder diagnoses

Anxiety disorder
Separation anxiety disorder
Specific phobia (any)
Social phobia or Social anxiety disorder
Agoraphobia
Panic disorder (DSM5 additionally has Panic Attack with a specifier)
Phobic anxiety disorder (unspecified)
Selective mutism
Generalized anxiety disorder
Obsessive-compulsive and related disorders
Body dysmorphic disorder
Acute stress reaction
Acute Stress Disorder
Post-traumatic stress disorder
Adjustment Disorder
Other anxiety disorder
Mixed anxiety and depressive disorder
Depression
Depressive episode (any / mild / moderate / severe)
Depressive disorder
Recurrent depressive disorder (any / mild / moderate / severe)
Major Depressive disorder
Persistent Depressive disorder
Other depressive episode
Persistent mood (affective) disorder (including cyclothymic disorder / dysthymic disorder)
Other / Unspecified mood (affective) disorder
Bipolar disorder
Bipolar affective disorder
Manic episode
Childhood emotional disorder unspecified (F93.9)
