# Supplementary material for: STAndardised DIagnostic Assessment for children and young people with emotional difficulties (STADIA): protocol for a multicentre randomised controlled trial
Source: BMJ Open. 2022 May 10;12(5):e053043. doi: 10.1136/bmjopen-2021-053043 (PMC9096530; doi:10.1136/bmjopen-2021-053043)
Supplement: Supplementary data [file bmjopen-2021-053043supp006.pdf]

STAndardised DIagnostic Assessment for children and adolescents with emotional difficulties (STADIA): A multi-centre randomised controlled trial

OUTCOME DEFINITION & ADJUDICATION PLAN

Final 1.0 – 25 February 2020

EMOTIONAL DISORDER DIAGNOSES RECORDED IN THE 12 MONTHS POST-RANDOMISATION

CONSTITUTES A CLINICAL DIAGNOSIS	REFER FOR ADJUDICATION	DOES NOT CONSTITUTE A CLINICAL DIAGNOSIS
- The presence of an eligible diagnosis within the diagnosis tab of the clinical record.<br>- The presence of an eligible diagnosis in the clinical record preceded by the heading 'diagnosis'.<br>- The presence of an eligible diagnosis in the clinical record preceded by a heading such as 'current difficulties' or 'presenting problems', except where this has been documented in the write up of the first appointment or in reference to the information received at referral (as this may simply reflect a pre-existing or referrer-made diagnosis).<br>- A clear confirmatory statement including use of an eligible diagnosis, for example:<br>*Meets the diagnostic criteria for…*<br>*Presentation is explained by a diagnosis of…*	- The presence of similar diagnostic terms within the diagnosis tab of the clinical record.<br>- The presence of an eligible diagnosis preceded by a heading such as 'current difficulties' or 'presenting problems', documented in the write up of the first appointment or in reference to the information received at referral.<br>- The presence of similar diagnostic terms preceded by a heading such as 'diagnosis', 'current difficulties' or 'presenting problems'.<br>- Reference to an eligible diagnosis or similar diagnostic terms, but where the context does not provide a clear confirmatory statement, for example:<br>*?...*<br>*Possible…*<br>*Assessed for…*<br>*…-type symptoms / behaviour*<br>*…-like symptoms / behaviour*<br>*Symptoms of…*<br>*History of…*	- No reference to an eligible diagnosis or similar diagnostic terms.<br>- A clear statement about the absence of an eligible diagnosis or similar diagnostic terms, for example:<br>*No evidence of…*<br>*…not meeting criteria for disorder*
Data collection and entry: instructions for researchers		
Use the checklist of eligible emotional disorder diagnoses.	*Document these as other emotional disorders.*	*Check 'none of the above' in the checklist of emotional disorder diagnoses and answer 'no' to 'other emotional disorder diagnoses'.*

Note: For definition of underlined terms see the Glossary below.

NO EMOTIONAL DISORDER

If there are no emotional disorder diagnoses documented in the CAMHS notes in 12 months post-randomisation, researchers will select one of the following options:

1. A clinician has documented the absence of emotional disorder.
2. Uncertainty about the presence of an emotional disorder is documented in the notes (for example, reflecting ongoing assessment / investigation).
3. There is no diagnostic information relating to emotional disorders documented in the CAMHS record.

**A clinician has documented the absence of emotional disorder.**	**Uncertainty about the presence of an emotional disorder is documented in the notes (for example, reflecting ongoing assessment / investigation).**	**There is no diagnostic information relating to emotional disorders documented in the CAMHS record.**
- Nothing in the clinical record is assessed to constitute a documented clinical diagnosis, AND<br>- There is a clear statement about the absence of one or more of the eligible diagnoses or similar diagnostic terms, for example:<br><br>*No evidence of…*<br>*…not meeting criteria for disorder*	- Nothing in the clinical record is assessed to constitute a documented clinical diagnosis, AND<br>- Reference to an eligible diagnosis or similar diagnostic terms, but where the context does not provide a clear confirmatory statement, for example:<br><br>*?...*<br>*Possible…*<br>*Assessed for…*<br>*…-type symptoms / behaviour*<br>*…-like symptoms / behaviour*<br>*Symptoms of…*<br>*History of…*	- Nothing in the clinical record is assessed to constitute a documented clinical diagnosis, AND<br>- There is no reference to an eligible diagnosis or similar diagnostic terms.*<br>- If emotional difficulties are identified they are described only by reference to the presenting symptoms with no attempt made to link these to an eligible diagnosis, for example:<br><br>*Presenting issue - Mood swings*<br>*Describing examples of ruminating thoughts.*<br><br>* Note that this includes children/young people who have not been seen by CAMHS in the 12-months post-randomisation.
Document these as absence of emotional disorder.	**REFER FOR ADJUDICATION**	**MAY REQUIRE ADJUDICATION**

**EMOTIONAL DISORDER DIAGNOSIS ADJUDICATION OUTCOME**	
The Adjudication Committee will first consider whether the record:<br>1) Constitutes a clinical diagnosis<br>2) Does not constitute a clinical diagnosis	If (1) then the Adjudication Committee will determine which of the eligible emotional disorder diagnoses apply.<br><br>If (2) then the Adjudication Committee will determine whether the record constitutes:<br>a) Absence of emotional disorder<br>b) Uncertainty about the presence of emotional disorder<br>c) No diagnostic information

Day F, *et al. BMJ Open* 2022; 12:e053043. doi: 10.1136/bmjopen-2021-053043

TREATMENTS / INTERVENTIONS GIVEN

CONSTITUTES A TREATMENT / INTERVENTION	REFER FOR ADJUDICATION
- The presence of an eligible treatment / intervention documented within the clinical record.	- Documented intervention by CAMHS where the description does not include an eligible treatment / intervention.
Data collection and entry: instructions for researchers	
Use the checklist of eligible treatments / interventions.	*Document these as other treatments / interventions.*

TREATMENTS / INTERVENTIONS ADJUDICATION OUTCOME	
The Adjudication Committee will first consider whether the record:<br>1) Constitutes a treatment / intervention<br>2) Does not constitute a treatment / intervention	If (1) then the Adjudication Committee will determine whether the record should be categorised:<br>a) As an existing treatment / intervention<br>b) As an 'other' treatment / intervention

GLOSSARY	
Eligible diagnosis	One of the pre-specified diagnoses listed on the data collection form. These should be considered present only when the exact phrase and/or corresponding ICD/DSM code is documented.
Similar diagnostic terms	Words or phrases which are similar to the eligible diagnoses, but without use of the exact wording or corresponding ICD/DSM code (e.g., separation anxiety WITHOUT use of the term disorder) or where the exact words are used alongside additional phrases (e.g., OCD-type behaviour or OCD-like symptoms).
Eligible treatment / intervention	One of the pre-specified treatments / interventions listed on the data collection form.
Adjudication Committee	The Adjudication Committee will comprise the clinician members of the Trial Management Group. A minimum of two clinicians will review terms referred for adjudication, with a third consulted if a consensus is not reached.<br>The Adjudication Committee will be blinded to treatment allocation for the purposes of adjudication.

Day F, *et al. BMJ Open* 2022; 12:e053043. doi: 10.1136/bmjopen-2021-053043
