# Supplementary material for: STAndardised DIagnostic Assessment for children and young people with emotional difficulties (STADIA): protocol for a multicentre randomised controlled trial
Source: BMJ Open. 2022 May 10;12(5):e053043. doi: 10.1136/bmjopen-2021-053043 (PMC9096530; doi:10.1136/bmjopen-2021-053043)
Supplement: Supplementary data [file bmjopen-2021-053043supp007.pdf]

Appendix 7. Summary of assessments

**Time-point**	**Maximum 10 working days from referral receipt[1]**					**6 months post-randomisation**	**12 months post-randomisation**
**Activity**	**Screening and invitation**	**Eligibility and enrolment**	**Consent and baseline**	**Randomisation**	**Intervention**<br><br>DAWBA in addition to usual practice<br><br>Or<br><br>Usual practice only	**Follow-Up**	
Initial eligibility screen of referral information	X						
Telephone invitation to participate	X						
Verbal agreement to participate		X					
Confirm eligibility		X					
Obtain enrolment data		X					
Participant enrolment		X					
Written informed consent/assent (online)			X				
Baseline demographics (parent/carer and CYP aged 16 & 17)			X				
Mood and Feelings Questionnaire (MFQ)			X			X	X
Revised Child's Anxiety Depression Scale (RCADS)			X			X	X
Strengths and Difficulties Questionnaire (SDQ)[2]			X			X	X
Child Revised Impact of Events Scale (CRIES-8)(42)[3]			X			X	X
CYP self-report self-harm measure			X			X	X
Patient Health Questionnaire (PHQ-9) - parent/carer only			X			X	X
Generalised Anxiety Disorder Assessment (GAD-7) - parent/carer only			X			X	X
Child Health Utility 9D (CHU9D)			X			X	X
EuroQol-5D youth (EQ-5D-Y)			X			X	X
EuroQol-5D five level (EQ-5D-5L)			X			X	X
Resource Use Questionnaire - parent/carer and CYP aged 16 & 17			X			X	X
Data collection from records[4]			X			X	X

[1] For sites where the waiting time for the CAMHS acceptance decision usually exceeds 10 working days from referral receipt, recruitment activities may start and/or continue beyond 10 working days from referral receipt, providing the intervention period can be completed prior to the CAMHS referral decision.
[2] For participants in the intervention arm, the baseline SDQ will be collected as part of the DAWBA, completed post-randomisation.
[3] Additional data collection undertaken to explore post-traumatic stress disorder symptoms in CYP during the Covid-19 pandemic
[4] Data collection from records will be completed periodically throughout the 12 month follow-up period.
