# Supplementary material for: STAndardised DIagnostic Assessment for children and young people with emotional difficulties (STADIA): protocol for a multicentre randomised controlled trial
Source: BMJ Open. 2022 May 10;12(5):e053043. doi: 10.1136/bmjopen-2021-053043 (PMC9096530; doi:10.1136/bmjopen-2021-053043)
Supplement: Author's manuscript [file bmjopen-2021-053043.draft_revisions.pdf]

BMJ Open

BMJ Open is committed to open peer review. As part of this commitment we make the peer review history of every article we publish publicly available.

When an article is published we post the peer reviewers' comments and the authors' responses online. We also post the versions of the paper that were used during peer review. These are the versions that the peer review comments apply to.

The versions of the paper that follow are the versions that were submitted during the peer review process. They are not the versions of record or the final published versions. They should not be cited or distributed as the published version of this manuscript.

BMJ Open is an open access journal and the full, final, typeset and author-corrected version of record of the manuscript is available on our site with no access controls, subscription charges or pay-per-view fees (http://bmjopen.bmj.com).

If you have any questions on BMJ Open's open peer review process please email info.bmjopen@bmj.com

BMJ Open

STAndardised DIagnostic Assessment for children and young people with emotional difficulties (STADIA): protocol for a multi-centre randomised controlled trial

Journal:	*BMJ Open*
Manuscript ID	bmjopen-2021-053043
Article Type:	Protocol
Date Submitted by the Author:	26-Aug-2021
Complete List of Authors:	Day, Florence; University of Nottingham,<br>Bhardwaj, Anupam; Cambridgeshire and Peterborough NHS Foundation Trust<br>Dubicka, Bernadka; The University of Manchester; Pennine Care NHS Foundation Trust<br>Ewart, Colleen<br>Gledhill, Julia; Central and North West London NHS Foundation Trust<br>James, Marilyn; University of Nottingham, School of Medicine<br>Lang, Alexandra; University of Nottingham<br>Marshall, Tamsin; Berkshire Healthcare NHS Foundation Trust<br>Montgomery, Alan; University of Nottingham<br>Reynolds, Shirley; University of Reading<br>Sprange, Kirsty; University of Nottingham, Nottingham Clinical Trials Research Unit<br>Thomson, Louise; University of Nottingham, Psychiatry<br>Bradley, Ellen; University of Nottingham<br>Lathe, James; University of Nottingham<br>Newman, Kristina; Nottinghamshire Healthcare NHS Foundation Trust<br>Partlett, Chris; University of Nottingham,<br>Starr, Kath; University of Nottingham<br>Sayal, Kapil; University of Nottingham
Keywords:	Child & adolescent psychiatry < PSYCHIATRY, Depression & mood disorders < PSYCHIATRY, HEALTH ECONOMICS

SCHOLARONE™
Manuscripts

STAndardised DIagnostic Assessment for children and young people with emotional difficulties (STADIA): protocol for a multi-centre randomised controlled trial

Authors:
Florence Day[1], Anupam Bhardwaj[2], Bernadka Dubicka[3], Colleen Ewart[4], Julia Gledhill[5], Marilyn James[1], Alexandra Lang[6], Tamsin Marshall[7], Alan Montgomery[1], Shirley Reynolds[8], Kirsty Sprange[1], Louise Thomson[9], Ellen Bradley[9], James Lathe[1], Kristina Newman[9], Chris Partlett[1], Kath Starr[1] & Kapil Sayal[9]

Corresponding author:
Professor Kapil Sayal
Institute of Mental Health
University of Nottingham
Nottingham, NG8 1BB

Email: kapil.sayal@nottingham.ac.uk

Word count, excluding title page, abstract, references, figures and tables: 3918

1 Nottingham Clinical Trials Unit, University of Nottingham, Nottingham NG7 2RD
2 Cambridge and Peterborough Foundation Trust, Brookside CAMHS, Cambridge CB2 8AH
3 Pennine Care NHS Foundation Trust, Ashton-under-Lyne OL6 7SR
4 STADIA Patient and Public Involvement co-lead, Institute of Mental Health, University of Nottingham, Nottingham NG8 1BB
5 Central and North West London NHS Foundation Trust, Harrow CAMHS, Ash Tree Clinic, South Harrow HA2 8EQ
6 Faculty of Engineering, University of Nottingham, Nottingham NG7 2RD
7 Berkshire Healthcare NHS Foundation Trust, Fitzwilliam House, Bracknell RG12 1BQ
8 School of Psychology and Clinical Language Sciences, University of Reading, Reading RG6 6DZ
9 Institute of Mental Health, University of Nottingham, Nottingham NG8 1BB

For peer review only

For peer review only - http://bmjopen.bmj.com/site/about/guidelines.xhtml

ABSTRACT

Introduction

Emotional disorders (such as anxiety and depression) are associated with considerable distress and impairment in day-to-day function for affected children and young people and for their families. Effective evidence-based interventions are available but require appropriate identification of difficulties to enable timely access to services. Standardised Diagnostic Assessment (SDA) tools may aid in the detection of emotional disorders, but there is limited evidence on the utility of SDA tools in routine care and equipoise amongst professionals about their clinical value.

Methods and analysis

A multi-centre, two-arm, parallel group RCT, with embedded qualitative and health economic components. Participants will be randomised in a 1:1 ratio to either the Development and Wellbeing Assessment (DAWBA) SDA tool as an adjunct to usual clinical care, or usual care only.

A total of 1,210 participants (Children and Young People referred to outpatient, specialist Child and Adolescent Mental Health Services (CAMHS) with emotional difficulties and their parent/carers) will be recruited from at least 6 sites in England.

The primary outcome is diagnosis of an emotional disorder within 12-months post-randomisation. Secondary outcomes include referral acceptance, diagnosis and treatment of emotional disorders, symptoms of emotional difficulties and comorbid disorders and associated functional impairment.

Ethics and dissemination

The study received favourable opinion from the South Birmingham Research Ethics Committee (Ref. 19/WM/0133). Results of this trial will be reported to the funder and published in full in the HTA Journal series and also submitted for publication in a peer reviewed journal.

Registration details

The STADIA trial was prospectively registered as ISRCTN15748675 on 29 May 2019.

Keywords

RCT; CAMHS; standardised diagnostic assessment; DAWBA; emotional disorders; diagnosis; outcomes; health economics; cost effectiveness; cost utility.

ARTICLE SUMMARY

Strengths and limitations of this study

- Large real-world multicentre randomised controlled trial of the DAWBA SDA tool as an adjunct to usual care versus usual care only.
- Trial procedures are carried out remotely with all data collection and the DAWBA completed online or via telephone, facilitating post-trial implementation into future service delivery models and routine clinical care.
- The embedded health economic component permits evaluation of both clinical and cost effectiveness.
- Embedded qualitative work will support optimal delivery and implementation to enhance acceptability, effectiveness and long-term uptake.
- Participants, researchers and clinicians cannot be blinded to treatment allocation.

For peer review only - http://bmjopen.bmj.com/site/about/guidelines.xhtml
For peer review only

INTRODUCTION

Emotional disorders cause considerable distress for affected children and young people (CYP) and their families, with adverse effects on family and peer relationships, quality of life, social involvement and activities, academic attainment and occupational opportunities, ultimately affecting life chances.[1-4] Emotional disorders are frequently comorbid with other disorders [2, 5], and are associated with self-harm and completed suicide. Effective evidence-based interventions are available but require appropriate identification of presenting difficulties to enable timely access to services and earlier recovery.[3]

The prevalence of emotional disorders has increased considerably over the past two decades.[1] In the UK, CYP with clinically significant emotional difficulties may be referred to outpatient specialist Child and Adolescent Mental Health Services (CAMHS). However, insufficient information is a common reason for referrals being declined.[6] There is limited evidence to inform optimal approaches to determine which referrals should be accepted, contributing to a large variation in acceptance rates.[6] Likewise there is a lack of evidence on how best to conduct assessments for suspected emotional difficulties to optimise outcomes. Acceptance criteria and assessment procedures differ across services and there is no single standardised approach.

The multi-disciplinary nature of CAMHS means CYP are assessed by practitioners from different professional backgrounds, with variations in training, ethos and conceptualisations of presenting difficulties. The type and scope of assessments offered vary. Assessments are often conducted by practitioners without formal diagnostic training.[7] The validity and value of mental health diagnoses have been questioned, reflecting concerns around stigma or labelling.[7-9] This can mean that in routine practice, assessments are often undertaken without the aim of making or recording a diagnosis.

However, NICE guidelines for management and treatment are usually based on diagnostic classification of disorders, so the ability to offer evidence-based interventions requires that the CYP's difficulties are appropriately identified. Although NICE Quality Standards[10] state that CYP with suspected depression should have the diagnosis confirmed and recorded, this is highly variable in practice.[7, 11] The use of diagnostic assessments has been recommended so that important problems are detected and appropriate interventions are offered.[3, 9] The NICE guidelines for depression have recommended the use of standardised diagnostic assessment (SDA) tools as potential adjuncts in the detection of depression within CAMHS.[12] It has further been recommended that SDA tools should be used as an adjunct to clinical assessments, potentially at the point of referral receipt, to enable the allocation of cases to the most appropriate professional.[8, 13, 14]

One such SDA tool is the Development and Well-Being Assessment (DAWBA), a structured package of questionnaires and interviews which can be completed online or by telephone and yields algorithm-based diagnostic information.[15] The DAWBA has established reliability and validity [15] and has been widely used for screening, diagnosis and outcome measurement in research in both clinical and community settings [16, 17], including trials of SDAs [18, 19] and large scale epidemiological research.[1, 20, 21] A previous randomised controlled trial (RCT) using the DAWBA highlighted that, for emotional disorders, disclosing DAWBA diagnosis information to clinicians can improve the level of agreement between the DAWBA and clinical diagnoses, suggesting that the DAWBA can aid clinical detection of emotional disorders.[19] It also improved detection of comorbid disorders. A UK trial found higher levels of agreement between DAWBA and clinical diagnoses, following disclosure of DAWBA information, in relation to anxiety disorders.[18] Practitioners

For peer review only - http://bmjopen.bmj.com/site/about/guidelines.xhtml

acknowledged that the additional information could supplement the assessment and aid detection of difficulties.[8]

Hence, it might be expected that the introduction of an SDA tool following CAMHS referral receipt could enable resources to be better targeted and a timely conclusion to assessments with a diagnostic decision, increase the likelihood that an appropriate evidence-based treatment is offered, and lead to improved outcomes and better experience of care for CYP and their families. However, there is limited evidence on the utility of SDA tools for informing optimal approaches to assessment within routine clinical practice.

Aims and Objectives

The aim is to evaluate the clinical and cost effectiveness of the DAWBA SDA tool, as an adjunct to usual clinical care for CYP presenting with emotional difficulties referred to CAMHS.

Specific objectives are to:

1. Conduct an RCT to determine the effectiveness of the DAWBA as an adjunct to usual clinical care on diagnosis and treatment of emotional disorders, symptoms of emotional difficulties and comorbid disorders and associated functional impairment.

2. Undertake an internal pilot to assess recruitment and acceptability.

3. Include a qualitative component within the pilot phase to address:
   a) The feasibility of recruitment.
   b) The acceptability and usability of the interventions and procedure.
   c) How the intervention is used and could be refined for the main trial.

4. Conduct a process evaluation alongside the main trial which will:
   a) Optimise the design and delivery of the DAWBA to enhance acceptability, effectiveness and long-term uptake.
   b) Identify the barriers and facilitators to implementation of the DAWBA from the perspectives of CYP, parents, and CAMHS practitioners, managers and commissioners.

5. Estimate cost effectiveness of the use of the DAWBA versus usual care.

6. Make evidence-based recommendations for assessment procedures within CAMHS and produce practice guidelines for clinical decision-making around the referral acceptance and assessment processes.

METHODS AND ANALYSIS

Design

A multi-centre, two-arm, parallel group RCT, with embedded qualitative and health economic components.

An internal pilot period, completed in the first 9 months of recruitment, will determine feasibility of recruitment and follow-up, assessed by the independent Trial Steering Committee against pre-defined stop/go criteria.

Setting

For peer review only - http://bmjopen.bmj.com/site/about/guidelines.xhtml

Recruitment will take place in at least six NHS Trusts in England, providing outpatient multidisciplinary specialist CAMHS. Sites are geographically dispersed covering urban and rural areas, thus are likely to be socio-demographically representative of CAMHS referrals in England, enabling nationally generalisable findings.

Recruitment and eligibility

Participant identification

The population is CYP presenting with emotional difficulties referred to CAMHS. Participants are identified through the usual referral pathways for the participating sites, which includes NHS and local authority managed Single/Central Point of Access referral points as well as referrals directly received and processed by CAMHS teams.

The STADIA researchers (NHS personnel, based within the CAMHS SPA/triage team to carry out research activities on behalf of the team and authorised to access referral information) at each site review the referrals received by CAMHS to identify CYP presenting with emotional difficulties, according to a standard proforma (Appendix 1. Screening form). Potentially eligible participants are invited to consider taking part in the trial and provided with written information. The initial invitation follows standardised wording to ensure clarity and consistency of approach.

Identification of participants takes place after referral receipt, but prior to referral acceptance (Figure 1).

Consent

Prior to consent, eligibility will be confirmed (**Error! Reference source not found.**) during telephone contact with the local STADIA researcher, who will also provide written and verbal information about the trial, answer questions and support the electronic consent/assent process. Participants who are eligible and provide verbal consent to participation during the call will be provided with a personal link to the online electronic Informed Consent/Assent Form (Table 2), enabling them to provide written informed consent/assent.

Table 1. Eligibility criteria

Inclusion criteria for the CYP<br>• Aged 5 to 17 years.<br>• Referred to outpatient multidisciplinary specialist CAMHS.<br>• Presenting with emotional difficulties.<br>• If aged <16, has an eligible individual with parental responsibility (see parent/carer eligibility criteria below) willing and able to participate in the trial.<br>• If aged 16-17, has capacity to provide valid written informed consent.<br>• If aged 16-17 and participating without a parent/carer, able to complete the assessment tool in English.<br>• If aged 16-17 and participating without a parent/carer, access to internet and email or telephone.
Exclusion criteria for the CYP<br>• Emergency or urgent referral to outpatient multidisciplinary specialist CAMHS (i.e. requires an expedited assessment) according to local risk assessment procedures.<br>• Severe learning disability.<br>• Previously randomised in the STADIA trial.
Inclusion criteria for the parent/carer

For peer review only - http://bmjopen.bmj.com/site/about/guidelines.xhtml

- Individual with parental responsibility for the CYP referred to CAMHS; this will be the CYP's mother or father, legally appointed guardian or a person with a residence order concerning the CYP.
- Adequate knowledge of the CYP to be able to complete the assessment tool (i.e., known for at least 6 months).
- Has capacity to provide valid written informed consent.
- Access to internet and email or telephone.
- Able to complete the assessment tool in English.

Exclusion criteria for the parent/carer

- Local authority representatives designated to care for the CYP.

The participation and consent/assent requirements for the trial are shown in Table 2.

Table 2: Consent & Participation

WHO WAS REFERRED TO CAMHS?	CYP aged <11	CYP aged 11-15		CYP aged 16-17	
WHO IS INITIALLY CONTACTED?	Parent/carer			Depends on contact details provided with the CAMHS referral*	
WHO CONSENTS?	Parent/carer	Parent/carer	Parent/carer	CYP AND parent/carer (optional)	CYP
WHO ASSENTS?	None	CYP (optional)	None	None	None
WHO ARE THE PARTICIPANTS?	Parent/carer only	CYP and parent/carer dyad	Parent/carer only	CYP and parent/carer dyad	CYP only
WHO IS THE PRIMARY PARTICIPANT?**	Parent/carer	Parent/carer	Parent/carer	CYP	CYP
WHO IS THE SECONDARY PARTICIPANT?	None	CYP	Non	Parent/carer	None
WHO IS INVITED TO COMPLETE THE DAWBA?	Parent/carer	Parent/carer AND CYP	Parent/carer	CYP AND parent/carer	CYP
WHO IS INVITED TO COMPLETE RESEARCH QUESTIONNAIRES?	Parent/carer report on CYP<br>Parent/carer self-report	Parent/carer report on CYP<br>Parent/carer self-report<br>CYP self-report	Parent/carer report on CYP<br>Parent/carer self-report	CYP self-report<br>Parent/carer report on CYP<br>Parent/carer self-report	CYP self-report

For all CYP aged <16 the initial contact about the study will be with the parent/carer. The involvement of CYP aged 11-15 will be at the discretion of the parent/carer.
* For CYP aged 16-17 if the CYP's contact details are provided on the CAMHS referral the first contact about the study will be with the CYP who can choose to nominate a parent/carer to participate in the trial alongside them or participate alone. If the parent/carer's contact details only are available the first contact will be with the parent/carer and the parent/carer will be asked

For peer review only - http://bmjopen.bmj.com/site/about/guidelines.xhtml

whether the CYP can also be contacted but may choose to refuse this. The parent/carer will not be able to participate in the STADIA trial without the involvement or consent of the CYP.
** The primary participant is the person who must provide consent as a minimum requirement in order for randomisation to take place. Assent (of CYP aged 11-15) and parental consent (for CYP aged 16 and 17) may also be sought but is not mandatory and therefore will not be required prior to randomisation.

Participants are free to withdraw at any time and for any reason. Participants may withdraw from the intervention, follow-up questionnaires and/or data collection from records in any combination (e.g., participants who do not complete the intervention will continue to be followed-up, participants withdrawing from follow-up questionnaire completion may continue to consent for data collection from records). Withdrawn participants will not be replaced. Data collected prior to withdrawal will be retained and used in the analysis.

Where CYP aged 16 or 17 have consented for their own involvement they can continue to participate in the trial in the event of their parent/carer's withdrawal, however, the parent/carer involvement would not continue should the CYP withdraw consent.

Randomisation and concealment
Participants will be randomised in a 1:1 ratio to either intervention or control. Allocation will be assigned using a minimisation algorithm balancing on recruiting site, CYP age (5-10, 11-15, 16-17 years) and sex, incorporating a probabilistic element to allocation. The allocation algorithm was created by Nottingham Clinical Trials Unit (NCTU) in accordance with their Standard Operating Procedures (SOPs). Allocation is concealed using an automated web system operated by NCTU.

Randomisation is automatically generated within the online system following submission, and automated verification, of baseline data by the primary participant. Participants are presented with their allocation and further instructions on-screen with email confirmation. Instructions for DAWBA completion are included for those in the intervention arm. Email confirmation is sent to the coordinating centre and site research team.

It will not be possible to blind participants, clinicians and some trial staff to treatment allocation, but treatment allocation data will be restricted to those trial staff who require access to facilitate trial conduct.

The risk of contamination between arms is considered low. Access to the DAWBA, and provision of the DAWBA report, is only provided to participants in the intervention arm. SDA tools are not current practice in standard care and it is unlikely that control participants will be asked to complete these at the point of referral receipt. DAWBA completion occurring outside the trial for control arm participants will be collected during follow-up.

Interventions
Development and wellbeing assessment (DAWBA)
The trial intervention is the DAWBA. [22] The DAWBA has a modular structure, with only those modules relevant to emotional and comorbid disorders included (Table 3). No freetext responses are collected.

Table 3: DAWBA modules

DAWBA Module	Included in STADIA-specific DAWBA report?

For peer review only - http://bmjopen.bmj.com/site/about/guidelines.xhtml

Strengths and Difficulties Questionnaire	Does not generate diagnostic predictions so not included in the DAWBA report
Separation Anxiety	Yes
Specific Phobia	Yes
Social Phobia	Yes
Panic and Agoraphobia	Yes
Generalised Anxiety	Yes
Post-traumatic stress disorder (PTSD)	Yes
Obsessive compulsive disorder (OCD)	Yes
Depression	Yes
Bipolar disorder	Does not generate diagnostic predictions so not included in the DAWBA report
Body dysmorphic disorder (BDD)	Does not generate diagnostic predictions so is not included in the DAWBA report
Oppositional defiant disorder (ODD)	Yes
Conduct Disorder	Yes

The DAWBA will be self-reported by participants via the secure, standalone online platform created and maintained by the DAWBA developer.[22] Access is by a unique ID number and password, assigned at the point of randomisation via a stock control system integrated into the randomisation system, ensuring accountability of DAWBA slot allocation.

The DAWBA may be completed by the parent/carer and/or CYP aged 11+, depending on the consent and participation arrangements (Table 2). DAWBA completion will be monitored and the STADIA researcher will support and encourage completion. Participants will be able to complete the DAWBA in a telephone call with the STADIA researcher if required. Participants are asked to complete all modules of the DAWBA presented to them. Should the DAWBA be only partially completed by respondents the report will be based only on fully answered modules with missing responses identified as such.

A trial-specific DAWBA report will be prepared for each participant, based on a standard, study-specific template (***Error! Reference source not found.***). The algorithm-derived diagnostic predictions will be used to highlight the likelihood of a CYP meeting ICD-10 criteria for the disorders assessed; the report is based entirely on the algorithm-derived predictions and is not clinically rated. The report will be sent to participants (via post or email) and CAMHS clinicians (via upload to the clinical record), as an adjunct to usual clinical practice.

Control

CYP randomised to the control arm will receive usual care (i.e., referral review as usual). Based on standard information provided with the referral a clinical decision is made about whether the referral is accepted and, if so, a clinician conducts the initial CAMHS assessment as per usual practice in the service.

Sample size

A target sample size of 1210 participants will be recruited and randomised, with equal allocation to intervention or control.

Assuming 45% of control participants have a confirmed diagnosis within 12 months (based on unpublished data obtained from the trial sites), detection of an absolute increase of 10% with 90% power and 5% two-sided alpha, requires 544 participants per arm for analysis. Allowing for up to 10% non-collection of the primary outcome, we will randomise 1210 participants.

For peer review only - http://bmjopen.bmj.com/site/about/guidelines.xhtml

Measures and outcomes

Primary outcome

The primary outcome is a clinician-made diagnosis decision about the presence of an emotional disorder within 12 months of randomisation. Diagnosis of an emotional disorder will be coded as 'yes'; absence or uncertainty (for example, reflecting ongoing assessment or investigation) will be coded as 'no'. Eligible diagnoses are those that reflect 'emotional' or 'internalizing' disorders in ICD/DSM (Appendix 3. Eligible emotional disorder diagnoses). The diagnosis must be documented in the clinical record within 12 months of randomisation by a mental health services clinician in an NHS-delivered or NHS-commissioned service.

Diagnoses will be collected from clinical records using a standard proforma. Alternative possible diagnoses identified from the clinical notes will be recorded verbatim on the data capture form and will be subject to adjudication by members of the Trial Management Group (**Error! Reference source not found.**).

Secondary outcomes

Secondary outcomes are listed in
and further detailed in **Error! Reference source not found.**.

Table 4. Secondary outcomes

Outcome	Measurement
Acceptance of index referral	Collected from records
Acceptance of any referral within 12 months of randomisation	Collected from records
Discharge from CAMHS within 12 months	Collected from records
Re-referral to CAMHS within 12 months	Collected from records
Confirmed diagnosis decision	Collected from records
Time from randomisation to diagnosis of emotional disorder	Collected from records
Diagnoses made over the 12 month period from randomisation	Collected from records
Treatment offered for diagnosed emotional disorder	Collected from records
Any treatment / interventions given	Collected from records
Time from randomisation to the decision to offer treatment for a diagnosed emotional disorder	Collected from records
Time from randomisation to start of first treatment for a diagnosed emotional disorder	Collected from records
Time from randomisation to the decision to offer any treatment	Collected from records
Time from randomisation to start of any treatment	Collected from records
Participant-reported diagnoses received from CAMHS in the 12 months post-randomisation	Participant self-report
Depression symptoms (CYP)	Mood and Feelings Questionnaire (MFQ)[23]
Anxiety symptoms (CYP)	Revised Child's Anxiety Depression Scale (RCADS)[24]
Oppositional defiant / conduct disorder symptoms (CYP)	Strengths and Difficulties Questionnaire (SDQ)[25]

For peer review only - http://bmjopen.bmj.com/site/about/guidelines.xhtml

Functional Impairment (CYP)	Strengths and Difficulties Questionnaire (SDQ)[25]
Self-harm thoughts (CYP)	CYP self-report self-harm measure
Self-harm behaviour (CYP)	CYP self-report self-harm measure
Depression symptoms (parent/carer)	Patient Health Questionnaire (PHQ-9)[26]
Anxiety symptoms (parent/carer)	Generalised Anxiety Disorder Assessment (GAD-7)[27]
Time off education, employment or training because of emotional difficulties for the CYP	Resource use questionnaire

Health economic measures

Outcomes
Health related quality of life (HRQoL) of the CYP assessed using the Child Health Utility 9D (CHU9D)[28] and EuroQol-5D youth (EQ-5D-Y).[29] These measures will be self-reported by CYP aged 11 and over, with proxy versions also completed by the parent/carer for CYP <16.

HRQoL for the parent/carer assessed using the EuroQol-5D five level version (EQ-5D-5L).[30]

Resource Use
Data will be collected on health care, education, and social care resource use for both the CYP and parents/carers, using a purposely designed resource use collection tool. The questionnaire was developed by health economists, in tandem with feedback from PPI representatives, addressing primary, secondary, and social care costs, alongside the broader patient-borne costs. These data will be attributable to the emotional difficulties of the young person and be self-reported by the parent/carer with supplementary information obtained from CYP aged 16 and 17. Administrative records of treatments/interventions offered by CAMHS during the trial period may be considered as a supplementary source of data.

Data collection
Data will be collected through participant reported questionnaires (parent/carer and CYP self-report aged 11+) and from clinical records. Participant reported outcomes will be collected at baseline and 6- and 12-months post-randomisation (**Error! Reference source not found.**). Questionnaires are intended to be completed online by participants in the first instance - to maximise rates of completion and retention there will be an option for telephone completion, should participants have difficulty accessing or completing the questionnaires online.

Outcomes collected from records will be reported for the 12-month period following randomisation.

Data management and analysis
Data management
Arrangements for data handling are specified in the Data Management Plan (DMP). Central and on-site monitoring will be carried out as required following a risk assessment and as documented in the monitoring plan. Monitoring activities will be carried out by the coordinating centre on behalf of the trial sponsor.

Data will be held on servers located within The University of Nottingham data centres. Security is both physical (secure limited access) and electronic (behind firewalls, access via user accounts). Personal data recorded on all documents will be regarded as strictly confidential and handled and stored in accordance with the Data Protection Act 2018.

For peer review only - http://bmjopen.bmj.com/site/about/guidelines.xhtml

Statistical analysis
The primary approach to between-group comparative analyses will be by modified intention-to-treat (i.e. including all participants who have been randomised and without imputation of missing outcome data).

The primary comparative analysis will employ a generalised linear mixed model to compare the proportions in each group with a clinician-made diagnosis decision within 12 months of randomisation, adjusted for minimisation variables. The comparison will be presented as both an absolute (risk difference) and relative (risk ratio) effect, along with 95% confidence intervals.

Secondary outcomes will be analysed using appropriate mixed effect regression models dependent on data type and will adjust for factors used in the minimisation and baseline value of the outcome where measured. For outcomes measured at multiple time points, these will be analysed using a mixed model with a treatment by time interaction to obtain estimates of treatment effect at each follow-up time.

Appropriate interaction terms will be included in the primary regression analyses in order to conduct subgroup analyses according to sex and age of the CYP.

Health economic analysis
In accordance with NICE guidance, primary analysis will take an NHS and personal social services perspective. Unit costs will be attached to participant reports of health care resource use or recorded treatments/interventions offered by CAMHS. The cost of the DAWBA itself will be distributed at the participant-level across the intervention arm of the trial. Sensitivity analyses will take a wider perspective to capture the broader societal costs inclusive of out-of-pocket expenses and productivity losses. Indices of HRQoL for the EQ-5D, EQ-5D-Y, and CHU9D will be derived using relevant population tariffs, and quality adjusted life years estimated using area under the curve (AUC).

The economic evaluation will take an incremental approach between the two groups using an intention-to-treat (ITT) population (irrespective of treatment received) and a 12-month time horizon. The outcome for the primary cost utility analysis will be the joint young person and parent/carer QALYs. The outcome for the secondary cost effectiveness analysis will be confirmed diagnosis decisions. Outcomes will be paired with their respective direct-to-NHS costs, bootstrapped, and scattered on the cost effectiveness plane to characterise the uncertainty in incremental estimates. Using the net monetary benefit framework,[31] Cost Effectiveness Acceptability Curves (CEACs) will be constructed to show the non-parametric probability the intervention is a cost effective option, compared to usual care, across a range of willingness to pay thresholds per QALY, and within the secondary analysis per confirmed diagnosis decision. While the receipt of any diagnosis of emotional difficulties in young people would likely lead to large divergences in lifecourse outcomes, the heterogeneity of conditions considered for diagnosis (**Error! Reference source not found.**) renders CUA modelling across the lifecourse infeasible. Secondary analysis is expected to be fully captured within the 12-month time horizon.

A full statistical analysis plan (SAP) and health economics analysis plan (HEAP) will be developed and agreed prior to database lock and un-blinding of the analysing statistician and health economist.

Embedded qualitative study
During the internal pilot, semi-structured interviews are undertaken with a sample of participants who consented to be invited to participate in qualitative interviews. Researchers, clinicians, service

For peer review only - http://bmjopen.bmj.com/site/about/guidelines.xhtml

managers and commissioners are identified by site leads. The proposed sample size is 25 participants (parent/carer and CYP aged 16-17), 25 staff and 15 service managers and commissioners. Interviews address: a) the feasibility of recruitment; b) the acceptability and usability of the interventions and procedure; c) how the intervention is used and how this deployment could be refined for the main trial. Interviews are conducted by the qualitative researcher (KN) in person, or by phone or video call based on participant preferences and pandemic restrictions.

A process evaluation, conducted during the main trial phase, will aim to identify the barriers and facilitators to implementation of the intervention. Semi-structured interviews will be conducted with a further sample of participants and clinicians to explore the perceived functioning of the intervention, the organisation of the service and reflective experiences on outcomes.

Qualitative interview data will be recorded and encrypted on a password protected Dictaphone and transferred securely to medical transcription company Dict8 for transcription. Transcriptions will be anonymised. Audio files will be destroyed after transcripts have been checked. Anonymised transcriptions will be analysed and stored on password protected computers and the secure University of Nottingham server.

Qualitative analysis

All qualitative interview data will be initially analysed by the qualitative researcher (KN) using interpretative thematic approaches to coding, and adopt the framework method,[32] with input from the qualitative lead (LT), Chief Investigator (KSa) and PPI leads (CE & AL). NVIVO 12 will be used to manage the qualitative data.

Patient and public involvement

Prior to submission, the proposal was informed by consultations with a person with lived parent/carer experience of CAMHS, including contribution to and review of the proposal, recruitment strategy, participant trial experience and consideration of burden of the intervention, and establishing a PPI workstream.

Following award, the PPI Co-I team recruited two representatives naïve of the study design to provide independent review of the trial via their membership of the Trial Steering Committee (TSC). Both TSC members are persons with lived parent/carer experience of CAMHS.

During study set up, PPI Co-I expertise was utilised to support researcher recruitment via the design and deployment of role plays within interviews.[33] This was to gain insight into candidates' capabilities when dealing with sensitive and challenging participant scenarios. Additionally, they contributed to design of researcher training materials, to support standardised approaches across trial sites. Iterative and creative design PPI activities were integral in the development of the STADIA trial logo and branding to ensure accessibility and acceptability to CYP and parents.

Since study commencement participatory design approaches have seen PPI co-design of the resource use questionnaire, qualitative interviews and the protocol for a Study Within A Trial (SWAT) to support participant engagement with follow-up. Additionally, collaborative working between the PPI and Qualitative workstreams has enabled examination of the qualitative themes using principles of the Framework Method[32] for independent verification of those themes.

Two PPI advisory panels have been established, meeting on average every 3 months since month 9 of the study. "STADIA PPI Panel" has 8 adult members, with lived parent/carer experience of CAMHS. "STADIA Labs" has 6 CYP members, aged 15 to 19 at inception, with lived experience of CAMHS. These groups have been involved in many traditional activities such as review of PIS and

For peer review only - http://bmjopen.bmj.com/site/about/guidelines.xhtml

consent forms, consultation on language and content for participant reminder text messages. PPI co-production activities are also seeing the development of age appropriate study newsletters and the design of STADIA information videos including decision making about video concept, audience, message, aesthetic and content. PPI group members are provided with supplementary training about PPI practices and involvement opportunities. Due to the Covid-19 pandemic, PPI meetings have had to move online and so the PPI team are investing in knowledge transfer and upskilling PPI representatives in different ways of working and collaborating online.

There are a range of planned flexible opportunities for participating in project feedback and dissemination activities including co-facilitating and presenting at the interactive dissemination workshop / consensus meeting, publication authorship as peer researcher and presenting at conferences to showcase the project findings.

ETHICS AND DISSEMINATION

Ethics

The study was reviewed and received favourable opinion from the South Birmingham Research Ethics Committee (Ref. 19/WM/0133) on 12 June 2019; subsequent amendments have been approved. The current, approved protocol is version 3.0 dated 13 August 2020.

Safety

The trial intervention is conceptually similar to usual clinical practice (i.e., CYP referred to CAMHS may be sent questionnaires about their difficulties), therefore the risks of the trial are considered comparable. The DAWBA is widely used in research for data collection therefore, although utilised as an intervention in the STADIA trial, the risks may be regarded as similar to those of an observational/questionnaire study. Data to inform safety oversight will therefore be collected during routine follow-up, from existing outcome measures. There is no separate adverse event or serious adverse event reporting.

The number of participants meeting pre-defined safety outcomes will be reported on an ongoing basis to the Trial Management Group (TMG) and TSC. Data will be presented by arms to the Data Monitoring Committee (DMC).

Trial oversight

Nottinghamshire Healthcare NHS Foundation Trust will undertake role of Sponsor as defined by the UK Policy Framework for Health and Social Care Research.[34] Delegated responsibilities will be assigned to the Chief Investigator, participating NHS Trusts and the trial coordinating centre, Nottingham Clinical Trials Unit (NCTU).

The full co-applicant team and NCTU staff responsible for the day-to-day management of the trial will form the TMG, responsible for monitoring recruitment and retention rates and implementing strategies to ensure targets are met. Independent Trial Steering and Data Monitoring Committees will operate in accordance with trial-specific Charters.

Dissemination

Results of this trial will be reported to the funder and published in full in the HTA Journal series and also submitted for publication in a peer reviewed journal.

Data Sharing

For peer review only

For peer review only - http://bmjopen.bmj.com/site/about/guidelines.xhtml

Anonymised trial data may be shared with researchers external to the trial research team in accordance with the NCTU's data sharing procedure.

Figures

Figure 1: Participant flow

Authors' contributions

All authors made substantial contributions to conception and design, acquisition of data, or analysis and interpretation of data; took part in drafting the article or revising it critically for important intellectual content; agreed to submit to the current journal; gave final approval of the version to be published; and agree to be accountable for all aspects of the work.

Funding statement

This study was funded as a result of a commissioned call by the National Institute for Health Research (NIHR) Health Technology Assessment programme (Grant Reference Number 16/96/09). The views expressed are those of the authors and not necessarily those of the NIHR or the Department of Health and Social Care.

The funder will have no role in the collection, management, analysis, and interpretation of data; writing of the report; and the decision to submit the report for publication.

Competing interests

The authors declare no competing interests.

Acknowledgements

We would like to acknowledge and thank all the parent/carers and young people participating in the trial and the research sites involved in recruiting participants and data collection. The authors would also like to thank the wider STADIA team for their input, including the PPI Advisory Panels, members of the independent Trial Steering and Data Monitoring Committees, and the Nottingham Clinical Trials Unit, who are the trial coordinating centre.

Finally, thanks to the trial sponsor, Nottinghamshire Healthcare NHS Foundation Trust (research@nottshc.nhs.uk).

For peer review only - http://bmjopen.bmj.com/site/about/guidelines.xhtml

REFERENCES

1. Sadler, K., et al. *Mental Health of Children and Young People in England, 2017*. 2018 06 February 2019]; Available from: https://digital.nhs.uk/data-and-information/publications/statistical/mental-health-of-children-and-young-people-in-england/2017/2017.
2. Goodyer IM, R.S., Barrett B, Byford S, Dubicka B, Hill J, Holland F, Kelvin R, Midgley N, Roberts C, Senior R, Target M, Widmer B, Wilkinson P, Fonagy P, *Cognitive behavioural therapy and short-term psychoanalytic psychotherapy versus brief psychosocial intervention in adolescents with unipolar major depression (IMPACT): a multicentre, pragmatic, observer-blind, randomised controlled trial.* Health Technology Assessment, 2017. **21**(12).
3. Simmons, M., P. Wilkinson, and B. Dubicka, *Measurement Issues: Depression measures in children and adolescents.* Child and Adolescent Mental Health, 2015. **20**(4): p. 230-241.
4. Colman, I.W., MEJ. Croudace, TJ. Jones, PB., *Forty-Year Psychiatric Outcomes Following Assessment for Internalizing Disorder in Adolescence.* American Journal of Psychiatry, 2007. **164**(1): p. 126-133.
5. Orchard, F., et al., *Clinical characteristics of adolescents referred for treatment of depressive disorders.* Child and Adolescent Mental Health, 2017. **22**(2): p. 61-68.
6. Frith, E. *Access and waiting times in children and young people's mental health services.* 2017 08 January 2019]; Available from: https://epi.org.uk/wp-content/uploads/2018/01/EPI_Access-and-waiting-times_.pdf.
7. Michelson, D., et al., *Improving psychiatric diagnosis in multidisciplinary child and adolescent mental health services.* The Psychiatrist, 2011. **35**: p. 454-459.
8. Martin, A.-M., et al., *Practitioners' attitudes towards the use of standardized diagnostic assessment in routine practice: A qualitative study in two Child and Adolescent Mental Health Services.* Clinical Child Psychology and Psychiatry, 2011. **16**(3): p. 407-420.
9. Craddock, N., et al., *Wake-up call for British psychiatry.* British Journal of Psychiatry, 2008. **193**(1): p. 6-9.
10. National Institute for Health and Clinical Excellence. *Depression in Children and Young People: Quality Standard*. 2013 08 January 2019]; Available from: https://www.nice.org.uk/guidance/cg28.
11. Fitzpatrick C, A.N.-N., Kehoe A, Devlin N, Glackin S, Power L, et al, *Do we miss depressive disorders and suicidal behaviours in clinical practice.* Clinical Child Psychology and Psychiatry, 2012. **17**: p. 449-458.
12. National Institute for Health and Clinical Excellence. *Depression in Children and Young People: Identification and Management in Primary, Community and Secondary Care – Update*. 2015 08 January 2019]; Available from: https://www.nice.org.uk/guidance/cg28.
13. Last, A., Henley, W., Norman, S., Goodman, R., Ford, T., *Innovations in Practice: Feasibility of the development and well-being assessment as an adjunct to clinical assessment in child and adolescent mental health services. .* Child and Adolescent Mental Health, 2014. **19**: p. 142-146.
14. Reeves, K., E. Charter, and T. Ford, *Measurement Issues: Is standardised diagnostic assessment feasible as an adjunct to clinical practice? A systematic review.* Child and Adolescent Mental Health, 2016. **21**(1): p. 51-63.
15. Goodman, R., et al., *The Development and Well-Being Assessment: Description and Initial Validation of an Integrated Assessment of Child and Adolescent Psychopathology.* Journal of Child Psychology and Psychiatry, 2000. **41**(5): p. 645-655.
16. Hall, C.L., et al., *Investigating a therapist-guided, parent-assisted remote digital behavioural intervention for tics in children and adolescents—'Online Remote Behavioural Intervention for Tics' (ORBIT) trial: protocol of an internal pilot study and single-blind randomised controlled trial.* BMJ Open, 2019. **9**(1): p. e027583.

For peer review only - http://bmjopen.bmj.com/site/about/guidelines.xhtml

17. Pickles, A., et al., *Parent-mediated social communication therapy for young children with autism (PACT): long-term follow-up of a randomised controlled trial.* The Lancet, 2016. **388**(10059): p. 2501-2509.
18. Ford, T., et al., *Can standardized diagnostic assessment be a useful adjunct to clinical assessment in child mental health services? A randomized controlled trial of disclosure of the Development and Well-Being Assessment to practitioners.* Social Psychiatry and Psychiatric Epidemiology, 2013. **48**(4): p. 583-593.
19. Aebi, M., et al., *The use of the development and well-being assessment (DAWBA) in clinical practice: a randomized trial.* European child & adolescent psychiatry, 2012. **21**(10): p. 559-567.
20. Meltzer, H., et al. *The mental health of children and adolescents in Great Britain*. 2000 07 February 2019]; Available from: http://webarchive.nationalarchives.gov.uk/20160105160709/http://www.ons.gov.uk/ons/rel/psychiatric-morbidity/the-mental-health-of-children-and-adolescents-in-great-britain/1999-survey/mental-health-of-children-and-adolescents-in-great-britain.pdf.
21. Green, H., et al. *Mental health of children and young people in Great Britain, 2004*. 2005 07 February 2019]; Available from: https://webarchive.nationalarchives.gov.uk/20180307185414/http://digital.nhs.uk/catalogue/PUB06116.
22. Youth in Mind. *Information for researchers and clinicians about the Development and Well-Being Assessment*. 2012 08 January 2019]; Available from: http://www.dawba.info/.
23. Angold, A., et al., *Development of a short questionnaire for use in epidemiological studies of depression in children and adolescents.* International Journal of Methods in Psychiatric Research, 1995. **5**(4): p. 237-249.
24. Chorpita, B.F., et al., *Assessment of symptoms of DSM-IV anxiety and depression in children: a revised child anxiety and depression scale.* Behaviour Research and Therapy, 2000. **38**(8): p. 835-855.
25. Goodman, R., *The Extended Version of the Strengths and Difficulties Questionnaire as a Guide to Child Psychiatric Caseness and Consequent Burden.* Journal of Child Psychology and Psychiatry, 1999. **40**(5): p. 791-799.
26. Kroenke, K., R.L. Spitzer, and J.B. Williams, *The PHQ-9: validity of a brief depression severity measure.* Journal of general internal medicine, 2001. **16**(9): p. 606-613.
27. Spitzer, R.L., et al., *A brief measure for assessing generalized anxiety disorder: The gad-7.* Archives of Internal Medicine, 2006. **166**(10): p. 1092-1097.
28. Stevens, K., *Developing a descriptive system for a new preference-based measure of health-related quality of life for children.* Quality of Life Research, 2009. **18**(8): p. 1105-1113.
29. Wille, N., et al., *Development of the EQ-5D-Y: a child-friendly version of the EQ-5D.* Qual Life Res, 2010. **19**(6): p. 875-86.
30. Herdman, M., et al., *Development and preliminary testing of the new five-level version of EQ-5D (EQ-5D-5L).* Quality of Life Research, 2011. **20**(10): p. 1727-1736.
31. Hoch, J.S., A.H. Briggs, and A.R. Willan, *Something old, something new, something borrowed, something blue: a framework for the marriage of health econometrics and cost-effectiveness analysis.* Health Economics, 2002. **11**(5): p. 415-430.
32. Gale, N.K., et al., *Using the framework method for the analysis of qualitative data in multi-disciplinary health research.* BMC Medical Research Methodology, 2013. **13**(1): p. 117.
33. Furniss, D., A.R. Lang, and C. Ewart, *Enhancing Patient Ergonomics with Patient and Public Involvement in Research Projects.* , in *The Patient Factor: A Handbook on Patient Ergonomics*, R.J. Holden and R.S. Valdez, Editors. 2001, CRC Press.
34. Health Research Authority. *UK Policy Framework for Health and Social Care Research*. 2017 18 December 2018]; Available from: https://www.hra.nhs.uk/planning-and-improving-research/policies-standards-legislation/uk-policy-framework-health-social-care-research/

For peer review only - http://bmjopen.bmj.com/site/about/guidelines.xhtml

35. Wood, A., et al., *Properties of the mood and feelings questionnaire in adolescent psychiatric outpatients: a research note.* 1995. **36**: p. 327-334.
36. Daviss, W.B., et al., *Criterion validity of the Mood and Feelings Questionnaire for depressive episodes in clinic and non-clinic subjects.* 2006. **47**: p. 927-934.
37. Chorpita, B.F., C.E. Moffitt, and J. Gray, *Psychometric properties of the Revised Child Anxiety and Depression Scale in a clinical sample.* Behaviour Research and Therapy, 2005. **43**(3): p. 309-322.
38. Yao, S., et al., *Measuring Adolescent Psychopathology: Psychometric Properties of the Self-Report Strengths and Difficulties Questionnaire in a Sample of Chinese Adolescents.* Journal of Adolescent Health, 2009. **45**(1): p. 55-62.
39. Goodman, R., *Psychometric Properties of the Strengths and Difficulties Questionnaire.* Journal of the American Academy of Child & Adolescent Psychiatry, 2001. **40**(11): p. 1337-1345.
40. Muris, P., C. Meesters, and F. van den Berg, *The Strengths and Difficulties Questionnaire (SDQ).* European child & adolescent psychiatry, 2003. **12**(1): p. 1-8.
41. Lundh, L.G., Wangby-Lundh, M., & Bjarehed, J. , *Self reported emotional and behavioral problems in Swedish 14 to 15-year-old adolescents: A study with the self-report version of the Strengths and Difficulties Questionnaire.* Scandinavian Journal of Psychology, 2008. **49**: p. 523-532.
42. Perrin, S., R. Meiser-Stedman, and P. Smith, *The children's revised impact of event scale (CRIES): Validity as a screening instrument for PTSD.* Behavioural and Cognitive Psychotherapy, 2005. **33**(4): p. 487-498.

For peer review only

For peer review only - http://bmjopen.bmj.com/site/about/guidelines.xhtml

Figure 1: Participant flow

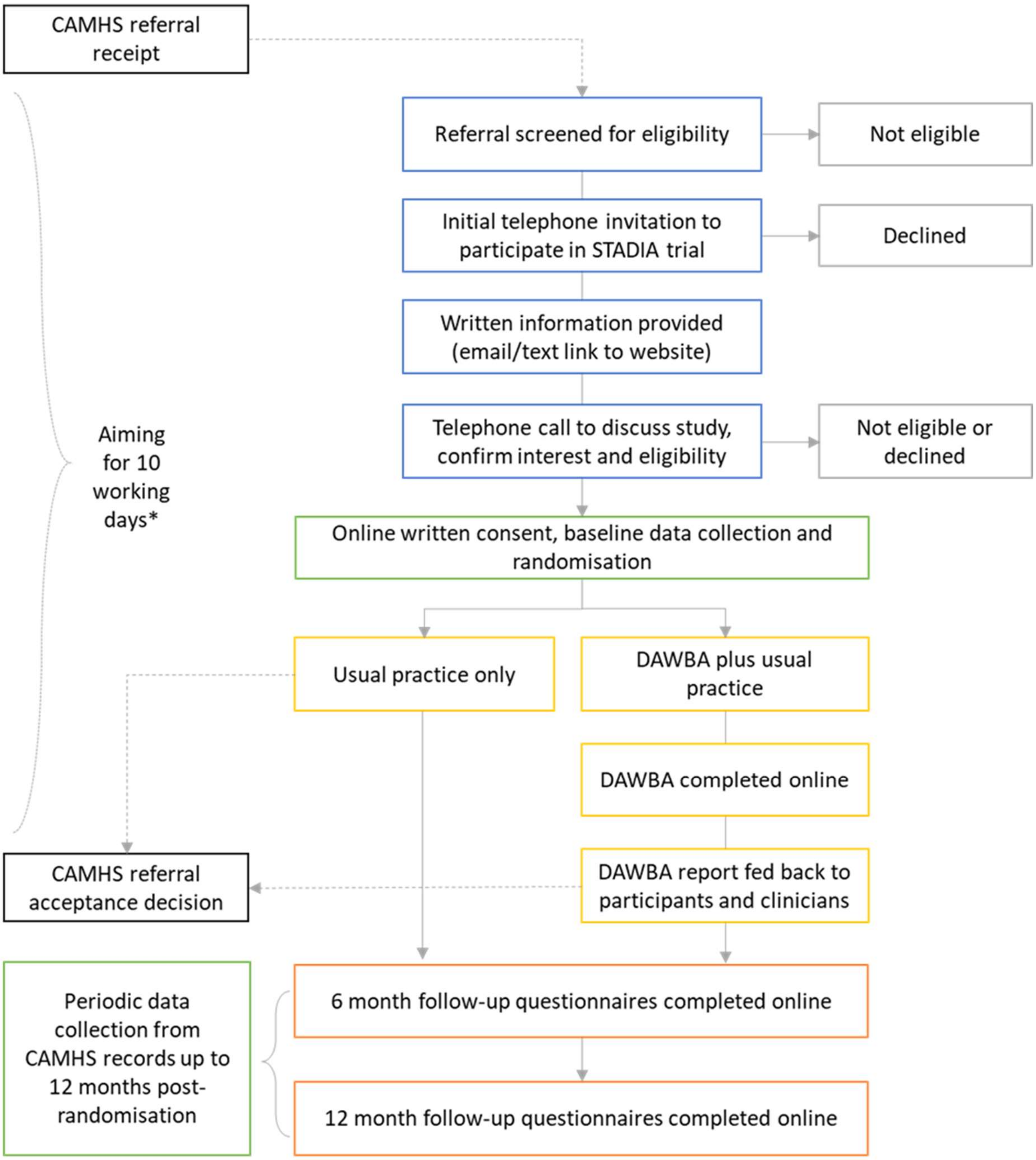

** For sites where the waiting time for the CAMHS acceptance decision usually exceeds 10 working days from referral receipt, recruitment activities may start and/or continue beyond 10 working days from referral receipt, providing the intervention period can be completed prior to the CAMHS referral decision.*

For peer review only

STAndardised DIagnostic Assessment for children and adolescents with emotional difficulties (STADIA): a multi-centre randomised controlled trial

SCREENING

**Site Number:**	
**Screening Number:**	
**Sponsor:**	**Nottinghamshire Healthcare NHS Foundation Trust**
**CRF Version:**	**Final v1.1 – 30 April 2019**

peer review only

Site Number: ☐☐

Screening Number: ☐☐☐

REFERRAL SCREENING	
Complete for all referrals screened for eligibility:	
NHS Number<br>*Local use only*	______________________
Trust Number<br>*Local use only*	______________________
Date of referral receipt (dd-mmm-yyyy)	☐☐ - ☐☐☐ - ☐☐☐☐
Date of screening (dd-mmm-yyyy)	☐☐ - ☐☐☐ - ☐☐☐☐
Young person's sex	Male ☐<br>Female ☐
Young person's age<br>*If <5 or >17 do not proceed*	☐☐
Has the young person been previously enrolled and randomised in the STADIA trial?<br>*If yes, do not proceed*	Yes ☐<br>No ☐
Does the referral mention any of the following Covid-19 related words/phrases?<br>*Tick all that apply.*<br>Covid-19 / Covid<br>Coronavirus<br>Lockdown<br>School closure / exams cancelled	 <br>☐<br>☐<br>☐<br>☐
Does the referral mention emotional difficulties*?<br>*If no, do not proceed*	Yes ☐<br>No ☐
Is this an emergency or urgent referral (according to local CAMHS triage / SPA team risk assessment)?<br>*If yes, do not proceed*	Yes ☐<br>No ☐
Does the young person have severe learning disability (e.g., the referral mentions this or that they attend a special school for children with severe learning difficulties)?<br>*If 'yes' do not proceed*<br>*If not known, confirm during telephone eligibility check at enrolment*	Yes ☐<br>No ☐<br>Not known ☐

For peer review only - http://bmjopen.bmj.com/site/about/guidelines.xhtml

For peer review only

Site Number: ☐☐

Screening Number: ☐☐☐

REFERRAL SCREENING	
If the young person is <16:<br>Does the referral information include contact details for a named parent/carer?<br>*If 'no' await parent/carer contact details before proceeding*	Yes ☐<br>No ☐<br>N/A ☐
If the young person is <16:<br>Is the named parent/carer a local authority representative designated to care for the child/young person?<br>*If 'yes' do not proceed*<br>*If not known, confirm during telephone eligibility check at enrolment*	Yes ☐<br>No ☐<br>Not known ☐<br>N/A ☐
If the young person is aged 16 or 17:<br>Whose contact details are given on the referral form?<br>*If young person contact details are provided, they should be contacted in the first instance*	Young person ☐<br>Parent/carer ☐<br>Both ☐<br>N/A ☐

EMOTIONAL DIFFICULTIES	
**Emotional difficulties may be indicated by the use of any of the following key words or phrases.*<br>*Tick all that apply. If 'other' record details and seek advice from the PI or NCTU before proceeding.*	
None	☐
Agitated / agitation	☐
Anger	☐
Anxiety / anxious / generalised anxiety	☐
Avoids things/people/places	☐
Can't leave the house	☐
Completing rituals / asking parents to carry out rituals	☐
Compulsions	☐
Depressed / depression / low / low mood / sad	☐
Difficulties sleeping	☐
Distress	☐
Fears and worries / fears relating to safety (germs, fire)	☐
Feeling low	☐
Feels flat / empty / blank	☐

For peer review only - http://bmjopen.bmj.com/site/about/guidelines.xhtml
For peer review only

Site Number: | | |

Screening Number: | | | |

EMOTIONAL DIFFICULTIES	
**Emotional difficulties may be indicated by the use of any of the following key words or phrases.<br>Tick all that apply. If 'other' record details and seek advice from the PI or NCTU before proceeding.*	
Feels hopeless	☐
Feels worthless / stupid	☐
Flashbacks	☐
Hypervigilance	☐
Irritable	☐
Low motivation	☐
Low self-esteem / Hates self	☐
Mood swings / moody	☐
Negative thoughts	☐
Nightmares (if trauma also present)	☐
No (or loss of) energy	☐
No (or loss of) interest in things / gave up… / lack of wanting to do things	☐
Not going to school / unable to go to school	☐
Not sleeping / poor sleep	☐
Obsessions	☐
OCD	☐
Phobia	☐
Panic / panic attacks	☐
PTSD	☐
Self-harm / DSH / Cutting	☐
Suicidal	☐
Suicidal thoughts / thoughts of ending life / thinks about killing self	☐
Tearful	☐
Thoughts of death	☐

For peer review only

For peer review only - http://bmjopen.bmj.com/site/about/guidelines.xhtml

Site Number: | | |

Screening Number: | | | |

EMOTIONAL DIFFICULTIES	
**Emotional difficulties may be indicated by the use of any of the following key words or phrases. Tick all that apply. If 'other' record details and seek advice from the PI or NCTU before proceeding.*	
Tiredness / fatigue	☐
Touching objects	☐
Trauma	☐
Weepy	☐
Withdrawal / withdrawn	☐
Worried / worrying (incl. worries/concerns about their appearance	☐
Other (please specify)	☐

FOR ALL REFERRALS SCREENED, ENTER SUMMARY DATA ON THE SCREENING & ENROLMENT LOG.

IF THE YOUNG PERSON APPEARS TO BE ELIGIBLE PROCEED TO THE INVITATION TELEPHONE CALL (CALL 1) AND ENTER DETAILS ON THE TRIAL DATABASE.

SIGN-OFF STATEMENT	
Completed by the researcher conducting the referral screening.	
To the best of my knowledge, I confirm that I have made every reasonable effort to ensure that ALL of the data in this Case Record Form is a true, accurate and complete report.	
Print Name	
Signature	
Date	_ _ - _ _ _ - _ _ _ _

For peer review only - http://bmjopen.bmj.com/site/about/guidelines.xhtml

DAWBA Report

The DAWBA collects information about a range of common emotional and behavioural difficulties, and uses this information to produce a report to highlight the level of difficulties.

How to understand the ratings

These ratings compare your responses with the responses from large numbers of other parents and young people across the UK. Many parents and young people find this sort of comparison helpful, but it is just a guide and not the same as a face-to-face assessment with a specialist.

To make it easier to read, we have grouped the ratings into four categories. Each category is different. This shows how your [child's] *(delete as appropriate)* difficulties compare with other children / young people:

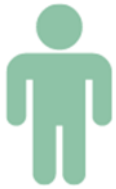

Close to average

In the general population most children/ young people (roughly 80 out of 100) are in the "close to average" category.

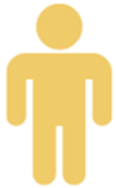

Slightly raised

If the ratings are in the "slightly raised" category this means the difficulties are slightly higher than average. Roughly 10 out of 100 children / young people are in this category.

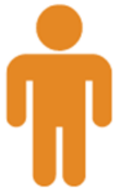

High

Around 5 in 100 children / young people score in the "high" category. This means that the difficulties are more severe than average.

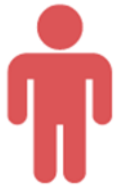

Very high

Around 5 in 100 children score in the "very high" category. This means that the difficulties appear to be more severe than we find in 95 out of every 100 children / young people.

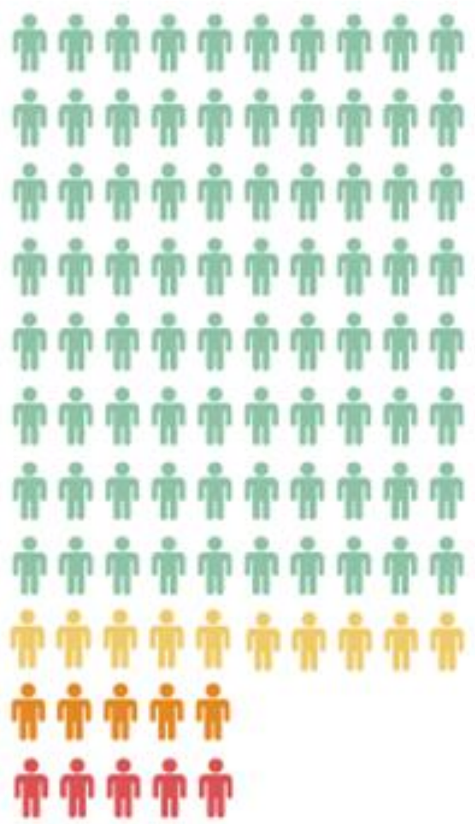

For peer review

The rating is only a rough guide. As high ratings can be a "false alarm", please use your own judgement. Not all difficulties need treating. Some difficulties get better by themselves, particularly if they are mild or if they have only been there for a short time.

Most strengths and difficulties lie on a scale. There will be children / young people at each end of the scale but most children / young people will fall somewhere in between.

For peer review only - http://bmjopen.bmj.com/site/about/guidelines.xhtml

Your [child's] *(delete as appropriate)* **ratings:**

- **Close to average / Slightly raised / High / Very high** for worrying a lot about different things (general fears and worries)
- **Close to average / Slightly raised / High / Very high** for worries about separation from key "attachment figures" such as parents (separation anxiety)
- **Close to average / Slightly raised / High / Very high** for specific fears (specific phobia)
- **Close to average / Slightly raised / High / Very high** for social fears (social anxiety)
- **Close to average / Slightly raised / High / Very high** for panic attacks
- **Close to average / Slightly raised / High / Very high** for fears of crowds, public places, open spaces etc (agoraphobia)
- **Close to average / Slightly raised / High / Very high** for stress linked to particularly frightening events (post-traumatic stress)
- **Close to average / Slightly raised / High / Very high** for obsessions or compulsions
- **Close to average / Slightly raised / High / Very high** for depression or loss of interest
- **Close to average / Slightly raised / High / Very high** for disruptive and uncooperative behaviours (troublesome behaviour)
- **Close to average / Slightly raised / High / Very high** for antisocial or aggressive behaviours that can get people into serious trouble (troublesome behaviour)

For peer review only

For peer review only - http://bmjopen.bmj.com/site/about/guidelines.xhtml

APPENDICES

Appendix 1. Screening form

Appendix 2. Template DAWBA report

Appendix 3. Eligible emotional disorder diagnoses

Anxiety disorder
Separation anxiety disorder
Specific phobia (any)
Social phobia or Social anxiety disorder
Agoraphobia
Panic disorder (DSM5 additionally has Panic Attack with a specifier)
Phobic anxiety disorder (unspecified)
Selective mutism
Generalized anxiety disorder
Obsessive-compulsive and related disorders
Body dysmorphic disorder
Acute stress reaction
Post-traumatic stress disorder
Adjustment Disorder
Other anxiety disorder
Mixed anxiety and depressive disorder
Depression
Depressive episode (any / mild / moderate / severe)
Depressive disorder
Recurrent depressive disorder (any / mild / moderate / severe)
Major Depressive disorder
Persistent Depressive disorder
Other depressive episode
Persistent mood (affective) disorder (including cyclothymic disorder / dysthymic disorder)
Other / Unspecified mood (affective) disorder
Bipolar disorder
Bipolar affective disorder
Manic episode
Childhood emotional disorder unspecified (F93.9)

Appendix 4. Outcome Definition and Adjudication Plan

For peer review only - http://bmjopen.bmj.com/site/about/guidelines.xhtml

Appendix 5. Secondary outcome definitions

Outcome	Measurement	Definition
Acceptance of index referral	Collected from records	Whether the index referral (i.e., the referral made to CAMHS at the point of recruitment to the STADIA trial) was accepted or declined.<br>Acceptance is defined as being offered an appointment within CAMHS, whether or not the initial appointment was attended or subsequent appointments were offered/attended.
Acceptance of any referral within 12 months of randomisation	Collected from records	Whether the index referral or any subsequent referral to CAMHS (if made) was accepted or not.<br>Acceptance as defined above for index referral.
Discharge from CAMHS within 12 months	Collected from records	Whether the CYP was discharged from CAMHS (following acceptance of the index referral) during the 12-months post-randomisation.
Re-referral to CAMHS within 12 months	Collected from records	Whether the CYP was re-referred to CAMHS (for those whose index referral was turned down by CAMHS or those whose index referral was accepted but were subsequently discharged) during the 12-months post-randomisation.
Confirmed diagnosis decision	Collected from records	Diagnosis of an emotional disorder or confirmed absence of an emotional disorder coded as 'yes' vs. uncertainty about the presence of an emotional disorder coded as 'no'.<br>Diagnosis as defined for primary outcome.
Time from randomisation to diagnosis of emotional disorder	Collected from records	Date of diagnosis will be the first documented eligible diagnosis.<br>Diagnosis as defined for primary outcome.
Diagnoses made over the 12 month period from randomisation	Collected from records	The diagnosis must be documented in the clinical record within 12 months of randomisation by a mental health services clinician in an NHS-delivered or NHS-commissioned service.<br>All diagnoses made within 12 months will be included.
Treatment offered for diagnosed emotional disorder	Collected from records	Whether treatment was offered for a diagnosed emotional disorder, as defined for primary outcome.
Any treatment / interventions given	Collected from records	All treatments/interventions offered by CAMHS for any reason within 12 months of randomisation, whether or not there is a documented diagnosis will be included.
Time from randomisation to the decision to offer treatment for a diagnosed emotional disorder	Collected from records	Date of decision will be the first date that the decision to offer treatment for a diagnosed emotional disorder is documented in the clinical notes.

For peer review only - http://bmjopen.bmj.com/site/about/guidelines.xhtml

Outcome	Measurement	Definition
Time from randomisation to start of first treatment for a diagnosed emotional disorder	Collected from records	Date of treatment will be the first date that any treatment offered for a diagnosed emotional disorder is started.<br><br>Treatment and diagnosed emotional disorder as defined.
Time from randomisation to the decision to offer any treatment	Collected from records	Date of decision will be the first date that the decision to offer any treatment is documented in the clinical notes.
Time from randomisation to start of any treatment	Collected from records	Date of treatment will be the first date that any treatment offered is started.<br><br>Treatment as defined.
Participant-reported diagnoses received in the 12 months post-randomisation	Participant self-report	Participants will be asked to report whether or not they received a diagnosis of the CYP's difficulties from CAMHS in the 12 months post-randomisation and if so, what diagnosis was given and by whom.
Depression symptoms in the CYP	Mood and Feelings Questionnaire (MFQ)	Mood and Feelings Questionnaire (MFQ) [23]<br>MFQ is a valid and reliable measure of depression in CYP.[35, 36]<br><br>33-items are answered on a 3-point scale ("not true" = 0, "somewhat true" = 1 point, "true" = 2 points).<br><br>Scores range from 0 to 66 with higher scores indicating more severe depressive symptoms.<br>A score of 27 or higher may be indicative of depression.

For peer review only - http://bmjopen.bmj.com/site/about/guidelines.xhtml

Outcome	Measurement	Definition
Anxiety symptoms in the CYP	Revised CYP's Anxiety Depression Scale (RCADS)	Revised CYP's Anxiety and Depression Scale (RCADS)[24]<br>RCADS is a 47-item questionnaire that measures the reported frequency of various symptoms of anxiety and low mood. Each item is rated on a 4-point scale (never = 0, sometimes = 1, often = 2, always = 3).<br>An overall anxiety and low mood score is generated, with separate sub-scale scores for separation anxiety, social phobia, generalised anxiety, panic, obsessive compulsive disorder and major depression.<br>RCADS demonstrates good psychometric properties.[37]<br>Total anxiety and depression scores range from 0 to 141.<br><br>We will record scores for each of the 6 sub-scales. For analysis metric, we will use the total anxiety score.
Comorbid oppositional defiant / conduct disorder symptoms in the CYP	Strengths and Difficulties Questionnaire (SDQ)	Strengths & Difficulties Questionnaire (SDQ):[25] A 25-item emotional and behavioural screening questionnaire for CYP.<br><br>Each item is rated on a 3-point scale (not true, somewhat true, certainly true). Values of 0, 1 or 2 are assigned to each response.<br><br>SDQ comprises 5 sub-scales and an impact supplement. The impact supplement asks effect of difficulties on homelife, friendships, education and leisure activities.<br><br>SDQ has demonstrated reasonable psychometric properties.[38-41]<br>Scores on the 'conduct problems' subscale will be used in the analysis of this outcome.<br><br>Sub-scale scores range from 0 to 10.
Functional Impairment in the CYP	Strengths and Difficulties Questionnaire (SDQ)	Impact supplement scores will be used to determine functional impairment. Impact scores range from 0 to 10.

For peer review only - http://bmjopen.bmj.com/site/about/guidelines.xhtml

Outcome	Measurement	Definition
Self-harm thoughts in the CYP	CYP self-report self-harm measure	CYP will be asked to report the frequency of thoughts of self-harm.<br>Frequency of thoughts of self-harm are rated over the last 6 months in the following categories and scored accordingly:<br>Not at all (0)<br>Once or twice (1)<br>Three or more times (2)
Self-harm behaviour in the CYP	CYP self-report self-harm measure	CYP will be asked to report frequency of instances of self-harm behaviour.<br>Frequency of self-harm behaviour are rated over the last 6 months in the following categories and scored accordingly:<br>Not at all (0)<br>Once (1)<br>Two or more times (2)
Depression symptoms in the parent/carer	Patient Health Questionnaire (PHQ-9)	PHQ-9:[26] PHQ-9 is frequently used as a screening tool for depression in general populations. Each of the nine DSM-IV depression criteria are scored as "0" (not at all) to "3" (nearly every day) depending on the frequency with which they were experienced over the last 2 weeks.<br>Total scores range from 0 to 27 with higher scores indicating increased severity of depression.
Anxiety symptoms in the parent/carer	Generalised Anxiety Disorder Assessment (GAD-7)	GAD-7:[27]<br>GAD-7 is a measure of the severity of anxiety in general populations. 7 items are rated according to the frequency with which they have been experienced over the past 2 weeks (0 = 'not at all', 1 = 'several days', 2 = 'more than half the days', and 3 = 'nearly every day').<br>Total scores range from 0 to 21 with higher scores indicating more severe anxiety.
Time off education, employment or training because of emotional difficulties for the CYP	Resource use questionnaire	Days missed from education, employment or training (as applicable) for the CYP due to emotional difficulties.

For peer review only - http://bmjopen.bmj.com/site/about/guidelines.xhtml

Appendix 6. Summary of assessments

Time-point	Maximum 10 working days from referral receipt[1]					6 months post-randomisation	12 months post-randomisation
**Activity**	**Screening and invitation**	**Eligibility and enrolment**	**Consent and baseline**	**Randomisation**	**Intervention**<br>DAWBA in addition to usual practice<br>Or<br>Usual practice only	**Follow-Up**	
Initial eligibility screen of referral information	X						
Telephone invitation to participate	X						
Verbal agreement to participate		X					
Confirm eligibility		X					
Obtain enrolment data		X					
Participant enrolment		X					
Written informed consent/assent (online)			X				
Baseline demographics (parent/carer and CYP aged 16 & 17)			X				
Mood and Feelings Questionnaire (MFQ)			X			X	X
Revised Child's Anxiety Depression Scale (RCADS)			X			X	X
Strengths and Difficulties Questionnaire (SDQ)[2]			X			X	X
Child Revised Impact of Events Scale (CRIES-8)[42][3]			X			X	X
CYP self-report self-harm measure			X			X	X
Patient Health Questionnaire (PHQ-9) - parent/carer only			X			X	X
Generalised Anxiety Disorder Assessment (GAD-7) - parent/carer only			X			X	X
Child Health Utility 9D (CHU9D)			X			X	X
EuroQol-5D youth (EQ-5D-Y)			X			X	X
EuroQol-5D five level (EQ-5D-5L)			X			X	X
Resource Use Questionnaire - parent/carer and CYP aged 16 & 17			X			X	X
Data collection from records[4]			X			X	X

[1] For sites where the waiting time for the CAMHS acceptance decision usually exceeds 10 working days from referral receipt, recruitment activities may start and/or continue beyond 10 working days from referral receipt, providing the intervention period can be completed prior to the CAMHS referral decision.
[2] For participants in the intervention arm, the baseline SDQ will be collected as part of the DAWBA, completed post-randomisation.
[3] Additional data collection undertaken to explore post-traumatic stress disorder symptoms in CYP during the Covid-19 pandemic
[4] Data collection from records will be completed periodically throughout the 12 month follow-up period.

For peer review only

For peer review only - http://bmjopen.bmj.com/site/about/guidelines.xhtml

STAndardised DIagnostic Assessment for children and adolescents with emotional difficulties (STADIA): A multi-centre randomised controlled trial

OUTCOME DEFINITION & ADJUDICATION PLAN

Final 1.0 – 25 February 2020

EMOTIONAL DISORDER DIAGNOSES RECORDED IN THE 12 MONTHS POST-RANDOMISATION

CONSTITUTES A CLINICAL DIAGNOSIS	REFER FOR ADJUDICATION	DOES NOT CONSTITUTE A CLINICAL DIAGNOSIS
- The presence of an eligible diagnosis within the diagnosis tab of the clinical record.<br>- The presence of an eligible diagnosis in the clinical record preceded by the heading 'diagnosis'.<br>- The presence of an eligible diagnosis in the clinical record preceded by a heading such as 'current difficulties' or 'presenting problems', except where this has been documented in the write up of the first appointment or in reference to the information received at referral (as this may simply reflect a pre-existing or referrer-made diagnosis).<br>- A clear confirmatory statement including use of an eligible diagnosis, for example:<br>*Meets the diagnostic criteria for…*<br>*Presentation is explained by a diagnosis of…*	- The presence of similar diagnostic terms within the diagnosis tab of the clinical record.<br>- The presence of an eligible diagnosis preceded by a heading such as 'current difficulties' or 'presenting problems', documented in the write up of the first appointment or in reference to the information received at referral.<br>- The presence of similar diagnostic terms preceded by a heading such as 'diagnosis', 'current difficulties' or 'presenting problems'.<br>- Reference to an eligible diagnosis or similar diagnostic terms, but where the context does not provide a clear confirmatory statement, for example:<br>*?...*<br>*Possible…*<br>*Assessed for…*<br>*…-type symptoms / behaviour*<br>*…-like symptoms / behaviour*<br>*Symptoms of…*<br>*History of…*	- No reference to an eligible diagnosis or similar diagnostic terms.<br>- A clear statement about the absence of an eligible diagnosis or similar diagnostic terms, for example:<br>*No evidence of…*<br>*…not meeting criteria for disorder*
Data collection and entry: instructions for researchers		
Use the checklist of eligible emotional disorder diagnoses.	*Document these as other emotional disorders.*	*Check 'none of the above' in the checklist of emotional disorder diagnoses and answer 'no' to 'other emotional disorder diagnoses'.*

Note: For definition of underlined terms see the Glossary below.

For peer review only - http://bmjopen.bmj.com/site/about/guidelines.xhtml

NO EMOTIONAL DISORDER

If there are no emotional disorder diagnoses documented in the CAMHS notes in 12 months post-randomisation, researchers will select one of the following options:

1. A clinician has documented the <u>absence</u> of emotional disorder.
2. <u>Uncertainty</u> about the presence of an emotional disorder is documented in the notes (for example, reflecting ongoing assessment / investigation).
3. There is no diagnostic information relating to emotional disorders documented in the CAMHS record.

A clinician has documented the <u>absence</u> of emotional disorder.	<u>Uncertainty</u> about the presence of an emotional disorder is documented in the notes (for example, reflecting ongoing assessment / investigation).	There is <u>no diagnostic information</u> relating to emotional disorders documented in the CAMHS record.
- Nothing in the clinical record is assessed to constitute a documented clinical diagnosis, AND<br>- There is a clear statement about the absence of one or more of the <u>eligible diagnoses</u> or <u>similar diagnostic terms</u>, for example:<br><br>*No evidence of…*<br>*…not meeting criteria for disorder*	- Nothing in the clinical record is assessed to constitute a documented clinical diagnosis, AND<br>- Reference to an <u>eligible diagnosis</u> or <u>similar diagnostic terms</u>, but where the context does not provide a clear confirmatory statement, for example:<br><br>*?...*<br>*Possible…*<br>*Assessed for…*<br>*…-type symptoms / behaviour*<br>*…-like symptoms / behaviour*<br>*Symptoms of…*<br>*History of…*	- Nothing in the clinical record is assessed to constitute a documented clinical diagnosis, AND<br>- There is no reference to an <u>eligible diagnosis</u> or <u>similar diagnostic terms</u>.*<br>- If emotional difficulties are identified they are described only by reference to the presenting symptoms with no attempt made to link these to an eligible diagnosis, for example:<br><br>*Presenting issue - Mood swings*<br>*Describing examples of ruminating thoughts.*<br><br>* Note that this includes children/young people who have <u>not been seen by CAMHS</u> in the 12-months post-randomisation.
Document these as absence of emotional disorder.	REFER FOR ADJUDICATION	MAY REQUIRE ADJUDICATION

For peer review only

<table>
<tr><th colspan="2">EMOTIONAL DISORDER DIAGNOSIS ADJUDICATION OUTCOME</th></tr>
<tr><td rowspan="2">The Adjudication Committee will first consider whether the record:
1) Constitutes a clinical diagnosis
2) Does not constitute a clinical diagnosis</td><td>If (1) then the Adjudication Committee will determine which of the eligible emotional disorder diagnoses apply.</td></tr>
<tr><td>If (2) then the Adjudication Committee will determine whether the record constitutes:
a) Absence of emotional disorder
b) Uncertainty about the presence of emotional disorder
c) No diagnostic information</td></tr>
</table>

For peer review only - http://bmjopen.bmj.com/site/about/guidelines.xhtml

TREATMENTS / INTERVENTIONS GIVEN

CONSTITUTES A TREATMENT / INTERVENTION	REFER FOR ADJUDICATION
- The presence of an eligible treatment / intervention documented within the clinical record.	- Documented intervention by CAMHS where the description does not include an eligible treatment / intervention.
Data collection and entry: instructions for researchers	
Use the checklist of eligible treatments / interventions.	*Document these as other treatments / interventions.*

TREATMENTS / INTERVENTIONS ADJUDICATION OUTCOME	
The Adjudication Committee will first consider whether the record:<br>1) Constitutes a treatment / intervention<br>2) Does not constitute a treatment / intervention	If (1) then the Adjudication Committee will determine whether the record should be categorised:<br>a) As an existing treatment / intervention<br>b) As an 'other' treatment / intervention

GLOSSARY	
Eligible diagnosis	One of the pre-specified diagnoses listed on the data collection form. These should be considered present only when the exact phrase and/or corresponding ICD/DSM code is documented.
Similar diagnostic terms	Words or phrases which are similar to the eligible diagnoses, but without use of the exact wording or corresponding ICD/DSM code (e.g., separation anxiety WITHOUT use of the term disorder) or where the exact words are used alongside additional phrases (e.g., OCD-type behaviour or OCD-like symptoms).
Eligible treatment / intervention	One of the pre-specified treatments / interventions listed on the data collection form.
Adjudication Committee	The Adjudication Committee will comprise the clinician members of the Trial Management Group. A minimum of two clinicians will review terms referred for adjudication, with a third consulted if a consensus is not reached.<br>The Adjudication Committee will be blinded to treatment allocation for the purposes of adjudication.

For peer review only - http://bmjopen.bmj.com/site/about/guidelines.xhtml

BMJ Open

STAndardised DIagnostic Assessment for children and young people with emotional difficulties (STADIA): protocol for a multi-centre randomised controlled trial

Journal:	*BMJ Open*
Manuscript ID	bmjopen-2021-053043.R1
Article Type:	Protocol
Date Submitted by the Author:	25-Feb-2022
Complete List of Authors:	Day, Florence; University of Nottingham, Nottingham Clinical Trials Unit, School of Medicine<br>Wyatt, Laura; University of Nottingham, Nottingham Clinical Trials Unit, School of Medicine<br>Bhardwaj, Anupam; Cambridgeshire and Peterborough NHS Foundation Trust<br>Dubicka, Bernadka; The University of Manchester; Pennine Care NHS Foundation Trust<br>Ewart, Colleen; University of Nottingham, STADIA Patient and Public Involvement co-lead, Institute of Mental Health<br>Gledhill, Julia; Central and North West London NHS Foundation Trust<br>James, Marilyn; University of Nottingham, Nottingham Clinical Trials Unit, School of Medicine<br>Lang, Alexandra; University of Nottingham, Faculty of Engineering<br>Marshall, Tamsin; Berkshire Healthcare NHS Foundation Trust<br>Montgomery, Alan; University of Nottingham, Nottingham Clinical Trials Unit, School of Medicine<br>Reynolds, Shirley; University of Reading, School of Psychology and Clinical Language Sciences<br>Sprange, Kirsty; University of Nottingham, Nottingham Clinical Trials Unit, School of Medicine<br>Thomson, Louise; University of Nottingham, Institute of Mental Health, School of Medicine<br>Bradley, Ellen; University of Nottingham, Institute of Mental Health, School of Medicine<br>Lathe, James; University of Nottingham, Nottingham Clinical Trials Unit, University of Nottingham<br>Newman, Kristina; Nottinghamshire Healthcare NHS Foundation Trust, Institute of Mental Health<br>Partlett, Chris; University of Nottingham, Nottingham Clinical Trials Unit, School of Medicine<br>Starr, Kath; University of Nottingham, Nottingham Clinical Trials Unit, School of Medicine<br>Sayal, Kapil; University of Nottingham, Institute of Mental Health, School of Medicine; Nottinghamshire Healthcare NHS Foundation Trust, Institute of Mental Health
**Primary Subject Heading**:	Mental health

Secondary Subject Heading:	Mental health
Keywords:	Child & adolescent psychiatry < PSYCHIATRY, Depression & mood disorders < PSYCHIATRY, HEALTH ECONOMICS

SCHOLARONE™
Manuscripts

STAndardised DIagnostic Assessment for children and young people with emotional difficulties (STADIA): protocol for a multi-centre randomised controlled trial

Authors:

Florence Day[1*], Laura Wyatt[1*], Anupam Bhardwaj[2], Bernadka Dubicka[3], Colleen Ewart[4], Julia Gledhill[5], Marilyn James[1], Alexandra Lang[6], Tamsin Marshall[7], Alan Montgomery[1], Shirley Reynolds[8], Kirsty Sprange[1], Louise Thomson[9], Ellen Bradley[9], James Lathe[1], Kristina Newman[10], Chris Partlett[1], Kath Starr[1] & Kapil Sayal[9,10]

*joint first authors

Corresponding author:

Professor Kapil Sayal
Institute of Mental Health
School of Medicine
University of Nottingham
Nottingham, NG8 1BB

Email: kapil.sayal@nottingham.ac.uk

Word count, excluding title page, abstract, references, figures and tables: 4339

1 Nottingham Clinical Trials Unit, School of Medicine, University of Nottingham, Nottingham NG7 2RD
2 Cambridge and Peterborough Foundation Trust, Brookside CAMHS, Cambridge CB2 8AH
3 Pennine Care NHS Foundation Trust, Ashton-under-Lyne OL6 7SR
4 STADIA Patient and Public Involvement co-lead, Institute of Mental Health, University of Nottingham, Nottingham NG8 1BB
5 Central and North West London NHS Foundation Trust, Harrow CAMHS, Ash Tree Clinic, South Harrow HA2 8EQ
6 Faculty of Engineering, University of Nottingham, Nottingham NG7 2RD
7 Berkshire Healthcare NHS Foundation Trust, Fitzwilliam House, Bracknell RG12 1BQ
8 School of Psychology and Clinical Language Sciences, University of Reading, Reading RG6 6DZ
9 Institute of Mental Health, School of Medicine, University of Nottingham, Nottingham NG8 1BB
10 Nottinghamshire Healthcare NHS Foundation Trust, Institute of Mental Health, Nottingham NG8 1BB

For peer review only - http://bmjopen.bmj.com/site/about/guidelines.xhtml

ABSTRACT

Introduction
Emotional disorders (such as anxiety and depression) are associated with considerable distress and impairment in day-to-day function for affected children and young people and for their families. Effective evidence-based interventions are available but require appropriate identification of difficulties to enable timely access to services. Standardised Diagnostic Assessment (SDA) tools may aid in the detection of emotional disorders, but there is limited evidence on the utility of SDA tools in routine care and equipoise amongst professionals about their clinical value.

Methods and analysis
A multi-centre, two-arm, parallel group RCT, with embedded qualitative and health economic components. Participants will be randomised in a 1:1 ratio to either the Development and Wellbeing Assessment (DAWBA) SDA tool as an adjunct to usual clinical care, or usual care only.

A total of 1,210 participants (Children and Young People referred to outpatient, specialist Child and Adolescent Mental Health Services (CAMHS) with emotional difficulties and their parent/carers) will be recruited from at least 6 sites in England.

The primary outcome is a clinician-made diagnosis about the presence of an emotional disorder within 12-months of randomisation. Secondary outcomes include referral acceptance, diagnosis and treatment of emotional disorders, symptoms of emotional difficulties and comorbid disorders and associated functional impairment.

Ethics and dissemination
The study received favourable opinion from the South Birmingham Research Ethics Committee (Ref. 19/WM/0133). Results of this trial will be reported to the funder and published in full in the HTA Journal series and also submitted for publication in a peer reviewed journal.

Registration details
The STADIA trial was prospectively registered as ISRCTN15748675 on 29 May 2019.

Keywords
RCT; CAMHS; standardised diagnostic assessment; DAWBA; emotional disorders; diagnosis; outcomes; health economics; cost effectiveness; cost utility.

ARTICLE SUMMARY

Strengths and limitations of this study
- Large real-world multicentre randomised controlled trial of the DAWBA SDA tool as an adjunct to usual care versus usual care only.
- Trial procedures are carried out remotely with all data collection and the DAWBA completed online or via telephone, facilitating post-trial implementation into future service delivery models and routine clinical care.
- The embedded health economic component permits evaluation of both clinical and cost effectiveness.
- Embedded qualitative work will support optimal delivery and implementation to enhance acceptability, effectiveness and long-term uptake.
- Participants, researchers and clinicians cannot be blinded to treatment allocation.

For peer review only - http://bmjopen.bmj.com/site/about/guidelines.xhtml

INTRODUCTION

Emotional disorders cause considerable distress for affected Children and Young People (CYP) and their families, with adverse effects on family and peer relationships, quality of life, social involvement and activities, academic attainment and occupational opportunities, ultimately affecting life chances.(1-4) Emotional disorders are frequently comorbid with other disorders (2, 5), and are associated with self-harm and completed suicide. Effective evidence-based interventions are available but require appropriate identification of presenting difficulties to enable timely access to services and earlier recovery.(3)

The prevalence of emotional disorders has increased considerably over the past two decades.(1) In the UK, CYP with clinically significant emotional difficulties may be referred to outpatient specialist Child and Adolescent Mental Health Services (CAMHS). However, insufficient information is a common reason for referrals being declined.(6) There is limited evidence to inform optimal approaches to determine which referrals should be accepted, contributing to a large variation in acceptance rates.(6) Likewise there is a lack of evidence on how best to conduct assessments for suspected emotional difficulties to optimise outcomes. Acceptance criteria and assessment procedures differ across services and there is no single standardised approach.

The multi-disciplinary nature of CAMHS means CYP are assessed by practitioners from different professional backgrounds, with variations in training, ethos and conceptualisations of presenting difficulties. The type and scope of assessments offered vary. Assessments are often conducted by practitioners without formal diagnostic training(7) and recording of potential diagnostic information can be influenced by patient, clinician and service related contextual considerations(8). The validity and value of mental health diagnoses have been questioned, reflecting concerns around restricting service access (9), stigma or labelling.(7, 10, 11) This can mean that in routine practice, assessments are often undertaken without the aim of making or recording a diagnosis.

However, NICE guidelines for management and treatment are usually based on diagnostic classification of disorders, so the ability to offer evidence-based interventions requires that the CYP's difficulties are appropriately identified. Although NICE Quality Standards(12) state that CYP with suspected depression should have the diagnosis confirmed and recorded, this is highly variable in practice.(7, 13) The use of diagnostic assessments has been recommended so that important problems are detected and appropriate interventions are offered.(3, 11) The NICE guidelines for depression have recommended the use of standardised diagnostic assessment (SDA) tools as potential adjuncts in the detection of depression within CAMHS.(14) It has further been recommended that SDA tools should be used as an adjunct to clinical assessments, potentially at the point of referral receipt, to enable the allocation of cases to the most appropriate professional.(10, 15, 16)

One such SDA tool is the Development and Well-Being Assessment (DAWBA), a structured package of questionnaires and interviews which can be completed online or by telephone and yields algorithm-based diagnostic information.(17) The DAWBA has established reliability and validity (17) and has been widely used for screening, diagnosis and outcome measurement in research in both clinical and community settings (18, 19), including trials of SDAs (20, 21) and large scale epidemiological research.(1, 22, 23) A previous randomised controlled trial (RCT) using the DAWBA highlighted that, for emotional disorders, disclosing DAWBA diagnosis information to clinicians can improve the level of agreement between the DAWBA and clinical diagnoses, suggesting that the DAWBA can aid clinical detection of emotional disorders.(21) It also improved detection of comorbid disorders. A UK trial found higher levels of agreement between DAWBA and clinical diagnoses, following disclosure of DAWBA information, in relation to anxiety disorders.(20) Practitioners

For peer review only - http://bmjopen.bmj.com/site/about/guidelines.xhtml

acknowledged that the additional information could supplement the assessment and aid detection of difficulties.(10)

Hence, it might be expected that the introduction of an SDA tool following CAMHS referral receipt could enable resources to be better targeted and a timely conclusion to assessments with a diagnostic decision, increase the likelihood that an appropriate evidence-based treatment is offered, and lead to improved outcomes and better experience of care for CYP and their families. However, there is limited evidence on the utility of SDA tools for informing optimal approaches to assessment within routine clinical practice.

Aims and Objectives

The aim is to evaluate the clinical and cost effectiveness of the DAWBA SDA tool, as an adjunct to usual clinical care for CYP presenting with emotional difficulties referred to CAMHS.

Specific objectives are to:

1. Conduct an RCT to determine the effectiveness of the DAWBA as an adjunct to usual clinical care on diagnosis and treatment of emotional disorders, symptoms of emotional difficulties and comorbid disorders and associated functional impairment.

2. Undertake an internal pilot to assess recruitment and acceptability.

3. Include a qualitative component within the pilot phase to address:
   a) The feasibility of recruitment.
   b) The acceptability and usability of the interventions and procedure.
   c) How the intervention is used and could be refined for the main trial.

4. Conduct a process evaluation alongside the main trial which will:
   a) Optimise the design and delivery of the DAWBA to enhance acceptability, effectiveness and long-term uptake.
   b) Identify the barriers and facilitators to implementation of the DAWBA from the perspectives of CYP, parents, and CAMHS practitioners, managers and commissioners.

5. Estimate cost effectiveness of the use of the DAWBA versus usual care.

6. Make evidence-based recommendations for assessment procedures within CAMHS and produce practice guidelines for clinical decision-making around the referral acceptance and assessment processes.

METHODS AND ANALYSIS

Design

A multi-centre, two-arm, parallel group RCT, with embedded qualitative and health economic components.

An internal pilot period, completed in the first 9 months of recruitment, will determine feasibility of recruitment and follow-up, assessed by the independent Trial Steering Committee against pre-defined stop/go criteria. The study start date is 01-Nov-2018 and end date is 31-Oct-2022.

Setting

For peer review only

For peer review only - http://bmjopen.bmj.com/site/about/guidelines.xhtml

Recruitment will take place in at least six NHS Trusts in England, providing outpatient multidisciplinary specialist CAMHS. Sites are geographically dispersed covering urban and rural areas, thus are likely to be socio-demographically representative of CAMHS referrals in England, enabling nationally generalisable findings.

Recruitment and eligibility

Participant identification

The population is CYP presenting with emotional difficulties referred to CAMHS. Participants are identified through the usual referral pathways for the participating sites, which includes NHS and local authority managed Single/Central Point of Access referral points as well as referrals directly received and processed by CAMHS teams.

The STADIA researchers (NHS personnel, based within the CAMHS SPA/triage team to carry out research activities on behalf of the team and authorised to access referral information) at each site review the referrals received by CAMHS to identify CYP presenting with emotional difficulties, according to a standard proforma (Appendix 1. Screening form). Referrals that mentioned any current emotional difficulties will be included, regardless of the number, frequency or severity of the emotional difficulties. Potentially eligible participants are invited to consider taking part in the trial and provided with written information. The initial invitation follows standardised wording to ensure clarity and consistency of approach.

Identification of participants takes place after referral receipt, but prior to referral acceptance (Figure 1).

Consent

Prior to consent, eligibility will be confirmed (table 1) during telephone contact with the local STADIA researcher, who will also provide written and verbal information about the trial, answer questions and support the electronic consent/assent process. Participants who are eligible and provide verbal consent to participation during the call will be provided with a personal link to the online electronic Informed Consent/Assent Form (table 2, appendix 6 and 7, respectively), enabling them to provide written informed consent/assent.

Table 1. Eligibility criteria

Inclusion criteria for the CYP<br>• Aged 5 to 17 years.<br>• Referred to outpatient multidisciplinary specialist CAMHS.<br>• Presenting with emotional difficulties.<br>• If aged <16, has an eligible individual with parental responsibility (see parent/carer eligibility criteria below) willing and able to participate in the trial.<br>• If aged 16-17, has capacity to provide valid written informed consent.<br>• If aged 16-17 and participating without a parent/carer, able to complete the assessment tool in English.<br>• If aged 16-17 and participating without a parent/carer, access to internet and email or telephone.
Exclusion criteria for the CYP<br>• Emergency or urgent referral to outpatient multidisciplinary specialist CAMHS (i.e. requires an expedited assessment) according to local risk assessment procedures.<br>• Severe learning disability.<br>• Previously randomised in the STADIA trial.
Inclusion criteria for the parent/carer

For peer review only - http://bmjopen.bmj.com/site/about/guidelines.xhtml

- Individual with parental responsibility for the CYP referred to CAMHS; this will be the CYP's mother or father, legally appointed guardian or a person with a residence order concerning the CYP.
- Adequate knowledge of the CYP to be able to complete the assessment tool (i.e., known for at least 6 months).
- Has capacity to provide valid written informed consent.
- Access to internet and email or telephone.
- Able to complete the assessment tool in English.

Exclusion criteria for the parent/carer

- Local authority representatives designated to care for the CYP.

The participation and consent/assent requirements for the trial are shown in table 2.

Table 2: Consent & Participation

**Age of CYP referred to CAMHS:**	**CYP aged <11**	**CYP aged 11-15**		**CYP aged 16-17**	
**Initial contact with:**	Parent/carer			Depends on contact details provided with the CAMHS referral*	
**Consent provided by:**	Parent/carer	Parent/carer	Parent/carer	CYP AND parent/carer (optional)	CYP
**Assent provided by:**	None	CYP (optional)	None	None	None
**Participant(s):**	Parent/carer only	CYP and parent/carer dyad	Parent/carer only	CYP and parent/carer dyad	CYP only
**Primary participant:****	Parent/carer	Parent/carer	Parent/carer	CYP	CYP
**Secondary participant:**	None	CYP	Non	Parent/carer	None
**DAWBA completed by:**	Parent/carer	Parent/carer AND CYP	Parent/carer	CYP AND parent/carer	CYP
**Research questionnaires completed by:**	Parent/carer report on CYP Parent/carer self-report	Parent/carer report on CYP Parent/carer self-report CYP self-report	Parent/carer report on CYP Parent/carer self-report	CYP self-report Parent/carer report on CYP Parent/carer self-report	CYP self-report

For all CYP aged <16 the initial contact about the study will be with the parent/carer. The involvement of CYP aged 11-15 will be at the discretion of the parent/carer.
* For CYP aged 16-17 if the CYP's contact details are provided on the CAMHS referral the first contact about the study will be with the CYP who can choose to nominate a parent/carer to participate in the trial alongside them or participate alone. If the parent/carer's contact details only are available the first contact will be with the parent/carer and the parent/carer will be asked whether the CYP can also be contacted but may choose to refuse this. The parent/carer will not be able to participate in the STADIA trial without the involvement or consent of the CYP.
** The primary participant is the person who must provide consent as a minimum requirement in order for randomisation to take place. Assent (of CYP aged 11-15) and parental consent (for CYP aged 16 and 17) may also be sought but is not mandatory and therefore will not be required prior to

For peer review only

For peer review only - http://bmjopen.bmj.com/site/about/guidelines.xhtml

randomisation.

Participants are free to withdraw at any time and for any reason. Participants may withdraw from the intervention, follow-up questionnaires and/or data collection from records in any combination (e.g., participants who do not complete the intervention will continue to be followed-up, participants withdrawing from follow-up questionnaire completion may continue to consent for data collection from records). Withdrawn participants will not be replaced. Data collected prior to withdrawal will be retained and used in the analysis.

Where CYP aged 16 or 17 have consented for their own involvement they can continue to participate in the trial in the event of their parent/carer's withdrawal, however, the parent/carer involvement would not continue should the CYP withdraw consent.

Randomisation and concealment

Participants will be randomised in a 1:1 ratio to either intervention or control. Allocation will be assigned using a minimisation algorithm balancing on recruiting site, CYP age (5-10, 11-15, 16-17 years) and sex, incorporating a probabilistic element to allocation. The allocation algorithm was created by Nottingham Clinical Trials Unit (NCTU) in accordance with their Standard Operating Procedures (SOPs). Allocation is concealed using an automated web system operated by NCTU.

Randomisation is automatically generated within the online system following submission, and automated verification, of baseline data by the primary participant. Participants are presented with their allocation and further instructions on-screen with email confirmation. Instructions for DAWBA completion are included for those in the intervention arm. Email confirmation is sent to the coordinating centre and site research team.

It will not be possible to blind participants, site researchers, clinicians and some trial staff to treatment allocation, but treatment allocation data will be restricted to those trial staff who require access to facilitate trial conduct. In particular, it will not be fully possible to blind researchers conducting data collection from records. However, any possible diagnoses identified from the CAMHS records will be recorded verbatim on the data capture form and will be subject to adjudication by the trial adjudication committee (members of the Trial Management Group). The committee will be blinded to treatment allocation and participant ID.

The risk of contamination between arms is considered low. Access to the DAWBA, and provision of the DAWBA report, is only provided to participants in the intervention arm. SDA tools are not current practice in standard care and it is unlikely that control participants will be asked to complete these at the point of referral receipt. DAWBA completion occurring outside the trial for control arm participants will be collected during follow-up.

Interventions

Development and wellbeing assessment (DAWBA)

The trial intervention is the DAWBA. (24) The DAWBA has a modular structure, with only those modules relevant to emotional and comorbid disorders included; separation anxiety, specific phobia, social phobia, panic and agoraphobia, generalised anxiety, post-traumatic stress disorder (PTSD), obsessive compulsive disorder (OCD), depression, oppositional defiant disorder (ODD) and conduct disorder. Whereas, the strengths and difficulties questionnaire, bipolar disorder, and body dysmorphic disorder are not included in the STADIA-specific DAWBA report as these modules do not generate diagnostic predictions. No freetext responses are collected.

For peer review only - http://bmjopen.bmj.com/site/about/guidelines.xhtml

The DAWBA will be self-reported by participants via the secure, standalone online platform created and maintained by the DAWBA developer.(24) Access is by a unique ID number and password, assigned at the point of randomisation via a stock control system integrated into the randomisation system, ensuring accountability of DAWBA slot allocation.

The DAWBA may be completed by the parent/carer and/or CYP aged 11+, depending on the consent and participation arrangements (Table 2) DAWBA completion will be monitored and the STADIA researcher will support and encourage completion. Participants will be able to complete the DAWBA in a telephone call with the STADIA researcher if required. Participants are asked to complete all modules of the DAWBA presented to them. Should the DAWBA be only partially completed by respondents the report will be based only on fully answered modules with missing responses identified as such.

A trial-specific DAWBA report will be prepared for each participant, based on a standard, study-specific template (Appendix 2. Template DAWBA report). The algorithm-derived diagnostic predictions will be used to highlight the likelihood of a CYP meeting ICD-10 criteria for the disorders assessed; the report is based entirely on the algorithm-derived predictions and is not clinically rated. The report will be sent to participants (via post or email) and CAMHS clinicians (via upload to the clinical record), as an adjunct to usual clinical practice.

Control

CYP randomised to the control arm will receive usual care (i.e., referral review as usual). Based on standard information provided with the referral a clinical decision is made about whether the referral is accepted and, if so, a clinician conducts the initial CAMHS assessment as per usual practice in the service.

Sample size

A target sample size of 1210 participants will be recruited and randomised, with equal allocation to intervention or control.

Assuming 45% of control participants have a confirmed diagnosis within 12 months (based on unpublished data obtained from the trial sites), detection of an absolute increase of 10% with 90% power and 5% two-sided alpha, requires 544 participants per arm for analysis. Allowing for up to 10% non-collection of the primary outcome, we will randomise 1210 participants.

Measures and outcomes

Primary outcome

The primary outcome is a clinician-made diagnosis decision about the presence of an emotional disorder within 12 months of randomisation i.e. diagnosis of an emotional disorder will be coded as ‘yes’; absence or uncertainty (for example, reflecting ongoing assessment or investigation) will be coded as ‘no’. Eligible diagnoses are those that reflect ‘emotional’ or ‘internalizing’ disorders in ICD/DSM (Appendix 3. Eligible emotional disorder diagnoses). The diagnosis must be documented in the clinical record within 12 months of randomisation by a mental health services clinician in an NHS-delivered or NHS-commissioned service.

Diagnoses will be collected from clinical records using a standard proforma. Alternative possible diagnoses identified from the clinical notes will be recorded verbatim on the data capture form and will be subject to adjudication by members of the Trial Management Group (Appendix 4. Outcome Definition and Adjudication Plan).

For peer review only - http://bmjopen.bmj.com/site/about/guidelines.xhtml

Secondary outcomes

Secondary outcomes are detailed in table 3.

For peer review only - http://bmjopen.bmj.com/site/about/guidelines.xhtml
For peer review only

Table 3. Secondary outcome definitions

Outcome	Measurement	Definition
Acceptance of index referral	Collected from records	Whether the index referral (i.e., the referral made to CAMHS at the point of recruitment to the STADIA trial) was accepted or declined.<br><br>Acceptance is defined as being offered an appointment within CAMHS, whether or not the initial appointment was attended or subsequent appointments were offered/attended. Collected within 12 months of randomisation.
Acceptance of any referral within 12 months of randomisation	Collected from records	Whether the index referral or any subsequent referral to CAMHS (if made) was accepted or not .<br>Acceptance as defined above for index referral. Collected within 12 months of randomisation.
Discharge from CAMHS within 12 months	Collected from records	Whether the child/young person was discharged from CAMHS (following acceptance of the index referral) during the 12-months post-randomisation.
Re-referral to CAMHS within 12 months	Collected from records	Whether the child/young person was re-referred to CAMHS (for those whose index referral was turned down by CAMHS or those whose index referral was accepted but were subsequently discharged) during the 12-months post-randomisation.
Confirmed diagnosis decision	Collected from records	Diagnosis of an emotional disorder or confirmed absence of an emotional disorder coded as 'yes' vs. uncertainty about the presence of an emotional disorder coded as 'no'. Diagnosis as defined for primary outcome, collected within 12 months of randomisation.
Time from randomisation to diagnosis of emotional disorder	Collected from records	Date of diagnosis will be the first documented eligible diagnosis.Diagnosis as defined for primary outcome, collected within 12 months of randomisation.
Diagnoses made over the 12 month period from randomisation	Collected from records	The diagnosis must be documented in the clinical record within 12 months of randomisation by a mental health services clinician in an NHS-delivered or NHS-commissioned service. All diagnoses made within 12 months will be included. Measured using a standard proforma (pre-specified diagnoses).
Treatment offered for diagnosed emotional disorder	Collected from records	Whether treatment was offered for a diagnosed emotional disorder, as defined for primary outcome, collected within 12 months of randomisation.
Any treatment / interventions given	Collected from records	All treatments/interventions offered by CAMHS for any reason within 12 months of randomisation, whether or not there is a documented diagnosis will be included.

For peer review only - http://bmjopen.bmj.com/site/about/guidelines.xhtml

Outcome	Measurement	Definition
Time from randomisation to the decision to offer treatment for a diagnosed emotional disorder	Collected from records	Date of decision will be the first date that the decision to offer treatment for a diagnosed emotional disorder is documented in the clinical notes, collected within 12 months of randomisation.
Time from randomisation to start of first treatment for a diagnosed emotional disorder	Collected from records	Date of treatment will be the first date that any treatment offered for a diagnosed emotional disorder is started.<br><br>Treatment and diagnosed emotional disorder as defined, collected within 12 months of randomisation.
Time from randomisation to the decision to offer any treatment	Collected from records	Date of decision will be the first date that the decision to offer any treatment is documented in the clinical notes, , collected within 12 months of randomisation.
Time from randomisation to start of any treatment	Collected from records	Date of treatment will be the first date that any treatment offered is started.<br><br>Treatment as defined, collected within 12 months of randomisation.
Participant-reported diagnoses received in the 12 months post-randomisation	Participant self-report	Participants will be asked to report whether or not they received a diagnosis of the child/young person's difficulties from CAMHS in the 12 months post-randomisation and if so, what diagnosis was given and by whom.
Depression symptoms in the CYP	Mood and Feelings Questionnaire (MFQ)	Mood and Feelings Questionnaire (MFQ) (25) is a valid and reliable measure of depression in CYP.(26, 27)<br><br>33-items are answered on a 3-point scale ("not true" = 0, "somewhat true" = 1 point, "true" = 2 points).<br><br>Scores range from 0 to 66 with higher scores indicating more severe depressive symptoms. A score of 27 or higher may be indicative of depression. MFQ collected at baseline, 6 and 12 months post-randomisation.

For peer review only - http://bmjopen.bmj.com/site/about/guidelines.xhtml

Outcome	Measurement	Definition
Anxiety symptoms in the CYP	Revised CYP's Anxiety Depression Scale (RCADS)	Revised CYP's Anxiety and Depression Scale (RCADS)(28)<br>RCADS is a 47-item questionnaire that measures the reported frequency of various symptoms of anxiety and low mood. Each item is rated on a 4-point scale (never = 0, sometimes = 1, often = 2, always = 3).<br>An overall anxiety and low mood score is generated, with separate sub-scale scores for separation anxiety, social phobia, generalised anxiety, panic, obsessive compulsive disorder and major depression.<br>RCADS demonstrates good psychometric properties.(29)<br>Total anxiety and depression scores range from 0 to 141.<br><br>We will record scores for each of the 6 sub-scales. For analysis metric, we will use the total anxiety score. RCADS collected at baseline, 6 and 12 months post-randomisation.
Comorbid oppositional defiant / conduct disorder symptoms in the CYP	Strengths and Difficulties Questionnaire (SDQ)	Strengths & Difficulties Questionnaire (SDQ):(30) A 25-item emotional and behavioural screening questionnaire for CYP.<br><br>Each item is rated on a 3-point scale (not true, somewhat true, certainly true). Values of 0, 1 or 2 are assigned to each response.<br><br>SDQ comprises 5 sub-scales and an impact supplement. The impact supplement asks effect of difficulties on homelife, friendships, education and leisure activities.<br><br>SDQ has demonstrated reasonable psychometric properties.(31-34)<br>Scores on the 'conduct problems' subscale will be used in the analysis of this outcome.<br><br>Sub-scale scores range from 0 to 10. SDQ collected at baseline, 6 and 12 months post-randomisation.
Functional Impairment in the CYP	Strengths and Difficulties Questionnaire (SDQ)	Impact supplement scores will be used to determine functional impairment. Impact scores range from 0 to 10. Collected at baseline, 6 and 12-months post-randomisation.

For peer review only - http://bmjopen.bmj.com/site/about/guidelines.xhtml

Outcome	Measurement	Definition
Self-harm thoughts in the CYP	CYP self-report self-harm measure	CYP will be asked to report the frequency of thoughts of self-harm.<br>Frequency of thoughts of self-harm are rated over the last 6 months in the following categories and scored accordingly:<br>Not at all (0)<br>Once or twice (1)<br>Three or more times (2)<br>Collected at baseline, 6 months and 12-months post-randomisation.
Self-harm behaviours in the CYP	CYP self-report self-harm measure	CYP will be asked to report frequency of instances of self-harm behaviour.<br>Frequency of self-harm behaviour are rated over the last 6 months in the following categories and scored accordingly:<br>Not at all (0)<br>Once (1)<br>Two or more times (2)<br>Collected at baseline, 6 months and 12-months post-randomisation.
Depression symptoms in the parent/carer	Patient Health Questionnaire (PHQ-9)	PHQ-9:(35) PHQ-9 is frequently used as a screening tool for depression in general populations. Each of the nine DSM-IV depression criteria are scored as "0" (not at all) to "3" (nearly every day) depending on the frequency with which they were experienced over the last 2 weeks.<br>Total scores range from 0 to 27 with higher scores indicating increased severity of depression, collected at baseline, 6 and 12-months post-randomisation.
Anxiety symptoms in the parent/carer	Generalised Anxiety Disorder Assessment (GAD-7)	GAD-7:(36)<br>GAD-7 is a measure of the severity of anxiety in general populations. 7 items are rated according to the frequency with which they have been experienced over the past 2 weeks (0 = 'not at all', 1 = 'several days', 2 = 'more than half the days', and 3 = 'nearly every day').<br>Total scores range from 0 to 21 with higher scores indicating more severe anxiety. Collected at baseline, 6 and 12-months post-randomisation.
Time off education, employment or training because of emotional difficulties for the CYP	Resource use questionnaire	Days missed from education, employment or training (as applicable) for the CYP due to emotional difficulties. Collected at baseline, 6 and 12-months post-randomisation.

For peer review only - http://bmjopen.bmj.com/site/about/guidelines.xhtml

Outcome	Measurement	Definition
Health economic outcome measures		
Health related quality of life in the CYP	Child Health Utility 9D (CHU9D) and EuroQol Quality of Life Questionnaire 5 Domains for Young People (EQ-5D-Y)	CHU9D (37) consists of nine individual items with five levels of response per question (scored 1-5), that assess the CYP functioning “today”. The following domains are included; worry, sadness, pain, tiredness, annoyance, school, sleep, daily routine and activities.<br><br>EuroQol-5D youth descriptive system (38) comprises 5 domains; mobility, looking after myself, doing usual activities, having pain or discomfort and feeling worried, sad or unhappy, values of 1, 2 or 3 are assigned to each response. The EuroQol Visual Analogue Scale (EQ-VAS) asks recipients to self-assess their health state ‘today’ from 0 (worst imaginable health) to 100 (best imaginable health), representing individual preferences.<br><br>These measures will be self-reported by CYP aged 11+, with proxy versions also completed by the parent/carer for CYP <16.<br><br>Both collected at baseline, 6 and 12-months post-randomisation.
Health-related quality of life in the parent/carer	EuroQol Quality of Life Questionnaire 5 Domains, 5 Levels (EQ-5D-5L)	The EuroQol 5-dimension multi attribute utility instrument (39)comprises 5 domains; mobility, self-care, usual activities, pain/discomfort and anxiety/depression. Each domain is scored between 1 and 5. This descriptive profile, in combination with a valuation set, produces a single index for health status representing societal preferences. The index score ranges from -0.59 to 1, with 0 representing death, 1 of-perfect health, and <0 of health states worse than death. The EQ-VAS is again included within the EQ-5D instrument Collected at baseline, 6 and 12-months post-randomisation.

For peer review only

For peer review only - http://bmjopen.bmj.com/site/about/guidelines.xhtml

Health economic measures

Health related quality of life (HRQoL) outcome measures are detailed in table 3.

Resource Use

Data will be collected on health care, education, and social care resource use for both the CYP and parents/carers, using a purposely designed resource use collection tool. The questionnaire was developed by health economists, in tandem with feedback from PPI representatives, addressing primary, secondary, and social care costs, alongside the broader patient-borne costs. These data will be attributable to the emotional difficulties of the young person and be self-reported by the parent/carer with supplementary information obtained from CYP aged 16 and 17. Administrative records of treatments/interventions offered by CAMHS during the trial period may be considered as a supplementary source of data.

Socio-demographic data

The following socio-demographic data will be collected primarily from the participant-reported questionnaires; age at randomisation, sex, gender, ethnicity, paid employment, and, derived from the postcode of the child's primary residence, the index of Multiple Deprivation score.

Data collection

Data will be collected through participant reported questionnaires (parent/carer and CYP self-report aged 11+) and from clinical records. Participant reported outcomes will be collected at baseline and 6- and 12-months post-randomisation (Appendix 5. Summary of assessments). Questionnaires are intended to be completed online by participants in the first instance - to maximise rates of completion and retention there will be an option for telephone completion, should participants have difficulty accessing or completing the questionnaires online.

Outcomes collected from records will be reported for the 12-month period following randomisation.

Data management and analysis

Data management

Arrangements for data handling are specified in the Data Management Plan (DMP). Central and on-site monitoring will be carried out as required following a risk assessment and as documented in the monitoring plan. Monitoring activities will be carried out by the coordinating centre on behalf of the trial sponsor.

Data will be held on servers located within The University of Nottingham data centres. Security is both physical (secure limited access) and electronic (behind firewalls, access via user accounts). Personal data recorded on all documents will be regarded as strictly confidential and handled and stored in accordance with the Data Protection Act 2018.

Statistical analysis

The primary approach to between-group comparative analyses will be by modified intention-to-treat (i.e. including all participants who have been randomised and without imputation of missing outcome data).

The primary comparative analysis will employ a generalised linear mixed model to compare the proportions in each group with a clinician-made diagnosis decision within 12 months of randomisation, adjusted for minimisation variables. The comparison will be presented as both an absolute (risk difference) and relative (risk ratio) effect, along with 95% confidence intervals.

For peer review only - http://bmjopen.bmj.com/site/about/guidelines.xhtml

Secondary outcomes will be analysed using appropriate mixed effect regression models dependent on data type and will adjust for factors used in the minimisation and baseline value of the outcome where measured. For outcomes measured at multiple time points, these will be analysed using a mixed model with a treatment by time interaction to obtain estimates of treatment effect at each follow-up time.

Appropriate interaction terms will be included in the primary regression analyses in order to conduct subgroup analyses according to sex and age of the CYP.

Statistical analysis will be conducted using Stata v17.0 (or later).

Health economic analysis

In accordance with NICE guidance, primary analysis will take an NHS and personal social services perspective. Unit costs will be attached to participant reports of health care resource use or recorded treatments/interventions offered by CAMHS. The cost of the DAWBA itself will be distributed at the participant-level across the intervention arm of the trial. Sensitivity analyses will take a wider perspective to capture the broader societal costs inclusive of out-of-pocket expenses and productivity losses. Indices of HRQoL for the EQ-5D, EQ-5D-Y, and CHU9D will be derived using relevant population tariffs, and quality adjusted life years estimated using area under the curve (AUC).

The economic evaluation will take an incremental approach between the two groups using an intention-to-treat (ITT) population (irrespective of treatment received) and a 12-month time horizon. The outcome for the primary cost utility analysis will be the joint young person and parent/carer QALYs. The outcome for the secondary cost effectiveness analysis will be confirmed diagnosis decisions. Outcomes will be paired with their respective direct-to-NHS costs, bootstrapped, and scattered on the cost effectiveness plane to characterise the uncertainty in incremental estimates. Using the net monetary benefit framework,(40) Cost Effectiveness Acceptability Curves (CEACs) will be constructed to show the non-parametric probability the intervention is a cost effective option, compared to usual care, across a range of willingness to pay thresholds per QALY, and within the secondary analysis per confirmed diagnosis decision. While the receipt of any diagnosis of emotional difficulties in young people would likely lead to large divergences in lifecourse outcomes, the heterogeneity of conditions considered for diagnosis (Appendix 3) renders CUA modelling across the lifecourse infeasible. Secondary analysis is expected to be fully captured within the 12-month time horizon.

A full statistical analysis plan (SAP) and health economics analysis plan (HEAP) will be developed and agreed prior to database lock and un-blinding of the analysing statistician and health economist.

Embedded qualitative study

During the internal pilot, semi-structured interviews are undertaken with a sample of participants who consented to be invited to participate in qualitative interviews. Researchers, clinicians, service managers and commissioners are identified by site leads. The proposed sample size is 25 participants (parent/carer and CYP aged 16-17), 25 staff and 15 service managers and commissioners. Interviews address: a) the feasibility of recruitment; b) the acceptability and usability of the interventions and procedure; c) how the intervention is used and how this deployment could be refined for the main trial. Interviews are conducted by the qualitative researcher (KN) in person, or by phone or video call based on participant preferences and pandemic restrictions.

A process evaluation, conducted during the main trial phase, will aim to identify the barriers and facilitators to implementation of the intervention. Semi-structured interviews will be conducted with

For peer review only - http://bmjopen.bmj.com/site/about/guidelines.xhtml

a further sample of participants and clinicians to explore the perceived functioning of the intervention, the organisation of the service and reflective experiences on outcomes.

Qualitative interview data will be recorded and encrypted on a password protected Dictaphone and transferred securely to medical transcription company Dict8 for transcription. Transcriptions will be anonymised. Audio files will be destroyed after transcripts have been checked. Anonymised transcriptions will be analysed and stored on password protected computers and the secure University of Nottingham server.

Qualitative analysis

All qualitative interview data will be initially analysed by the qualitative researcher (KN) using interpretative thematic approaches to coding, and adopt the framework method,(41) with input from the qualitative lead (LT), Chief Investigator (KSa) and PPI leads (CE & AL). NVIVO 12 will be used to manage the qualitative data.

Patient and public involvement

Prior to submission, the proposal was informed by consultations with a person with lived parent/carer experience of CAMHS, including contribution to and review of the proposal, recruitment strategy, participant trial experience and consideration of burden of the intervention, and establishing a PPI workstream.

Following award, the PPI Co-I team recruited two representatives naïve of the study design to provide independent review of the trial via their membership of the Trial Steering Committee (TSC). Both TSC members are persons with lived parent/carer experience of CAMHS.

During study set up, PPI Co-I expertise was utilised to support researcher recruitment via the design and deployment of role plays within interviews.(42) This was to gain insight into candidates' capabilities when dealing with sensitive and challenging participant scenarios. Additionally, they contributed to design of researcher training materials, to support standardised approaches across trial sites. Iterative and creative design PPI activities were integral in the development of the STADIA trial logo and branding to ensure accessibility and acceptability to CYP and parents.

Since study commencement participatory design approaches have seen PPI co-design of the resource use questionnaire, qualitative interviews and the protocol for a Study Within A Trial (SWAT) to support participant engagement with follow-up. Additionally, collaborative working between the PPI and Qualitative workstreams has enabled examination of the qualitative themes using principles of the Framework Method(41) for independent verification of those themes.

Two PPI advisory panels have been established, meeting on average every 3 months since month 9 of the study. "STADIA PPI Panel" has 8 adult members, with lived parent/carer experience of CAMHS. "STADIA Labs" has 6 CYP members, aged 15 to 19 at inception, with lived experience of CAMHS. These groups have been involved in many traditional activities such as review of PIS and consent forms, consultation on language and content for participant reminder text messages. PPI co-production activities are also seeing the development of age appropriate study newsletters and the design of STADIA information videos including decision making about video concept, audience, message, aesthetic and content. PPI group members are provided with supplementary training about PPI practices and involvement opportunities. Due to the Covid-19 pandemic, PPI meetings have had to move online and so the PPI team are investing in knowledge transfer and upskilling PPI representatives in different ways of working and collaborating online.

For peer review only - http://bmjopen.bmj.com/site/about/guidelines.xhtml

There are a range of planned flexible opportunities for participating in project feedback and dissemination activities including co-facilitating and presenting at the interactive dissemination workshop / consensus meeting, publication authorship as peer researcher and presenting at conferences to showcase the project findings.

ETHICS AND DISSEMINATION

Ethics

The study was reviewed and received favourable opinion from the South Birmingham Research Ethics Committee (Ref. 19/WM/0133) on 12 June 2019; subsequent amendments have been approved. The current, approved protocol is version 4.0 dated 03 February 2021.

Safety

The trial intervention is conceptually similar to usual clinical practice (i.e., CYP referred to CAMHS may be sent questionnaires about their difficulties), therefore the risks of the trial are considered comparable. The DAWBA is widely used in research for data collection therefore, although utilised as an intervention in the STADIA trial, the risks may be regarded as similar to those of an observational/questionnaire study. Data to inform safety oversight will therefore be collected during routine follow-up, from existing outcome measures. There is no separate adverse event or serious adverse event reporting.

The number of participants meeting pre-defined safety outcomes will be reported on an ongoing basis to the Trial Management Group (TMG) and TSC. Data will be presented by arms to the Data Monitoring Committee (DMC).

Trial oversight

Nottinghamshire Healthcare NHS Foundation Trust will undertake role of Sponsor as defined by the UK Policy Framework for Health and Social Care Research.(43) Delegated responsibilities will be assigned to the Chief Investigator, participating NHS Trusts and the trial coordinating centre, Nottingham Clinical Trials Unit (NCTU).

The full co-applicant team and NCTU staff responsible for the day-to-day management of the trial will form the TMG, responsible for monitoring recruitment and retention rates and implementing strategies to ensure targets are met. Independent Trial Steering and Data Monitoring Committees will operate in accordance with trial-specific Charters.

Dissemination

Results of this trial will be reported to the funder and published in full in the HTA Journal series and also submitted for publication in a peer reviewed journal.

Data Sharing

Anonymised trial data may be shared with researchers external to the trial research team in accordance with the NCTU's data sharing procedure.

Figures

Figure 1: Participant flow

Authors' contributions

FD, LW, AB, BD, CE, JG, MJ, AL, TM, AM, SR, KSp, LT, EB, JL, KN, CP, KSt and KSa made substantial contributions to conception and design or acquisition of data; took part in drafting the article or

For peer review only

For peer review only - http://bmjopen.bmj.com/site/about/guidelines.xhtml

revising it critically for important intellectual content; agreed to submit to the current journal; gave final approval of the version to be published; and agree to be accountable for all aspects of the work. KSa is guarantor for the paper. FD and LW contributed equally to this paper.

Funding statement

This study was funded as a result of a commissioned call by the National Institute for Health Research (NIHR) Health Technology Assessment programme (Grant Reference Number 16/96/09). The views expressed are those of the authors and not necessarily those of the NIHR or the Department of Health and Social Care.

The funder will have no role in the collection, management, analysis, and interpretation of data; writing of the report; and the decision to submit the report for publication.

Competing interests

The authors declare no competing interests.

Acknowledgements

We would like to acknowledge and thank all the parent/carers and young people participating in the trial and the research sites involved in recruiting participants and data collection. The authors would also like to thank the wider STADIA team for their input, including the PPI Advisory Panels, members of the independent Trial Steering and Data Monitoring Committees, and the Nottingham Clinical Trials Unit, who are the trial coordinating centre.

Finally, thanks to the trial sponsor, Nottinghamshire Healthcare NHS Foundation Trust (researchsponsor@nottshc.nhs.uk).

For peer review only - http://bmjopen.bmj.com/site/about/guidelines.xhtml

REFERENCES

1. Sadler K, Vizard T, Ford T, Marcheselli F, Pearce N, Mandalia D, et al. Mental Health of Children and Young People in England, 2017: NHS Digital; 2018 [Available from: https://digital.nhs.uk/data-and-information/publications/statistical/mental-health-of-children-and-young-people-in-england/2017/2017.
2. Goodyer IM RS, Barrett B, Byford S, Dubicka B, Hill J, Holland F, Kelvin R, Midgley N, Roberts C, Senior R, Target M, Widmer B, Wilkinson P, Fonagy P. Cognitive behavioural therapy and short-term psychoanalytic psychotherapy versus brief psychosocial intervention in adolescents with unipolar major depression (IMPACT): a multicentre, pragmatic, observer-blind, randomised controlled trial. Health Technology Assessment. 2017;21(12).
3. Simmons M, Wilkinson P, Dubicka B. Measurement Issues: Depression measures in children and adolescents. Child and Adolescent Mental Health. 2015;20(4):230-41.
4. Colman IW, MEJ. Croudace, TJ. Jones, PB. Forty-Year Psychiatric Outcomes Following Assessment for Internalizing Disorder in Adolescence. American Journal of Psychiatry. 2007;164(1):126-33.
5. Orchard F, Pass L, Marshall T, Reynolds S. Clinical characteristics of adolescents referred for treatment of depressive disorders. Child and Adolescent Mental Health. 2017;22(2):61-8.
6. Frith E. Access and waiting times in children and young people's mental health services.: Education Policy Institute; 2017 [Available from: https://epi.org.uk/wp-content/uploads/2018/01/EPI_Access-and-waiting-times_.pdf.
7. Michelson D, Rock S, Holliday S, Murphy E, Myers G, Tilki S, et al. Improving psychiatric diagnosis in multidisciplinary child and adolescent mental health services. The Psychiatrist. 2011;35:454-9.
8. O'Connor C, Downs J, McNicholas F, Cross L, Shetty H. Documenting diagnosis in child and adolescent mental healthcare: A content analysis of diagnostic statements in a psychiatric case register. Children and Youth Services Review. 2020;113:104948.
9. Allsopp K, Kinderman P. The use of diagnoses in mental health service eligibility and exclusion criteria. Journal of mental health (Abingdon, England). 2021;30(1):97-103.
10. Martin A-M, Fishman R, Baxter L, Ford T. Practitioners' attitudes towards the use of standardized diagnostic assessment in routine practice: A qualitative study in two Child and Adolescent Mental Health Services. Clinical Child Psychology and Psychiatry. 2011;16(3):407-20.
11. Craddock N, Antebi D, Attenburrow M-J, Bailey A, Carson A, Cowen P, et al. Wake-up call for British psychiatry. British Journal of Psychiatry. 2008;193(1):6-9.
12. National Institute for Health and Clinical Excellence. Depression in Children and Young People: Quality Standard London: National Institute for Health and Clinical Excellence; 2013 [Available from: https://www.nice.org.uk/guidance/cg28.
13. Fitzpatrick C AN-N, Kehoe A, Devlin N, Glackin S, Power L, et al. Do we miss depressive disorders and suicidal behaviours in clinical practice. Clinical Child Psychology and Psychiatry. 2012;17:449-58.
14. National Institute for Health and Clinical Excellence. Depression in Children and Young People: Identification and Management in Primary, Community and Secondary Care – Update London: National Institute for Health and Clinical Excellence,; 2015 [Available from: https://www.nice.org.uk/guidance/cg28.
15. Last A, Henley, W., Norman, S., Goodman, R., Ford, T. Innovations in Practice: Feasibility of the development and well-being assessment as an adjunct to clinical assessment in child and adolescent mental health services. . Child and Adolescent Mental Health. 2014;19:142-6.
16. Reeves K, Charter E, Ford T. Measurement Issues: Is standardised diagnostic assessment feasible as an adjunct to clinical practice? A systematic review. Child and Adolescent Mental Health. 2016;21(1):51-63.

For peer review only - http://bmjopen.bmj.com/site/about/guidelines.xhtml

17. Goodman R, Ford T, Richards H, Gatward R, Meltzer H. The Development and Well-Being Assessment: Description and Initial Validation of an Integrated Assessment of Child and Adolescent Psychopathology. Journal of Child Psychology and Psychiatry. 2000;41(5):645-55.
18. Hall CL, Davies EB, Andrén P, Murphy T, Bennett S, Brown BJ, et al. Investigating a therapist-guided, parent-assisted remote digital behavioural intervention for tics in children and adolescents—'Online Remote Behavioural Intervention for Tics' (ORBIT) trial: protocol of an internal pilot study and single-blind randomised controlled trial. BMJ Open. 2019;9(1):e027583.
19. Pickles A, Le Couteur A, Leadbitter K, Salomone E, Cole-Fletcher R, Tobin H, et al. Parent-mediated social communication therapy for young children with autism (PACT): long-term follow-up of a randomised controlled trial. The Lancet. 2016;388(10059):2501-9.
20. Ford T, Last A, Henley W, Norman S, Guglani S, Kelesidi K, et al. Can standardized diagnostic assessment be a useful adjunct to clinical assessment in child mental health services? A randomized controlled trial of disclosure of the Development and Well-Being Assessment to practitioners. Social Psychiatry and Psychiatric Epidemiology. 2013;48(4):583-93.
21. Aebi M, Kuhn C, Metzke CW, Stringaris A, Goodman R, Steinhausen H-C. The use of the development and well-being assessment (DAWBA) in clinical practice: a randomized trial. European child & adolescent psychiatry. 2012;21(10):559-67.
22. Meltzer H, Gatward R, Goodman R, Ford T. The mental health of children and adolescents in Great Britain London: Office for National Statistics; 2000 [Available from: http://webarchive.nationalarchives.gov.uk/20160105160709/http://www.ons.gov.uk/ons/rel/psychiatric-morbidity/the-mental-health-of-children-and-adolescents-in-great-britain/1999-survey/mental-health-of-children-and-adolescents-in-great-britain.pdf.
23. Green H, McGinnity A, Meltzer H, Ford T, Goodman R. Mental health of children and young people in Great Britain, 2004 Basingstoke, Hamshire: Palgrave Macmillan; 2005 [Available from: https://webarchive.nationalarchives.gov.uk/20180307185414/http://digital.nhs.uk/catalogue/PUB06116.
24. Youth in Mind. Information for researchers and clinicians about the Development and Well-Being Assessment: Youth in Mind; 2012 [Available from: http://www.dawba.info/.
25. Angold A, Costello EJ, Messer SC, Pickles A. Development of a short questionnaire for use in epidemiological studies of depression in children and adolescents. International Journal of Methods in Psychiatric Research. 1995;5(4):237-49.
26. Wood A, Kroll L, Moore A, Harrington R. Properties of the mood and feelings questionnaire in adolescent psychiatric outpatients: a research note. 1995;36:327-34.
27. Daviss WB, Birmaher B, Melhem NA, Axelson DA, Michaels SM, Brent DA. Criterion validity of the Mood and Feelings Questionnaire for depressive episodes in clinic and non-clinic subjects. 2006;47:927-34.
28. Chorpita BF, Yim L, Moffitt C, Umemoto LA, Francis SE. Assessment of symptoms of DSM-IV anxiety and depression in children: a revised child anxiety and depression scale. Behaviour Research and Therapy. 2000;38(8):835-55.
29. Chorpita BF, Moffitt CE, Gray J. Psychometric properties of the Revised Child Anxiety and Depression Scale in a clinical sample. Behaviour Research and Therapy. 2005;43(3):309-22.
30. Goodman R. The Extended Version of the Strengths and Difficulties Questionnaire as a Guide to Child Psychiatric Caseness and Consequent Burden. Journal of Child Psychology and Psychiatry. 1999;40(5):791-9.
31. Yao S, Zhang C, Zhu X, Jing X, McWhinnie CM, Abela JRZ. Measuring Adolescent Psychopathology: Psychometric Properties of the Self-Report Strengths and Difficulties Questionnaire in a Sample of Chinese Adolescents. Journal of Adolescent Health. 2009;45(1):55-62.
32. Goodman R. Psychometric Properties of the Strengths and Difficulties Questionnaire. Journal of the American Academy of Child & Adolescent Psychiatry. 2001;40(11):1337-45.
33. Muris P, Meesters C, van den Berg F. The Strengths and Difficulties Questionnaire (SDQ). European child & adolescent psychiatry. 2003;12(1):1-8.

For peer review only - http://bmjopen.bmj.com/site/about/guidelines.xhtml

34. Lundh LG, Wangby-Lundh, M., & Bjarehed, J. . Self reported emotional and behavioral problems in Swedish 14 to 15-year-old adolescents: A study with the self-report version of the Strengths and Difficulties Questionnaire. Scandinavian Journal of Psychology. 2008;49:523-32.
35. Kroenke K, Spitzer RL, Williams JB. The PHQ-9: validity of a brief depression severity measure. Journal of general internal medicine. 2001;16(9):606-13.
36. Spitzer RL, Kroenke K, Williams JW, Löwe B. A brief measure for assessing generalized anxiety disorder: The gad-7. Archives of Internal Medicine. 2006;166(10):1092-7.
37. Stevens K. Developing a descriptive system for a new preference-based measure of health-related quality of life for children. Quality of Life Research. 2009;18(8):1105-13.
38. Wille N, Badia X, Bonsel G, Burström K, Cavrini G, Devlin N, et al. Development of the EQ-5D-Y: a child-friendly version of the EQ-5D. Quality of life research : an international journal of quality of life aspects of treatment, care and rehabilitation. 2010;19(6):875-86.
39. Herdman M, Gudex C, Lloyd A, Janssen M, Kind P, Parkin D, et al. Development and preliminary testing of the new five-level version of EQ-5D (EQ-5D-5L). Quality of Life Research. 2011;20(10):1727-36.
40. Hoch JS, Briggs AH, Willan AR. Something old, something new, something borrowed, something blue: a framework for the marriage of health econometrics and cost-effectiveness analysis. Health Economics. 2002;11(5):415-30.
41. Gale NK, Heath G, Cameron E, Rashid S, Redwood S. Using the framework method for the analysis of qualitative data in multi-disciplinary health research. BMC Medical Research Methodology. 2013;13(1):117.
42. Furniss D, Lang AR, Ewart C. Enhancing Patient Ergonomics with Patient and Public Involvement in Research Projects. . In: Holden RJ, Valdez RS, editors. The Patient Factor: A Handbook on Patient Ergonomics: CRC Press; 2001.
43. Health Research Authority. UK Policy Framework for Health and Social Care Research: Health Research Authority; 2017 [Available from: https://www.hra.nhs.uk/planning-and-improving-research/policies-standards-legislation/uk-policy-framework-health-social-care-research/

For peer review only

For peer review only - http://bmjopen.bmj.com/site/about/guidelines.xhtml

Figure 1: Participant flow

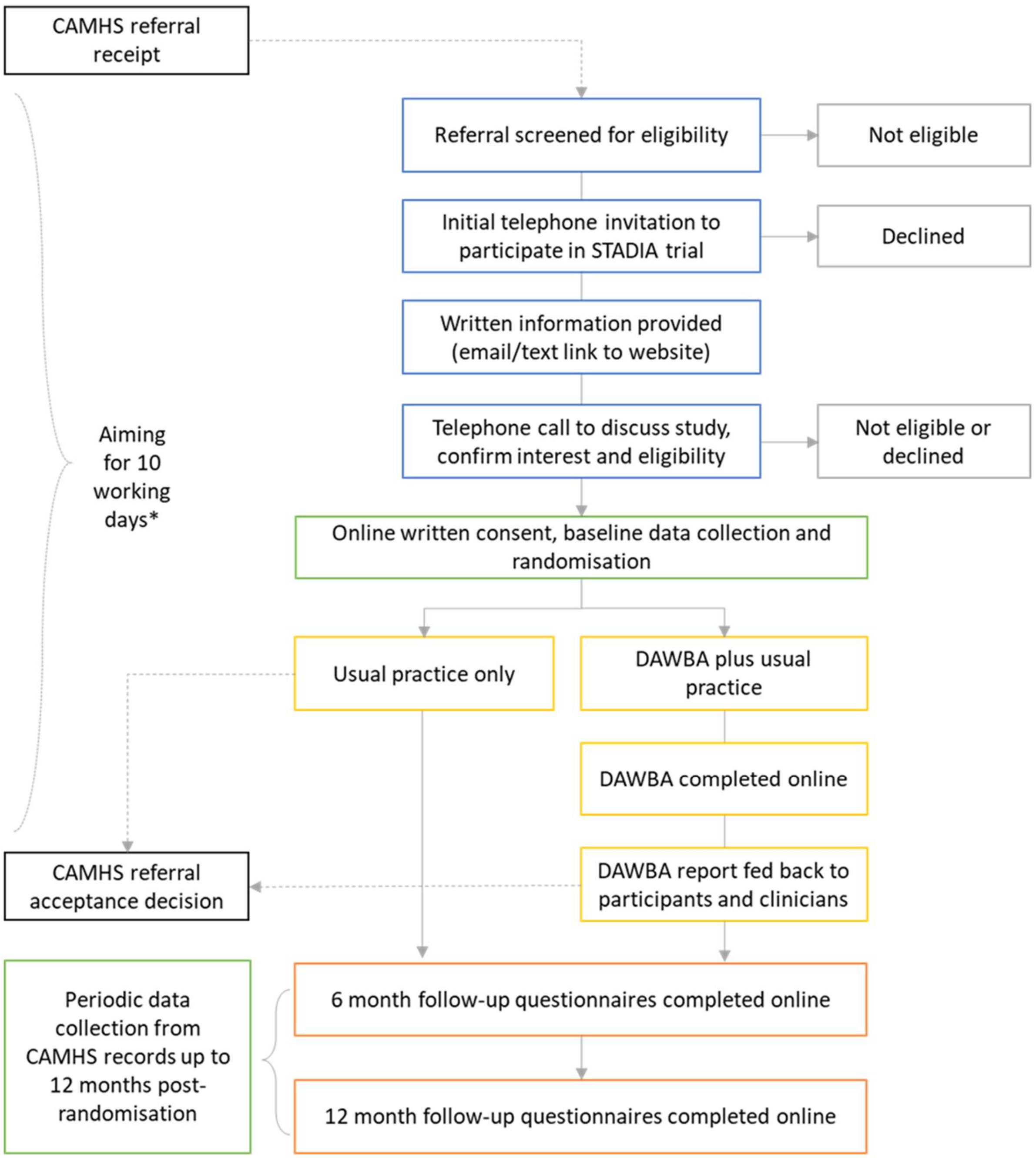

** For sites where the waiting time for the CAMHS acceptance decision usually exceeds 10 working days from referral receipt, recruitment activities may start and/or continue beyond 10 working days from referral receipt, providing the intervention period can be completed prior to the CAMHS referral decision.*

For peer review only

STAndardised DIagnostic Assessment for children and adolescents with emotional difficulties (STADIA): a multi-centre randomised controlled trial

SCREENING

**Site Number:**	
**Screening Number:**	
**Sponsor:**	**Nottinghamshire Healthcare NHS Foundation Trust**
**CRF Version:**	**Final v1.1 – 30 April 2019**

peer review only

Site Number:

Screening Number:

REFERRAL SCREENING	
Complete for all referrals screened for eligibility:	
NHS Number<br>*Local use only*	____________________
Trust Number<br>*Local use only*	____________________
Date of referral receipt (dd-mmm-yyyy)	- -
Date of screening (dd-mmm-yyyy)	- -
Young person's sex	Male ☐<br>Female ☐
Young person's age<br>*If <5 or >17 do not proceed*	
Has the young person been previously enrolled and randomised in the STADIA trial?<br>*If yes, do not proceed*	Yes ☐<br>No ☐
Does the referral mention any of the following Covid-19 related words/phrases?<br>*Tick all that apply.*<br>Covid-19 / Covid<br>Coronavirus<br>Lockdown<br>School closure / exams cancelled	 <br>☐<br>☐<br>☐<br>☐
Does the referral mention emotional difficulties*?<br>*If no, do not proceed*	Yes ☐<br>No ☐
Is this an emergency or urgent referral (according to local CAMHS triage / SPA team risk assessment)?<br>*If yes, do not proceed*	Yes ☐<br>No ☐
Does the young person have severe learning disability (e.g., the referral mentions this or that they attend a special school for children with severe learning difficulties)?<br>*If 'yes' do not proceed*<br>*If not known, confirm during telephone eligibility check at enrolment*	Yes ☐<br>No ☐<br>Not known ☐

For peer review only - http://bmjopen.bmj.com/site/about/guidelines.xhtml

Site Number: | | |

Screening Number: | | | |

REFERRAL SCREENING	
If the young person is <16:<br>Does the referral information include contact details for a named parent/carer?<br>*If 'no' await parent/carer contact details before proceeding*	Yes ☐<br>No ☐<br>N/A ☐
If the young person is <16:<br>Is the named parent/carer a local authority representative designated to care for the child/young person?<br>*If 'yes' do not proceed*<br>*If not known, confirm during telephone eligibility check at enrolment*	Yes ☐<br>No ☐<br>Not known ☐<br>N/A ☐
If the young person is aged 16 or 17:<br>Whose contact details are given on the referral form?<br>*If young person contact details are provided, they should be contacted in the first instance*	Young person ☐<br>Parent/carer ☐<br>Both ☐<br>N/A ☐

EMOTIONAL DIFFICULTIES	
**Emotional difficulties may be indicated by the use of any of the following key words or phrases.*<br>*Tick all that apply. If 'other' record details and seek advice from the PI or NCTU before proceeding.*	
None	☐
Agitated / agitation	☐
Anger	☐
Anxiety / anxious / generalised anxiety	☐
Avoids things/people/places	☐
Can't leave the house	☐
Completing rituals / asking parents to carry out rituals	☐
Compulsions	☐
Depressed / depression / low / low mood / sad	☐
Difficulties sleeping	☐
Distress	☐
Fears and worries / fears relating to safety (germs, fire)	☐
Feeling low	☐
Feels flat / empty / blank	☐

For peer review only

For peer review only - http://bmjopen.bmj.com/site/about/guidelines.xhtml

Site Number: ☐☐

Screening Number: ☐☐☐

EMOTIONAL DIFFICULTIES	
**Emotional difficulties may be indicated by the use of any of the following key words or phrases. Tick all that apply. If 'other' record details and seek advice from the PI or NCTU before proceeding.*	
Feels hopeless	☐
Feels worthless / stupid	☐
Flashbacks	☐
Hypervigilance	☐
Irritable	☐
Low motivation	☐
Low self-esteem / Hates self	☐
Mood swings / moody	☐
Negative thoughts	☐
Nightmares (if trauma also present)	☐
No (or loss of) energy	☐
No (or loss of) interest in things / gave up… / lack of wanting to do things	☐
Not going to school / unable to go to school	☐
Not sleeping / poor sleep	☐
Obsessions	☐
OCD	☐
Phobia	☐
Panic / panic attacks	☐
PTSD	☐
Self-harm / DSH / Cutting	☐
Suicidal	☐
Suicidal thoughts / thoughts of ending life / thinks about killing self	☐
Tearful	☐
Thoughts of death	☐

For peer review only - http://bmjopen.bmj.com/site/about/guidelines.xhtml

Site Number:

Screening Number:

EMOTIONAL DIFFICULTIES	
**Emotional difficulties may be indicated by the use of any of the following key words or phrases. Tick all that apply. If 'other' record details and seek advice from the PI or NCTU before proceeding.*	
Tiredness / fatigue	☐
Touching objects	☐
Trauma	☐
Weepy	☐
Withdrawal / withdrawn	☐
Worried / worrying (incl. worries/concerns about their appearance	☐
Other (please specify)	☐

FOR ALL REFERRALS SCREENED, ENTER SUMMARY DATA ON THE SCREENING & ENROLMENT LOG.

IF THE YOUNG PERSON APPEARS TO BE ELIGIBLE PROCEED TO THE INVITATION TELEPHONE CALL (CALL 1) AND ENTER DETAILS ON THE TRIAL DATABASE.

SIGN-OFF STATEMENT	
Completed by the researcher conducting the referral screening.	
To the best of my knowledge, I confirm that I have made every reasonable effort to ensure that ALL of the data in this Case Record Form is a true, accurate and complete report.	
Print Name	
Signature	
Date	- -

For peer review only - http://bmjopen.bmj.com/site/about/guidelines.xhtml

DAWBA Report

The DAWBA collects information about a range of common emotional and behavioural difficulties, and uses this information to produce a report to highlight the level of difficulties.

How to understand the ratings

These ratings compare your responses with the responses from large numbers of other parents and young people across the UK. Many parents and young people find this sort of comparison helpful, but it is just a guide and not the same as a face-to-face assessment with a specialist.

To make it easier to read, we have grouped the ratings into four categories. Each category is different. This shows how your [child's] *(delete as appropriate)* difficulties compare with other children / young people:

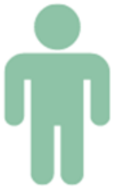

Close to average

In the general population most children/ young people (roughly 80 out of 100) are in the "close to average" category.

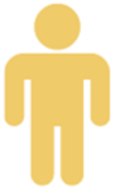

Slightly raised

If the ratings are in the "slightly raised" category this means the difficulties are slightly higher than average. Roughly 10 out of 100 children / young people are in this category.

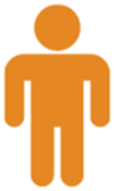

High

Around 5 in 100 children / young people score in the "high" category. This means that the difficulties are more severe than average.

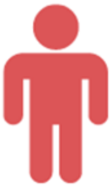

Very high

Around 5 in 100 children score in the "very high" category. This means that the difficulties appear to be more severe than we find in 95 out of every 100 children / young people.

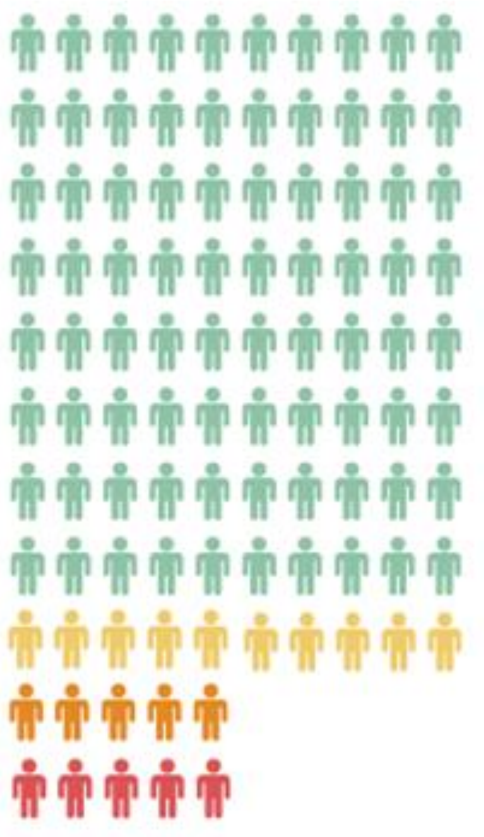

The rating is only a rough guide. As high ratings can be a "false alarm", please use your own judgement. Not all difficulties need treating. Some difficulties get better by themselves, particularly if they are mild or if they have only been there for a short time.

Most strengths and difficulties lie on a scale. There will be children / young people at each end of the scale but most children / young people will fall somewhere in between.

For peer review only - http://bmjopen.bmj.com/site/about/guidelines.xhtml

Your [child's] *(delete as appropriate)* **ratings:**

- **Close to average / Slightly raised / High / Very high** for worrying a lot about different things (general fears and worries)
- **Close to average / Slightly raised / High / Very high** for worries about separation from key "attachment figures" such as parents (separation anxiety)
- **Close to average / Slightly raised / High / Very high** for specific fears (specific phobia)
- **Close to average / Slightly raised / High / Very high** for social fears (social anxiety)
- **Close to average / Slightly raised / High / Very high** for panic attacks
- **Close to average / Slightly raised / High / Very high** for fears of crowds, public places, open spaces etc (agoraphobia)
- **Close to average / Slightly raised / High / Very high** for stress linked to particularly frightening events (post-traumatic stress)
- **Close to average / Slightly raised / High / Very high** for obsessions or compulsions
- **Close to average / Slightly raised / High / Very high** for depression or loss of interest
- **Close to average / Slightly raised / High / Very high** for disruptive and uncooperative behaviours (troublesome behaviour)
- **Close to average / Slightly raised / High / Very high** for antisocial or aggressive behaviours that can get people into serious trouble (troublesome behaviour)

For peer review only

For peer review only - http://bmjopen.bmj.com/site/about/guidelines.xhtml

Appendix 3. Eligible emotional disorder diagnoses

Anxiety disorder
Separation anxiety disorder
Specific phobia (any)
Social phobia or Social anxiety disorder
Agoraphobia
Panic disorder (DSM5 additionally has Panic Attack with a specifier)
Phobic anxiety disorder (unspecified)
Selective mutism
Generalized anxiety disorder
Obsessive-compulsive and related disorders
Body dysmorphic disorder
Acute stress reaction
Acute Stress Disorder
Post-traumatic stress disorder
Adjustment Disorder
Other anxiety disorder
Mixed anxiety and depressive disorder
Depression
Depressive episode (any / mild / moderate / severe)
Depressive disorder
Recurrent depressive disorder (any / mild / moderate / severe)
Major Depressive disorder
Persistent Depressive disorder
Other depressive episode
Persistent mood (affective) disorder (including cyclothymic disorder / dysthymic disorder)
Other / Unspecified mood (affective) disorder
Bipolar disorder
Bipolar affective disorder
Manic episode
Childhood emotional disorder unspecified (F93.9)

only

STAndardised DIagnostic Assessment for children and adolescents with emotional difficulties (STADIA): A multi-centre randomised controlled trial

OUTCOME DEFINITION & ADJUDICATION PLAN

Final 1.0 – 25 February 2020

EMOTIONAL DISORDER DIAGNOSES RECORDED IN THE 12 MONTHS POST-RANDOMISATION

CONSTITUTES A CLINICAL DIAGNOSIS	REFER FOR ADJUDICATION	DOES NOT CONSTITUTE A CLINICAL DIAGNOSIS
- The presence of an eligible diagnosis within the diagnosis tab of the clinical record.<br>- The presence of an eligible diagnosis in the clinical record preceded by the heading 'diagnosis'.<br>- The presence of an eligible diagnosis in the clinical record preceded by a heading such as 'current difficulties' or 'presenting problems', except where this has been documented in the write up of the first appointment or in reference to the information received at referral (as this may simply reflect a pre-existing or referrer-made diagnosis).<br>- A clear confirmatory statement including use of an eligible diagnosis, for example:<br>*Meets the diagnostic criteria for…*<br>*Presentation is explained by a diagnosis of…*	- The presence of similar diagnostic terms within the diagnosis tab of the clinical record.<br>- The presence of an eligible diagnosis preceded by a heading such as 'current difficulties' or 'presenting problems', documented in the write up of the first appointment or in reference to the information received at referral.<br>- The presence of similar diagnostic terms preceded by a heading such as 'diagnosis', 'current difficulties' or 'presenting problems'.<br>- Reference to an eligible diagnosis or similar diagnostic terms, but where the context does not provide a clear confirmatory statement, for example:<br>*?...*<br>*Possible…*<br>*Assessed for…*<br>*…-type symptoms / behaviour*<br>*…-like symptoms / behaviour*<br>*Symptoms of…*<br>*History of…*	- No reference to an eligible diagnosis or similar diagnostic terms.<br>- A clear statement about the absence of an eligible diagnosis or similar diagnostic terms, for example:<br>*No evidence of…*<br>*…not meeting criteria for disorder*
Data collection and entry: instructions for researchers		
Use the checklist of eligible emotional disorder diagnoses.	*Document these as other emotional disorders.*	*Check 'none of the above' in the checklist of emotional disorder diagnoses and answer 'no' to 'other emotional disorder diagnoses'.*

Note: For definition of underlined terms see the Glossary below.

For peer review only - http://bmjopen.bmj.com/site/about/guidelines.xhtml

NO EMOTIONAL DISORDER

If there are no emotional disorder diagnoses documented in the CAMHS notes in 12 months post-randomisation, researchers will select one of the following options:

1. A clinician has documented the absence of emotional disorder.
2. Uncertainty about the presence of an emotional disorder is documented in the notes (for example, reflecting ongoing assessment / investigation).
3. There is no diagnostic information relating to emotional disorders documented in the CAMHS record.

**A clinician has documented the absence of emotional disorder.**	**Uncertainty about the presence of an emotional disorder is documented in the notes (for example, reflecting ongoing assessment / investigation).**	**There is no diagnostic information relating to emotional disorders documented in the CAMHS record.**
- Nothing in the clinical record is assessed to constitute a documented clinical diagnosis, AND<br>- There is a clear statement about the absence of one or more of the eligible diagnoses or similar diagnostic terms, for example:<br><br>*No evidence of…*<br>*…not meeting criteria for disorder*	- Nothing in the clinical record is assessed to constitute a documented clinical diagnosis, AND<br>- Reference to an eligible diagnosis or similar diagnostic terms, but where the context does not provide a clear confirmatory statement, for example:<br><br>*?...*<br>*Possible…*<br>*Assessed for…*<br>*…-type symptoms / behaviour*<br>*…-like symptoms / behaviour*<br>*Symptoms of…*<br>*History of…*	- Nothing in the clinical record is assessed to constitute a documented clinical diagnosis, AND<br>- There is no reference to an eligible diagnosis or similar diagnostic terms.*<br>- If emotional difficulties are identified they are described only by reference to the presenting symptoms with no attempt made to link these to an eligible diagnosis, for example:<br><br>*Presenting issue - Mood swings*<br>*Describing examples of ruminating thoughts.*<br><br>* Note that this includes children/young people who have not been seen by CAMHS in the 12-months post-randomisation.
Document these as absence of emotional disorder.	**REFER FOR ADJUDICATION**	**MAY REQUIRE ADJUDICATION**

**EMOTIONAL DISORDER DIAGNOSIS ADJUDICATION OUTCOME**	
The Adjudication Committee will first consider whether the record:<br>1) Constitutes a clinical diagnosis<br>2) Does not constitute a clinical diagnosis	If (1) then the Adjudication Committee will determine which of the eligible emotional disorder diagnoses apply.<br><br>If (2) then the Adjudication Committee will determine whether the record constitutes:<br>a) Absence of emotional disorder<br>b) Uncertainty about the presence of emotional disorder<br>c) No diagnostic information

For peer review only - http://bmjopen.bmj.com/site/about/guidelines.xhtml

TREATMENTS / INTERVENTIONS GIVEN

CONSTITUTES A TREATMENT / INTERVENTION	REFER FOR ADJUDICATION
- The presence of an eligible treatment / intervention documented within the clinical record.	- Documented intervention by CAMHS where the description does not include an eligible treatment / intervention.
Data collection and entry: instructions for researchers	
Use the checklist of eligible treatments / interventions.	*Document these as other treatments / interventions.*

TREATMENTS / INTERVENTIONS ADJUDICATION OUTCOME	
The Adjudication Committee will first consider whether the record:<br>1) Constitutes a treatment / intervention<br>2) Does not constitute a treatment / intervention	If (1) then the Adjudication Committee will determine whether the record should be categorised:<br>a) As an existing treatment / intervention<br>b) As an 'other' treatment / intervention

GLOSSARY	
Eligible diagnosis	One of the pre-specified diagnoses listed on the data collection form. These should be considered present only when the exact phrase and/or corresponding ICD/DSM code is documented.
Similar diagnostic terms	Words or phrases which are similar to the eligible diagnoses, but without use of the exact wording or corresponding ICD/DSM code (e.g., separation anxiety WITHOUT use of the term disorder) or where the exact words are used alongside additional phrases (e.g., OCD-type behaviour or OCD-like symptoms).
Eligible treatment / intervention	One of the pre-specified treatments / interventions listed on the data collection form.
Adjudication Committee	The Adjudication Committee will comprise the clinician members of the Trial Management Group. A minimum of two clinicians will review terms referred for adjudication, with a third consulted if a consensus is not reached.<br>The Adjudication Committee will be blinded to treatment allocation for the purposes of adjudication.

For peer review only - http://bmjopen.bmj.com/site/about/guidelines.xhtml

Appendix 5. Summary of assessments

Time-point	Maximum 10 working days from referral receipt[1]					6 months post-randomisation	12 months post-randomisation
**Activity**	**Screening and invitation**	**Eligibility and enrolment**	**Consent and baseline**			**Follow-Up**	
Initial eligibility screen of referral information	X			**Randomisation**	**Intervention**<br><br>DAWBA in addition to usual practice<br><br>Or<br><br>Usual practice only		
Telephone invitation to participate	X						
Verbal agreement to participate		X					
Confirm eligibility		X					
Obtain enrolment data		X					
Participant enrolment		X					
Written informed consent/assent (online)			X				
Baseline demographics (parent/carer and CYP aged 16 & 17)			X				
Mood and Feelings Questionnaire (MFQ)			X			X	X
Revised Child's Anxiety Depression Scale (RCADS)			X			X	X
Strengths and Difficulties Questionnaire (SDQ)[2]			X			X	X
Child Revised Impact of Events Scale (CRIES-8)(42)[3]			X			X	X
CYP self-report self-harm measure			X			X	X
Patient Health Questionnaire (PHQ-9) - parent/carer only			X			X	X
Generalised Anxiety Disorder Assessment (GAD-7) - parent/carer only			X			X	X
Child Health Utility 9D (CHU9D)			X			X	X
EuroQol-5D youth (EQ-5D-Y)			X			X	X
EuroQol-5D five level (EQ-5D-5L)			X			X	X
Resource Use Questionnaire - parent/carer and CYP aged 16 & 17			X			X	X
Data collection from records[4]			X			X	X

[1] For sites where the waiting time for the CAMHS acceptance decision usually exceeds 10 working days from referral receipt, recruitment activities may start and/or continue beyond 10 working days from referral receipt, providing the intervention period can be completed prior to the CAMHS referral decision.
[2] For participants in the intervention arm, the baseline SDQ will be collected as part of the DAWBA, completed post-randomisation.
[3] Additional data collection undertaken to explore post-traumatic stress disorder symptoms in CYP during the Covid-19 pandemic
[4] Data collection from records will be completed periodically throughout the 12 month follow-up period.

For peer review only

ADD LOCAL HEADER

STandardised DIagnostic Assessment for children and young people with emotional difficulties (STADIA)

Informed Consent Form for the Parent/Carer

Final v2.0 13 August 2020

Name of Principal Investigator: [add local PI name]

IRAS Project ID: 255635

Participant Trial ID:
(To be completed after randomisation)

We are doing this research to find out how to make sure children and young people get the help they need when they are referred to CAMHS. We have invited you to take part in this research because a young person you care for has been referred to CAMHS. You can decide whether or not to take part in this research.
If you agree to take part in the STADIA Trial, please read and acknowledge each of the following statements.

A drop-down menu will be provided within the online electronic Informed Consent Form so that the person providing consent has the option to acknowledge/agree to each of the following statements.	
**1.**	I confirm that I have read and understand the Participant Information Sheet, Version <insert current PIS version number and date > for the above research. (*Only for the parent/carer of children/young people aged 11-15*) [My child and] I have had the opportunity to consider the information, ask questions and have had these answered satisfactorily.
**2.**	*Only for the parent/carer of children/young people aged 11-15*<br>I have spoken to my child about the research and they are aware of the study.
**3.**	I understand that mine and my child's participation is voluntary and that I am free to withdraw at any time, without giving any reason, and without my child's medical care or legal rights being affected. I understand that should I withdraw, then the information collected so far cannot be deleted and that this information may still be used in the research.
**4.**	I understand that relevant sections of my child's CAMHS records and data collected in the trial may be looked at by authorised individuals from the Nottingham Clinical Trials Unit (University of Nottingham), the Sponsor (Nottinghamshire Healthcare NHS Foundation Trust), NHS bodies, the trial research group and regulatory authorities where it is relevant to taking part in this study. I give permission for these individuals to have access to these records and for my consent form to be retained by the Nottingham Clinical Trials Unit.
**5.**	I give permission for the Nottingham Clinical Trials Unit, the Sponsor and the trial research group to collect, store, analyse and publish information obtained from mine and my child's participation in this trial. I understand that our personal details will be kept confidential.
**6.**	I understand that the Nottingham Clinical Trials Unit and the trial research group will be provided with mine and my child's personal details to send questionnaires by email and study-related correspondence during the trial. I give my permission for this information to be kept and for these individuals to contact me.
**7.**	I understand that if I fill out the DAWBA, I will receive a copy of the DAWBA report and a copy will also be provided to the CAMHS team and kept in my child's CAMHS records.
**8.**	I agree to my child's GP being informed of their participation in this trial.
**9.**	I understand that the anonymised information collected about me and my child may be used to support other research in the future and may be shared with other researchers.
**10.**	I agree to take part in the above trial.

For peer review only - http://bmjopen.bmj.com/site/about/guidelines.xhtml

Please also answer yes or no to the following options.

A drop-down menu will be provided within the online electronic Informed Consent Form so that the person providing consent has the option to answer yes or no to each of the following optional statements.			
**1.**	*Interviews about your experiences*<br>I agree to be contacted about the STADIA interview study. I understand that there is no obligation to take part and I will just be informed of what the study will involve.	Yes	No
**2.**	*Future studies*<br>I agree to be contacted about other research studies in the future. I understand that there is no obligation to take part and I will just be informed of what the future research would involve.	Yes	No
**3.**	*Results of the STADIA study*<br>I would like to receive a summary of the results at the end of the STADIA study.	Yes	No
**4.**	*Only for the parent/carer of children/young people aged 11-15*<br>*Questionnaires*<br>I agree to my child being invited to complete questionnaires about their mood and feelings for the research.	Yes	No
**5.**	I consent to [INSERT NHS TRUST NAME] passing identifiable data (my child's NHS number, name and date of birth) to the organisations that are responsible for health information including NHS Digital. This will be used to request data from the Children and Young People's Health Services Data Set and the Mental Health Services Data Set.	Yes	No

Type your name here:

____________________________ _________________

Name of parent/carer Date [system generated]

Type the name of your child here:

____________________________ _________________

Name of child/young person Date [system generated]

System use only:

____________________________ _________________

Name of person taking consent Date [system generated]
(You must be on the delegation log)

NB. Signatures will not be collected as consent will be obtained online. Participants will be asked to complete the eICF and write their name before submitting the online form; the date will be system-generated. The name of the researcher who provided the study information and the date the eICF was generated will also be recorded within the online system.

The online electronic Informed Consent Form (eICF) will be retained within the trial database. Printable (PDF) copies will be generated and retained within the Investigator Site File and CAMHS records. A copy will be sent by email to the participant.

For peer review only - http://bmjopen.bmj.com/site/about/guidelines.xhtml

ADD LOCAL HEADER

STandardised DIagnostic Assessment for children and young people with emotional difficulties (STADIA)

Assent form for young people aged 11-15

Final v1.0 28-Mar-2019

Name of Principal Investigator: [add local PI name]

IRAS Project ID: 255635

Participant Trial ID: | | | | | |

(To be completed after randomisation)

We are doing this research to find out how to make sure people get the help they need when they are referred to CAMHS. We are asking you to help with this research but you can decide whether or not to take part.

If you agree to help with the STADIA Trial please answer the following questions.

A drop-down menu will be provided within the online electronic Assent Form so that the young person providing assent has the option to acknowledge/agree to each of the following statements.			
**1.**	Have you read the information about the research or has someone explained it to you?	Yes	No
**2.**	Do you understand what the research is about?	Yes	No
**3.**	Have you been able to ask all the questions you want?	Yes	No
**4.**	Do you understand that it's your choice whether or not to take part and it's OK to stop taking part at any time?	Yes	No
**5.**	Do you want to help with the research by completing some questionnaires about your mood and feelings?	Yes	No

Type your name here:

__________________________ Name of child/young person

_______________ Date [system generated]

System use only:

__________________________ Name of person taking consent
(You must be on the delegation log)

_______________ Date [system generated]

NB. Signatures will not be collected as consent will be obtained online. Participants will be asked to complete the eICF and write their name before submitting the online form; the date will be system-generated. The name of the researcher who provided the study information and the date the eICF was generated will also be recorded within the online system. The online electronic Informed Consent Form (eICF) will be retained within the trial database. Printable (PDF) copies will be generated and retained within the Investigator Site File and CAMHS records. A copy will be sent by email to the participant.

For peer review only - http://bmjopen.bmj.com/site/about/guidelines.xhtml

Reporting checklist for protocol of a clinical trial.

Based on the SPIRIT guidelines.

Instructions to authors

Complete this checklist by entering the page numbers from your manuscript where readers will find each of the items listed below.

Your article may not currently address all the items on the checklist. Please modify your text to include the missing information. If you are certain that an item does not apply, please write "n/a" and provide a short explanation.

Upload your completed checklist as an extra file when you submit to a journal.

In your methods section, say that you used the SPIRITreporting guidelines, and cite them as:

Chan A-W, Tetzlaff JM, Gøtzsche PC, Altman DG, Mann H, Berlin J, Dickersin K, Hróbjartsson A, Schulz KF, Parulekar WR, Krleža-Jerić K, Laupacis A, Moher D. SPIRIT 2013 Explanation and Elaboration: Guidance for protocols of clinical trials. BMJ. 2013;346:e7586

		Reporting Item	Page Number (line)
**Administrative information**			
Title	#1	Descriptive title identifying the study design, population, interventions, and, if applicable, trial acronym	1(1-2)

For peer review only

Trial registration	#2a	Trial identifier and registry name. If not yet registered, name of intended registry	2 (68)
Trial registration: data set	#2b	All items from the World Health Organization Trial Registration Data Set	Throughout
Protocol version	#3	Date and version identifier	18 (499)
Funding	#4	Sources and types of financial, material, and other support	19 (544-547)
Roles and responsibilities: contributorship	#5a	Names, affiliations, and roles of protocol contributors	1 (5-9)
Roles and responsibilities: sponsor contact information	#5b	Name and contact information for the trial sponsor	19 (562-563)
Roles and responsibilities: sponsor and funder	#5c	Role of study sponsor and funders, if any, in study design; collection, management, analysis, and interpretation of data; writing of the report; and the decision to submit the report for publication, including whether they will have ultimate authority over any of these activities	19 (544-550)
Roles and responsibilities: committees	#5d	Composition, roles, and responsibilities of the coordinating centre, steering committee, endpoint adjudication committee, data management team,	18 (515-523)

For peer review only

		and other individuals or groups overseeing the trial, if applicable (see Item 21a for data monitoring committee)	
**Introduction**			
Background and rationale	#6a	Description of research question and justification for undertaking the trial, including summary of relevant studies (published and unpublished) examining benefits and harms for each intervention	3-4 (91-148)
Background and rationale: choice of comparators	#6b	Explanation for choice of comparators	3-4, 7-8 (129-148)
Objectives	#7	Specific objectives or hypotheses	4 (150-176)
Trial design	#8	Description of trial design including type of trial (eg, parallel group, crossover, factorial, single group), allocation ratio, and framework (eg, superiority, equivalence, non-inferiority, exploratory)	4 (182-183)
**Methods: Participants, interventions, and outcomes**			

For peer review only

Study setting	#9	Description of study settings (eg, community clinic, academic hospital) and list of countries where data will be collected. Reference to where list of study sites can be obtained	5 (190-193)
Eligibility criteria	#10	Inclusion and exclusion criteria for participants. If applicable, eligibility criteria for study centres and individuals who will perform the interventions (eg, surgeons, psychotherapists)	5-6 (see table 1)
Interventions: description	#11a	Interventions for each group with sufficient detail to allow replication, including how and when they will be administered	7-8 (269-303)
Interventions: modifications	#11b	Criteria for discontinuing or modifying allocated interventions for a given trial participant (eg, drug dose change in response to harms, participant request, or improving / worsening disease)	n/a
Interventions: adherance	#11c	Strategies to improve adherence to intervention protocols, and any procedures for monitoring adherence (eg, drug tablet return; laboratory tests)	7-8 (269-303)
Interventions: concomitant care	#11d	Relevant concomitant care and interventions that are permitted or prohibited during the trial	7-8 (269-303)
Outcomes	#12	Primary, secondary, and other outcomes, including the specific measurement variable (eg, systolic blood pressure), analysis metric (eg, change from	8-14 (316-337)

For peer review only

		baseline, final value, time to event), method of aggregation (eg, median, proportion), and time point for each outcome. Explanation of the clinical relevance of chosen efficacy and harm outcomes is strongly recommended	
Participant timeline	#13	Time schedule of enrolment, interventions (including any run-ins and washouts), assessments, and visits for participants. A schematic diagram is highly recommended (see Figure 1)	5-7 (197-239) and see figure 1
Sample size	#14	Estimated number of participants needed to achieve study objectives and how it was determined, including clinical and statistical assumptions supporting any sample size calculations	8 (306-312)
Recruitment	#15	Strategies for achieving adequate participant enrolment to reach target sample size	5 (197-212)
**Methods: Assignment of interventions (for controlled trials)**			
Allocation: sequence generation	#16a	Method of generating the allocation sequence (eg, computer-generated random numbers), and list of any factors for stratification. To reduce	7 (242-246)

For peer review only

		predictability of a random sequence, details of any planned restriction (eg, blocking) should be provided in a separate document that is unavailable to those who enrol participants or assign interventions	
Allocation concealment mechanism	#16b	Mechanism of implementing the allocation sequence (eg, central telephone; sequentially numbered, opaque, sealed envelopes), describing any steps to conceal the sequence until interventions are assigned	7 (248-252)
Allocation: implementation	#16c	Who will generate the allocation sequence, who will enrol participants, and who will assign participants to interventions	7 (244-252)
Blinding (masking)	#17a	Who will be blinded after assignment to interventions (eg, trial participants, care providers, outcome assessors, data analysts), and how	7 (254-260)
Blinding (masking): emergency unblinding	#17b	If blinded, circumstances under which unblinding is permissible, and procedure for revealing a participant's allocated intervention during the trial	7 (254-256)
**Methods: Data collection, management, and analysis**			

For peer review only

Data collection plan	#18a	Plans for assessment and collection of outcome, baseline, and other trial data, including any related processes to promote data quality (eg, duplicate measurements, training of assessors) and a description of study instruments (eg, questionnaires, laboratory tests) along with their reliability and validity, if known. Reference to where data collection forms can be found, if not in the protocol	15 (356-363)
Data collection plan: retention	#18b	Plans to promote participant retention and complete follow-up, including list of any outcome data to be collected for participants who discontinue or deviate from intervention protocols	15 (359-362)
Data management	#19	Plans for data entry, coding, security, and storage, including any related processes to promote data quality (eg, double data entry; range checks for data values). Reference to where details of data management procedures can be found, if not in the protocol	15 (367-375)
Statistics: outcomes	#20a	Statistical methods for analysing primary and secondary outcomes. Reference to where other details of the statistical analysis plan can be found, if not in the protocol	15-17 (378-451)

For peer review only

Statistics: additional analyses	#20b	Methods for any additional analyses (eg, subgroup and adjusted analyses)	15-17 (378-451)
Statistics: analysis population and missing data	#20c	Definition of analysis population relating to protocol non-adherence (eg, as randomised analysis), and any statistical methods to handle missing data (eg, multiple imputation)	15 (378-380)
**Methods: Monitoring**			
Data monitoring: formal committee	#21a	Composition of data monitoring committee (DMC); summary of its role and reporting structure; statement of whether it is independent from the sponsor and competing interests; and reference to where further details about its charter can be found, if not in the protocol. Alternatively, an explanation of why a DMC is not needed	18 (522-523)
Data monitoring: interim analysis	#21b	Description of any interim analyses and stopping guidelines, including who will have access to these interim results and make the final decision to terminate the trial	18 (520-523)
Harms	#22	Plans for collecting, assessing, reporting, and managing solicited and spontaneously reported adverse events and other unintended effects of trial interventions or trial conduct	18 (502-512)

For peer review only

Auditing	#23	Frequency and procedures for auditing trial conduct, if any, and whether the process will be independent from investigators and the sponsor	18 (520-523)
**Ethics and dissemination**			
Research ethics approval	#24	Plans for seeking research ethics committee / institutional review board (REC / IRB) approval	18 (497-499)
Protocol amendments	#25	Plans for communicating important protocol modifications (eg, changes to eligibility criteria, outcomes, analyses) to relevant parties (eg, investigators, REC / IRBs, trial participants, trial registries, journals, regulators)	18 (497-499)
Consent or assent	#26a	Who will obtain informed consent or assent from potential trial participants or authorised surrogates, and how (see Item 32)	6 (see table 2)
Consent or assent: ancillary studies	#26b	Additional consent provisions for collection and use of participant data and biological specimens in ancillary studies, if applicable	6 (table 2)
Confidentiality	#27	How personal information about potential and enrolled participants will be collected, shared, and maintained in order to protect confidentiality before, during, and after the trial	15 (372-375)

For peer review only

Declaration of interests	#28	Financial and other competing interests for principal investigators for the overall trial and each study site	19 (553)
Data access	#29	Statement of who will have access to the final trial dataset, and disclosure of contractual agreements that limit such access for investigators	15 (372-375)
Ancillary and post trial care	#30	Provisions, if any, for ancillary and post-trial care, and for compensation to those who suffer harm from trial participation	n/a
Dissemination policy: trial results	#31a	Plans for investigators and sponsor to communicate trial results to participants, healthcare professionals, the public, and other relevant groups (eg, via publication, reporting in results databases, or other data sharing arrangements), including any publication restrictions	18 (526-527)
Dissemination policy: authorship	#31b	Authorship eligibility guidelines and any intended use of professional writers	18-19 (537-541)
Dissemination policy: reproducible research	#31c	Plans, if any, for granting public access to the full protocol, participant-level dataset, and statistical code	18 (530-531)

Appendices

For peer review only

Informed consent materials	#32	Model consent form and other related documentation given to participants and authorised surrogates	Supplementary materials 6 & 7
Biological specimens	#33	Plans for collection, laboratory evaluation, and storage of biological specimens for genetic or molecular analysis in the current trial and for future use in ancillary studies, if applicable	n/a

None The SPIRIT Explanation and Elaboration paper is distributed under the terms of the Creative Commons Attribution License CC-BY-NC. This checklist can be completed online using https://www.goodreports.org/, a tool made by the EQUATOR Network in collaboration with Penelope.ai

For peer review only

BMJ Open

STAndardised DIagnostic Assessment for children and young people with emotional difficulties (STADIA): protocol for a multi-centre randomised controlled trial

Journal:	*BMJ Open*
Manuscript ID	bmjopen-2021-053043.R2
Article Type:	Protocol
Date Submitted by the Author:	10-Mar-2022
Complete List of Authors:	Day, Florence; University of Nottingham, Nottingham Clinical Trials Unit, School of Medicine<br>Wyatt, Laura; University of Nottingham, Nottingham Clinical Trials Unit, School of Medicine<br>Bhardwaj, Anupam; Cambridgeshire and Peterborough NHS Foundation Trust<br>Dubicka, Bernadka; The University of Manchester; Pennine Care NHS Foundation Trust<br>Ewart, Colleen; University of Nottingham, STADIA Patient and Public Involvement co-lead, Institute of Mental Health<br>Gledhill, Julia; Central and North West London NHS Foundation Trust<br>James, Marilyn; University of Nottingham, Nottingham Clinical Trials Unit, School of Medicine<br>Lang, Alexandra; University of Nottingham, Faculty of Engineering<br>Marshall, Tamsin; Berkshire Healthcare NHS Foundation Trust<br>Montgomery, Alan; University of Nottingham, Nottingham Clinical Trials Unit, School of Medicine<br>Reynolds, Shirley; University of Reading, School of Psychology and Clinical Language Sciences<br>Sprange, Kirsty; University of Nottingham, Nottingham Clinical Trials Unit, School of Medicine<br>Thomson, Louise; University of Nottingham, Institute of Mental Health, School of Medicine<br>Bradley, Ellen; University of Nottingham, Institute of Mental Health, School of Medicine<br>Lathe, James; University of Nottingham, Nottingham Clinical Trials Unit, University of Nottingham<br>Newman, Kristina; Nottinghamshire Healthcare NHS Foundation Trust, Institute of Mental Health<br>Partlett, Chris; University of Nottingham, Nottingham Clinical Trials Unit, School of Medicine<br>Starr, Kath; University of Nottingham, Nottingham Clinical Trials Unit, School of Medicine<br>Sayal, Kapil; University of Nottingham, Institute of Mental Health, School of Medicine; Nottinghamshire Healthcare NHS Foundation Trust, Institute of Mental Health
**Primary Subject Heading**:	Mental health

Secondary Subject Heading:	Mental health
Keywords:	Child & adolescent psychiatry < PSYCHIATRY, Depression & mood disorders < PSYCHIATRY, HEALTH ECONOMICS

SCHOLARONE™
Manuscripts

STAndardised DIagnostic Assessment for children and young people with emotional difficulties (STADIA): protocol for a multi-centre randomised controlled trial

Authors:
Florence Day[1*], Laura Wyatt[1*], Anupam Bhardwaj[2], Bernadka Dubicka[3], Colleen Ewart[4], Julia Gledhill[5], Marilyn James[1], Alexandra Lang[6], Tamsin Marshall[7], Alan Montgomery[1], Shirley Reynolds[8], Kirsty Sprange[1], Louise Thomson[9], Ellen Bradley[9], James Lathe[1], Kristina Newman[10], Chris Partlett[1], Kath Starr[1] & Kapil Sayal[9,10]
***joint first authors**

Corresponding author:
Professor Kapil Sayal
Institute of Mental Health
School of Medicine
University of Nottingham
Nottingham, NG8 1BB

Email: kapil.sayal@nottingham.ac.uk

Word count, excluding title page, abstract, references, figures and tables: 4505

1 Nottingham Clinical Trials Unit, School of Medicine, University of Nottingham, Nottingham NG7 2RD
2 Cambridge and Peterborough Foundation Trust, Brookside CAMHS, Cambridge CB2 8AH
3 Pennine Care NHS Foundation Trust, Ashton-under-Lyne OL6 7SR
4 STADIA Patient and Public Involvement co-lead, Institute of Mental Health, University of Nottingham, Nottingham NG8 1BB
5 Central and North West London NHS Foundation Trust, Harrow CAMHS, Ash Tree Clinic, South Harrow HA2 8EQ
6 Faculty of Engineering, University of Nottingham, Nottingham NG7 2RD
7 Berkshire Healthcare NHS Foundation Trust, Fitzwilliam House, Bracknell RG12 1BQ
8 School of Psychology and Clinical Language Sciences, University of Reading, Reading RG6 6DZ
9 Institute of Mental Health, School of Medicine, University of Nottingham, Nottingham NG8 1BB
10 Nottinghamshire Healthcare NHS Foundation Trust, Institute of Mental Health, Nottingham NG8 1BB

For peer review only

ABSTRACT

Introduction

Emotional disorders (such as anxiety and depression) are associated with considerable distress and impairment in day-to-day function for affected children and young people and for their families. Effective evidence-based interventions are available but require appropriate identification of difficulties to enable timely access to services. Standardised Diagnostic Assessment (SDA) tools may aid in the detection of emotional disorders, but there is limited evidence on the utility of SDA tools in routine care and equipoise amongst professionals about their clinical value.

Methods and analysis

A multi-centre, two-arm, parallel group RCT, with embedded qualitative and health economic components. Participants will be randomised in a 1:1 ratio to either the Development and Wellbeing Assessment (DAWBA) SDA tool as an adjunct to usual clinical care, or usual care only.

A total of 1,210 participants (Children and Young People referred to outpatient, specialist Child and Adolescent Mental Health Services (CAMHS) with emotional difficulties and their parent/carers) will be recruited from at least 6 sites in England.

The primary outcome is a clinician-made diagnosis about the presence of an emotional disorder within 12-months of randomisation. Secondary outcomes include referral acceptance, diagnosis and treatment of emotional disorders, symptoms of emotional difficulties and comorbid disorders and associated functional impairment.

Ethics and dissemination

The study received favourable opinion from the South Birmingham Research Ethics Committee (Ref. 19/WM/0133). Results of this trial will be reported to the funder and published in full in the HTA Journal series and also submitted for publication in a peer reviewed journal.

Registration details

The STADIA trial was prospectively registered as ISRCTN15748675 on 29 May 2019.

Keywords

RCT; CAMHS; standardised diagnostic assessment; DAWBA; emotional disorders; diagnosis; outcomes; health economics; cost effectiveness; cost utility.

ARTICLE SUMMARY

Strengths and limitations of this study

- Large real-world multicentre randomised controlled trial of the DAWBA SDA tool as an adjunct to usual care versus usual care only.
- Trial procedures are carried out remotely with all data collection and the DAWBA completed online or via telephone, facilitating post-trial implementation into future service delivery models and routine clinical care.
- The embedded health economic component permits evaluation of both clinical and cost effectiveness.
- Embedded qualitative work will support optimal delivery and implementation to enhance acceptability, effectiveness and long-term uptake.
- Participants, researchers and clinicians cannot be blinded to treatment allocation.

For peer review only - http://bmjopen.bmj.com/site/about/guidelines.xhtml

INTRODUCTION

Emotional disorders cause considerable distress for affected Children and Young People (CYP) and their families, with adverse effects on family and peer relationships, quality of life, social involvement and activities, academic attainment and occupational opportunities, ultimately affecting life chances.(1-4) Emotional disorders are frequently comorbid with other disorders (2, 5), and are associated with self-harm and completed suicide. Effective evidence-based interventions are available but require appropriate identification of presenting difficulties to enable timely access to services and earlier recovery.(3)

The prevalence of emotional disorders has increased considerably over the past two decades.(1) In the UK, CYP with clinically significant emotional difficulties may be referred to outpatient specialist Child and Adolescent Mental Health Services (CAMHS). However, insufficient information is a common reason for referrals being declined.(6) There is limited evidence to inform optimal approaches to determine which referrals should be accepted, contributing to a large variation in acceptance rates.(6) Likewise there is a lack of evidence on how best to conduct assessments for suspected emotional difficulties to optimise outcomes. Acceptance criteria and assessment procedures differ across services and there is no single standardised approach.

The multi-disciplinary nature of CAMHS means CYP are assessed by practitioners from different professional backgrounds, with variations in training, ethos and conceptualisations of presenting difficulties. The type and scope of assessments offered vary. Assessments are often conducted by practitioners without formal diagnostic training(7) and recording of potential diagnostic information can be influenced by patient, clinician and service related contextual considerations(8). The validity and value of mental health diagnoses have been questioned, reflecting concerns around restricting service access (9), stigma or labelling.(7, 10, 11) This can mean that in routine practice, assessments are often undertaken without the aim of making or recording a diagnosis.

However, NICE guidelines for management and treatment are usually based on diagnostic classification of disorders, so the ability to offer evidence-based interventions requires that the CYP's difficulties are appropriately identified. Although NICE Quality Standards(12) state that CYP with suspected depression should have the diagnosis confirmed and recorded, this is highly variable in practice.(7, 13) The use of diagnostic assessments has been recommended so that important problems are detected and appropriate interventions are offered.(3, 11) The NICE guidelines for depression have recommended the use of standardised diagnostic assessment (SDA) tools as potential adjuncts in the detection of depression within CAMHS.(14) It has further been recommended that SDA tools should be used as an adjunct to clinical assessments, potentially at the point of referral receipt, to enable the allocation of cases to the most appropriate professional.(10, 15, 16)

One such SDA tool is the Development and Well-Being Assessment (DAWBA), a structured package of questionnaires and interviews which can be completed online or by telephone and yields algorithm-based diagnostic information.(17) The DAWBA has established reliability and validity (17) and has been widely used for screening, diagnosis and outcome measurement in research in both clinical and community settings (18, 19), including trials of SDAs (20, 21) and large scale epidemiological research.(1, 22, 23) A previous randomised controlled trial (RCT) using the DAWBA highlighted that, for emotional disorders, disclosing DAWBA diagnosis information to clinicians can improve the level of agreement between the DAWBA and clinical diagnoses, suggesting that the DAWBA can aid clinical detection of emotional disorders.(21) It also improved detection of comorbid disorders. A UK trial found higher levels of agreement between DAWBA and clinical diagnoses, following disclosure of DAWBA information, in relation to anxiety disorders.(20) Practitioners

For peer review only - http://bmjopen.bmj.com/site/about/guidelines.xhtml

acknowledged that the additional information could supplement the assessment and aid detection of difficulties.(10)

Hence, it might be expected that the introduction of an SDA tool following CAMHS referral receipt could enable resources to be better targeted and a timely conclusion to assessments with a diagnostic decision, increase the likelihood that an appropriate evidence-based treatment is offered, and lead to improved outcomes and better experience of care for CYP and their families. However, there is limited evidence on the utility of SDA tools for informing optimal approaches to assessment within routine clinical practice.

Aims and Objectives

The aim is to evaluate the clinical and cost effectiveness of the DAWBA SDA tool, as an adjunct to usual clinical care for CYP presenting with emotional difficulties referred to CAMHS.

Specific objectives are to:

1. Conduct an RCT to determine the effectiveness of the DAWBA as an adjunct to usual clinical care on diagnosis and treatment of emotional disorders, symptoms of emotional difficulties and comorbid disorders and associated functional impairment.

2. Undertake an internal pilot to assess recruitment and acceptability.

3. Include a qualitative component within the pilot phase to address:
   a) The feasibility of recruitment.
   b) The acceptability and usability of the interventions and procedure.
   c) How the intervention is used and could be refined for the main trial.

4. Conduct a process evaluation alongside the main trial which will:
   a) Optimise the design and delivery of the DAWBA to enhance acceptability, effectiveness and long-term uptake.
   b) Identify the barriers and facilitators to implementation of the DAWBA from the perspectives of CYP, parents, and CAMHS practitioners, managers and commissioners.

5. Estimate cost effectiveness of the use of the DAWBA versus usual care.

6. Make evidence-based recommendations for assessment procedures within CAMHS and produce practice guidelines for clinical decision-making around the referral acceptance and assessment processes.

METHODS AND ANALYSIS

Design

A multi-centre, two-arm, parallel group RCT, with embedded qualitative and health economic components.

An internal pilot period, completed in the first 9 months of recruitment, will determine feasibility of recruitment and follow-up, assessed by the independent Trial Steering Committee against pre-defined stop/go criteria. The study start date is 01-Nov-2018 and end date is 31-Oct-2022.

Setting

For peer review only - http://bmjopen.bmj.com/site/about/guidelines.xhtml

Recruitment will take place in at least six NHS Trusts in England, providing outpatient multidisciplinary specialist CAMHS. Sites are geographically dispersed covering urban and rural areas, thus are likely to be socio-demographically representative of CAMHS referrals in England, enabling nationally generalisable findings.

Recruitment and eligibility

Participant identification

The population is CYP presenting with emotional difficulties referred to CAMHS. Participants are identified through the usual referral pathways for the participating sites, which includes NHS and local authority managed Single/Central Point of Access referral points as well as referrals directly received and processed by CAMHS teams.

The STADIA researchers (NHS personnel, based within the CAMHS SPA/triage team to carry out research activities on behalf of the team and authorised to access referral information) at each site review the referrals received by CAMHS to identify CYP presenting with emotional difficulties, according to a standard proforma (Appendix 1. Screening form). Referrals that mentioned any current emotional difficulties will be included, regardless of the number, frequency or severity of the emotional difficulties. Potentially eligible participants are invited to consider taking part in the trial and provided with written information. The initial invitation follows standardised wording to ensure clarity and consistency of approach.

Identification of participants takes place after referral receipt, but prior to referral acceptance (Figure 1).

Consent

Prior to consent, eligibility will be confirmed (table 1) during telephone contact with the local STADIA researcher, who will also provide written and verbal information about the trial, answer questions and support the electronic consent/assent process. Participants who are eligible and provide verbal consent to participation during the call will be provided with a personal link to the online electronic Informed Consent/Assent Form (table 2, appendix 2 and 3, respectively), enabling them to provide written informed consent/assent.

Table 1. Eligibility criteria

Inclusion criteria for the CYP<br>• Aged 5 to 17 years.<br>• Referred to outpatient multidisciplinary specialist CAMHS.<br>• Presenting with emotional difficulties.<br>• If aged <16, has an eligible individual with parental responsibility (see parent/carer eligibility criteria below) willing and able to participate in the trial.<br>• If aged 16-17, has capacity to provide valid written informed consent.<br>• If aged 16-17 and participating without a parent/carer, able to complete the assessment tool in English.<br>• If aged 16-17 and participating without a parent/carer, access to internet and email or telephone.
Exclusion criteria for the CYP<br>• Emergency or urgent referral to outpatient multidisciplinary specialist CAMHS (i.e. requires an expedited assessment) according to local risk assessment procedures.<br>• Severe learning disability.<br>• Previously randomised in the STADIA trial.
Inclusion criteria for the parent/carer

For peer review only - http://bmjopen.bmj.com/site/about/guidelines.xhtml

For peer review only

- Individual with parental responsibility for the CYP referred to CAMHS; this will be the CYP's mother or father, legally appointed guardian or a person with a residence order concerning the CYP.
- Adequate knowledge of the CYP to be able to complete the assessment tool (i.e., known for at least 6 months).
- Has capacity to provide valid written informed consent.
- Access to internet and email or telephone.
- Able to complete the assessment tool in English.

Exclusion criteria for the parent/carer

- Local authority representatives designated to care for the CYP.

The participation and consent/assent requirements for the trial are shown in table 2.

Table 2: Consent & Participation

**Age of CYP referred to CAMHS:**	**CYP aged <11**	**CYP aged 11-15**		**CYP aged 16-17**	
**Initial contact with:**	Parent/carer			Depends on contact details provided with the CAMHS referral*	
**Consent provided by:**	Parent/carer	Parent/carer	Parent/carer	CYP AND parent/carer (optional)	CYP
**Assent provided by:**	None	CYP (optional)	None	None	None
**Participant(s):**	Parent/carer only	CYP and parent/carer dyad	Parent/carer only	CYP and parent/carer dyad	CYP only
**Primary participant:****	Parent/carer	Parent/carer	Parent/carer	CYP	CYP
**Secondary participant:**	None	CYP	Non	Parent/carer	None
**DAWBA completed by:**	Parent/carer	Parent/carer AND CYP	Parent/carer	CYP AND parent/carer	CYP
**Research questionnaires completed by:**	Parent/carer report on CYP<br>Parent/carer self-report	Parent/carer report on CYP<br>Parent/carer self-report<br>CYP self-report	Parent/carer report on CYP<br>Parent/carer self-report	CYP self-report<br>Parent/carer report on CYP<br>Parent/carer self-report	CYP self-report

For all CYP aged <16 the initial contact about the study will be with the parent/carer. The involvement of CYP aged 11-15 will be at the discretion of the parent/carer.
* For CYP aged 16-17 if the CYP's contact details are provided on the CAMHS referral the first contact about the study will be with the CYP who can choose to nominate a parent/carer to participate in the trial alongside them or participate alone. If the parent/carer's contact details only are available the first contact will be with the parent/carer and the parent/carer will be asked whether the CYP can also be contacted but may choose to refuse this. The parent/carer will not be able to participate in the STADIA trial without the involvement or consent of the CYP.
** The primary participant is the person who must provide consent as a minimum requirement in order for randomisation to take place. Assent (of CYP aged 11-15) and parental consent (for CYP aged 16 and 17) may also be sought but is not mandatory and therefore will not be required prior to

For peer review only

For peer review only - http://bmjopen.bmj.com/site/about/guidelines.xhtml

randomisation.

Participants are free to withdraw at any time and for any reason. Participants may withdraw from the intervention, follow-up questionnaires and/or data collection from records in any combination (e.g., participants who do not complete the intervention will continue to be followed-up, participants withdrawing from follow-up questionnaire completion may continue to consent for data collection from records). Withdrawn participants will not be replaced. Data collected prior to withdrawal will be retained and used in the analysis.

Where CYP aged 16 or 17 have consented for their own involvement they can continue to participate in the trial in the event of their parent/carer's withdrawal, however, the parent/carer involvement would not continue should the CYP withdraw consent.

Randomisation and concealment

Participants will be randomised in a 1:1 ratio to either intervention or control. Allocation will be assigned using a minimisation algorithm balancing on recruiting site, CYP age (5-10, 11-15, 16-17 years) and sex, incorporating a probabilistic element to allocation. The allocation algorithm was created by Nottingham Clinical Trials Unit (NCTU) in accordance with their Standard Operating Procedures (SOPs). Allocation is concealed using an automated web system operated by NCTU.

Randomisation is automatically generated within the online system following submission, and automated verification, of baseline data by the primary participant. Participants are presented with their allocation and further instructions on-screen with email confirmation. Instructions for DAWBA completion are included for those in the intervention arm. Email confirmation is sent to the coordinating centre and site research team.

It will not be possible to blind participants, site researchers, clinicians and some trial staff to treatment allocation, but treatment allocation data will be restricted to those trial staff who require access to facilitate trial conduct. In particular, it will not be fully possible to blind researchers conducting data collection from records. However, any possible diagnoses identified from the CAMHS records will be recorded verbatim on the data capture form and will be subject to adjudication by the trial adjudication committee (members of the Trial Management Group). The committee will be blinded to treatment allocation and participant ID.

The risk of contamination between arms is considered low. Access to the DAWBA, and provision of the DAWBA report, is only provided to participants in the intervention arm. SDA tools are not current practice in standard care and it is unlikely that control participants will be asked to complete these at the point of referral receipt. DAWBA completion occurring outside the trial for control arm participants will be collected during follow-up.

Interventions

Development and wellbeing assessment (DAWBA)

The trial intervention is the DAWBA. (24) The DAWBA has a modular structure, with only those modules relevant to emotional and comorbid disorders included; separation anxiety, specific phobia, social phobia, panic and agoraphobia, generalised anxiety, post-traumatic stress disorder (PTSD), obsessive compulsive disorder (OCD), depression, oppositional defiant disorder (ODD) and conduct disorder. Whereas, the strengths and difficulties questionnaire, bipolar disorder, and body dysmorphic disorder are not included in the STADIA-specific DAWBA report as these modules do not generate diagnostic predictions. No freetext responses are collected.

For peer review only - http://bmjopen.bmj.com/site/about/guidelines.xhtml

The DAWBA will be self-reported by participants via the secure, standalone online platform created and maintained by the DAWBA developer.(24) Access is by a unique ID number and password, assigned at the point of randomisation via a stock control system integrated into the randomisation system, ensuring accountability of DAWBA slot allocation.

The DAWBA may be completed by the parent/carer and/or CYP aged 11+, depending on the consent and participation arrangements (Table 2) DAWBA completion will be monitored and the STADIA researcher will support and encourage completion. Participants will be able to complete the DAWBA in a telephone call with the STADIA researcher if required. Participants are asked to complete all modules of the DAWBA presented to them. Should the DAWBA be only partially completed by respondents the report will be based only on fully answered modules with missing responses identified as such.

A trial-specific DAWBA report will be prepared for each participant, based on a standard, study-specific template (Appendix 4. Template DAWBA report). The algorithm-derived diagnostic predictions will be used to highlight the likelihood of a CYP meeting ICD-10 criteria for the disorders assessed; the report is based entirely on the algorithm-derived predictions and is not clinically rated. The report will be sent to participants (via post or email) and CAMHS clinicians (via upload to the clinical record), as an adjunct to usual clinical practice.

Control

CYP randomised to the control arm will receive usual care (i.e., referral review as usual). Based on standard information provided with the referral a clinical decision is made about whether the referral is accepted and, if so, a clinician conducts the initial CAMHS assessment as per usual practice in the service.

Sample size

A target sample size of 1210 participants will be recruited and randomised, with equal allocation to intervention or control.

Assuming 45% of control participants have a confirmed diagnosis within 12 months (based on unpublished data obtained from the trial sites), detection of an absolute increase of 10% with 90% power and 5% two-sided alpha, requires 544 participants per arm for analysis. Allowing for up to 10% non-collection of the primary outcome, we will randomise 1210 participants.

Measures and outcomes

Primary outcome

The primary outcome is a clinician-made diagnosis decision about the presence of an emotional disorder within 12 months of randomisation i.e. diagnosis of an emotional disorder will be coded as ‘yes’; absence or uncertainty (for example, reflecting ongoing assessment or investigation) will be coded as ‘no’. Eligible diagnoses are those that reflect ‘emotional’ or ‘internalizing’ disorders in ICD/DSM (Appendix 5. Eligible emotional disorder diagnoses). The diagnosis must be documented in the clinical record within 12 months of randomisation by a mental health services clinician in an NHS-delivered or NHS-commissioned service.

Diagnoses will be collected from clinical records using a standard proforma. Alternative possible diagnoses identified from the clinical notes will be recorded verbatim on the data capture form and will be subject to adjudication by members of the Trial Management Group (Appendix 6. Outcome Definition and Adjudication Plan).

For peer review only - http://bmjopen.bmj.com/site/about/guidelines.xhtml

Secondary outcomes

Secondary outcomes are detailed in table 3.

For peer review only

For peer review only - http://bmjopen.bmj.com/site/about/guidelines.xhtml

333 *Table 3. Secondary outcome definitions*
334

Outcome	Measurement	Definition
Acceptance of index referral	Collected from records	Whether the index referral (i.e., the referral made to CAMHS at the point of recruitment to the STADIA trial) was accepted or declined.<br><br>Acceptance is defined as being offered an appointment within CAMHS, whether or not the initial appointment was attended or subsequent appointments were offered/attended. Collected within 12 months of randomisation.
Acceptance of any referral within 12 months of randomisation	Collected from records	Whether the index referral or any subsequent referral to CAMHS (if made) was accepted or not .<br>Acceptance as defined above for index referral. Collected within 12 months of randomisation.
Discharge from CAMHS within 12 months	Collected from records	Whether the child/young person was discharged from CAMHS (following acceptance of the index referral) during the 12-months post-randomisation.
Re-referral to CAMHS within 12 months	Collected from records	Whether the child/young person was re-referred to CAMHS (for those whose index referral was turned down by CAMHS or those whose index referral was accepted but were subsequently discharged) during the 12-months post-randomisation.
Confirmed diagnosis decision	Collected from records	Diagnosis of an emotional disorder or confirmed absence of an emotional disorder coded as 'yes' vs. uncertainty about the presence of an emotional disorder coded as 'no'. Diagnosis as defined for primary outcome, collected within 12 months of randomisation.
Time from randomisation to diagnosis of emotional disorder	Collected from records	Date of diagnosis will be the first documented eligible diagnosis.Diagnosis as defined for primary outcome, collected within 12 months of randomisation.
Diagnoses made over the 12 month period from randomisation	Collected from records	The diagnosis must be documented in the clinical record within 12 months of randomisation by a mental health services clinician in an NHS-delivered or NHS-commissioned service. All diagnoses made within 12 months will be included. Measured using a standard proforma (pre-specified diagnoses).
Treatment offered for diagnosed emotional disorder	Collected from records	Whether treatment was offered for a diagnosed emotional disorder, as defined for primary outcome, collected within 12 months of randomisation.
Any treatment / interventions given	Collected from records	All treatments/interventions offered by CAMHS for any reason within 12 months of randomisation, whether or not there is a documented diagnosis will be included.

For peer review only - http://bmjopen.bmj.com/site/about/guidelines.xhtml

Outcome	Measurement	Definition
Time from randomisation to the decision to offer treatment for a diagnosed emotional disorder	Collected from records	Date of decision will be the first date that the decision to offer treatment for a diagnosed emotional disorder is documented in the clinical notes, collected within 12 months of randomisation.
Time from randomisation to start of first treatment for a diagnosed emotional disorder	Collected from records	Date of treatment will be the first date that any treatment offered for a diagnosed emotional disorder is started.<br><br>Treatment and diagnosed emotional disorder as defined, collected within 12 months of randomisation.
Time from randomisation to the decision to offer any treatment	Collected from records	Date of decision will be the first date that the decision to offer any treatment is documented in the clinical notes, , collected within 12 months of randomisation.
Time from randomisation to start of any treatment	Collected from records	Date of treatment will be the first date that any treatment offered is started.<br><br>Treatment as defined, collected within 12 months of randomisation.
Participant-reported diagnoses received in the 12 months post-randomisation	Participant self-report	Participants will be asked to report whether or not they received a diagnosis of the child/young person's difficulties from CAMHS in the 12 months post-randomisation and if so, what diagnosis was given and by whom.
Depression symptoms in the CYP	Mood and Feelings Questionnaire (MFQ)	Mood and Feelings Questionnaire (MFQ) (25) is a valid and reliable measure of depression in CYP.(26, 27)<br><br>33-items are answered on a 3-point scale ("not true" = 0, "somewhat true" = 1 point, "true" = 2 points).<br><br>Scores range from 0 to 66 with higher scores indicating more severe depressive symptoms. A score of 27 or higher may be indicative of depression. MFQ collected at baseline, 6 and 12 months post-randomisation.

For peer review only - http://bmjopen.bmj.com/site/about/guidelines.xhtml

Outcome	Measurement	Definition
Anxiety symptoms in the CYP	Revised CYP's Anxiety Depression Scale (RCADS)	Revised CYP's Anxiety and Depression Scale (RCADS)(28)<br>RCADS is a 47-item questionnaire that measures the reported frequency of various symptoms of anxiety and low mood. Each item is rated on a 4-point scale (never = 0, sometimes = 1, often = 2, always = 3).<br>An overall anxiety and low mood score is generated, with separate sub-scale scores for separation anxiety, social phobia, generalised anxiety, panic, obsessive compulsive disorder and major depression.<br>RCADS demonstrates good psychometric properties.(29)<br>Total anxiety and depression scores range from 0 to 141.<br><br>We will record scores for each of the 6 sub-scales. For analysis metric, we will use the total anxiety score. RCADS collected at baseline, 6 and 12 months post-randomisation.

For peer review only - http://bmjopen.bmj.com/site/about/guidelines.xhtml
For peer review only

Outcome	Measurement	Definition
Comorbid oppositional defiant / conduct disorder symptoms in the CYP	Strengths and Difficulties Questionnaire (SDQ)	Strengths & Difficulties Questionnaire (SDQ):(30) A 25-item emotional and behavioural screening questionnaire for CYP.<br><br>Each item is rated on a 3-point scale (not true, somewhat true, certainly true). Values of 0, 1 or 2 are assigned to each response.<br><br>SDQ comprises 5 sub-scales and an impact supplement. The impact supplement asks effect of difficulties on homelife, friendships, education and leisure activities.<br><br>SDQ has demonstrated reasonable psychometric properties.(31-34)<br>Scores on the 'conduct problems' subscale will be used in the analysis of this outcome.<br><br>Sub-scale scores range from 0 to 10. SDQ collected at baseline, 6 and 12 months post-randomisation.
Functional Impairment in the CYP	Strengths and Difficulties Questionnaire (SDQ)	Impact supplement scores will be used to determine functional impairment. Impact scores range from 0 to 10. Collected at baseline, 6 and 12-months post-randomisation.

For peer review only - http://bmjopen.bmj.com/site/about/guidelines.xhtml

Outcome	Measurement	Definition
Self-harm thoughts in the CYP	CYP self-report self-harm measure	CYP will be asked to report the frequency of thoughts of self-harm.<br>Frequency of thoughts of self-harm are rated over the last 6 months in the following categories and scored accordingly:<br>Not at all (0)<br>Once or twice (1)<br>Three or more times (2)<br>Collected at baseline, 6 months and 12-months post-randomisation.
Self-harm behaviours in the CYP	CYP self-report self-harm measure	CYP will be asked to report frequency of instances of self-harm behaviour.<br>Frequency of self-harm behaviour are rated over the last 6 months in the following categories and scored accordingly:<br>Not at all (0)<br>Once (1)<br>Two or more times (2)<br>Collected at baseline, 6 months and 12-months post-randomisation.
Depression symptoms in the parent/carer	Patient Health Questionnaire (PHQ-9)	PHQ-9:(35) PHQ-9 is frequently used as a screening tool for depression in general populations. Each of the nine DSM-IV depression criteria are scored as "0" (not at all) to "3" (nearly every day) depending on the frequency with which they were experienced over the last 2 weeks.<br>Total scores range from 0 to 27 with higher scores indicating increased severity of depression, collected at baseline, 6 and 12-months post-randomisation.
Anxiety symptoms in the parent/carer	Generalised Anxiety Disorder Assessment (GAD-7)	GAD-7:(36)<br>GAD-7 is a measure of the severity of anxiety in general populations. 7 items are rated according to the frequency with which they have been experienced over the past 2 weeks (0 = 'not at all', 1 = 'several days', 2 = 'more than half the days', and 3 = 'nearly every day').<br>Total scores range from 0 to 21 with higher scores indicating more severe anxiety.<br>Collected at baseline, 6 and 12-months post-randomisation.
Time off education, employment or training because of emotional difficulties for the CYP	Resource use questionnaire	Days missed from education, employment or training (as applicable) for the CYP due to emotional difficulties. Collected at baseline, 6 and 12-months post-randomisation.

For peer review only - http://bmjopen.bmj.com/site/about/guidelines.xhtml

Outcome	Measurement	Definition
Health economic outcome measures		
Health related quality of life in the CYP	Child Health Utility 9D (CHU9D) and EuroQol Quality of Life Questionnaire 5 Domains for Young People (EQ-5D-Y)	CHU9D (37) consists of nine individual items with five levels of response per question (scored 1-5), that assess the CYP functioning "today". The following domains are included; worry, sadness, pain, tiredness, annoyance, school, sleep, daily routine and activities.<br><br>EuroQol-5D youth descriptive system (38) comprises 5 domains; mobility, looking after myself, doing usual activities, having pain or discomfort and feeling worried, sad or unhappy, values of 1, 2 or 3 are assigned to each response. The EuroQol Visual Analogue Scale (EQ-VAS) asks recipients to self-assess their health state 'today' from 0 (worst imaginable health) to 100 (best imaginable health), representing individual preferences.<br><br>These measures will be self-reported by CYP aged 11+, with proxy versions also completed by the parent/carer for CYP <16.<br><br>Both collected at baseline, 6 and 12-months post-randomisation.
Health-related quality of life in the parent/carer	EuroQol Quality of Life Questionnaire 5 Domains, 5 Levels (EQ-5D-5L)	The EuroQol 5-dimension multi attribute utility instrument (39)comprises 5 domains; mobility, self-care, usual activities, pain/discomfort and anxiety/depression. Each domain is scored between 1 and 5. This descriptive profile, in combination with a valuation set, produces a single index for health status representing societal preferences. The index score ranges from -0.59 to 1, with 0 representing death, 1 of-perfect health, and <0 of health states worse than death. The EQ-VAS is again included within the EQ-5D instrument Collected at baseline, 6 and 12-months post-randomisation.

For peer review only - http://bmjopen.bmj.com/site/about/guidelines.xhtml

Health economic measures

Health related quality of life (HRQoL) outcome measures are detailed in table 3.

Resource Use

Data will be collected on health care, education, and social care resource use for both the CYP and parents/carers, using a purposely designed resource use collection tool. The questionnaire was developed by the study's health economics team at Nottingham following discussions with the study's Patient and Public Involvement (PPI) team and representatives. This was an iterative process until all parties including the PPI team and representatives, the health economics team and the wider Trial Management Group were reassured the questionnaire was fit for purpose. It collects data on all aspects of healthcare interventions including medication, inpatient and outpatient hospital visits and primary and community care use as well as societal and education costs. It also includes sections specifically designed to quantify the effect of time off work for parents/carers (including friends and family) to quantify the wider social cost i.e. implications for productivity. In addition, it measures effects on time lost from education or training for the child/young person because of emotional difficulties. A similar approach to capturing resource use information was employed by members of the study team for a feasibility trial involving parents and carers of children with ADHD (40).

These data will be attributable to the emotional difficulties of the young person and be self-reported by the parent/carer with supplementary information obtained from CYP aged 16 and 17. Administrative records of treatments/interventions offered by CAMHS during the trial period may be considered as a supplementary source of data.

Socio-demographic data

The following socio-demographic data will be collected primarily from the participant-reported questionnaires; age at randomisation, sex, gender, ethnicity, paid employment, and, derived from the postcode of the child's primary residence, the index of Multiple Deprivation score.

Data collection

Data will be collected through participant reported questionnaires (parent/carer and CYP self-report aged 11+) and from clinical records. Participant reported outcomes will be collected at baseline and 6- and 12-months post-randomisation (Appendix 7. Summary of assessments). Questionnaires are intended to be completed online by participants in the first instance - to maximise rates of completion and retention there will be an option for telephone completion, should participants have difficulty accessing or completing the questionnaires online.

Outcomes collected from records will be reported for the 12-month period following randomisation.

Data management and analysis

Data management

Arrangements for data handling are specified in the Data Management Plan (DMP). Central and on-site monitoring will be carried out as required following a risk assessment and as documented in the monitoring plan. Monitoring activities will be carried out by the coordinating centre on behalf of the trial sponsor.

Data will be held on servers located within The University of Nottingham data centres. Security is both physical (secure limited access) and electronic (behind firewalls, access via user accounts). Personal data recorded on all documents will be regarded as strictly confidential and handled and stored in accordance with the Data Protection Act 2018.

For peer review only

For peer review only - http://bmjopen.bmj.com/site/about/guidelines.xhtml

Statistical analysis

The primary approach to between-group comparative analyses will be by modified intention-to-treat (i.e. including all participants who have been randomised and without imputation of missing outcome data).

The primary comparative analysis will employ a generalised linear mixed model to compare the proportions in each group with a clinician-made diagnosis decision within 12 months of randomisation, adjusted for minimisation variables. The comparison will be presented as both an absolute (risk difference) and relative (risk ratio) effect, along with 95% confidence intervals.

Secondary outcomes will be analysed using appropriate mixed effect regression models dependent on data type and will adjust for factors used in the minimisation and baseline value of the outcome where measured. For outcomes measured at multiple time points, these will be analysed using a mixed model with a treatment by time interaction to obtain estimates of treatment effect at each follow-up time.

Appropriate interaction terms will be included in the primary regression analyses in order to conduct subgroup analyses according to sex and age of the CYP.

Statistical analysis will be conducted using Stata v17.0 (or later).

Health economic analysis

In accordance with NICE guidance, primary analysis will take an NHS and personal social services perspective. Unit costs will be attached to participant reports of health care resource use or recorded treatments/interventions offered by CAMHS. The cost of the DAWBA itself will be distributed at the participant-level across the intervention arm of the trial. Sensitivity analyses will take a wider perspective to capture the broader societal costs inclusive of out-of-pocket expenses and productivity losses. Indices of HRQoL for the EQ-5D, EQ-5D-Y, and CHU9D will be derived using relevant population tariffs, and quality adjusted life years estimated using area under the curve (AUC).

The economic evaluation will take an incremental approach between the two groups using an intention-to-treat (ITT) population (irrespective of treatment received) and a 12-month time horizon. The outcome for the primary cost utility analysis will be the joint young person and parent/carer QALYs. The outcome for the secondary cost effectiveness analysis will be confirmed diagnosis decisions. Outcomes will be paired with their respective direct-to-NHS costs, bootstrapped, and scattered on the cost effectiveness plane to characterise the uncertainty in incremental estimates. Using the net monetary benefit framework,(41) Cost Effectiveness Acceptability Curves (CEACs) will be constructed to show the non-parametric probability the intervention is a cost effective option, compared to usual care, across a range of willingness to pay thresholds per QALY, and within the secondary analysis per confirmed diagnosis decision. While the receipt of any diagnosis of emotional difficulties in young people would likely lead to large divergences in lifecourse outcomes, the heterogeneity of conditions considered for diagnosis (Appendix 5) renders CUA modelling across the lifecourse infeasible. Secondary analysis is expected to be fully captured within the 12-month time horizon.

A full statistical analysis plan (SAP) and health economics analysis plan (HEAP) will be developed and agreed prior to database lock and un-blinding of the analysing statistician and health economist.

Embedded qualitative study

For peer review only - http://bmjopen.bmj.com/site/about/guidelines.xhtml

During the internal pilot, semi-structured interviews are undertaken with a sample of participants who consented to be invited to participate in qualitative interviews. Researchers, clinicians, service managers and commissioners are identified by site leads. The proposed sample size is 25 participants (parent/carer and CYP aged 16-17), 25 staff and 15 service managers and commissioners. Interviews address: a) the feasibility of recruitment; b) the acceptability and usability of the interventions and procedure; c) how the intervention is used and how this deployment could be refined for the main trial. Interviews are conducted by the qualitative researcher (KN) in person, or by phone or video call based on participant preferences and pandemic restrictions.

A process evaluation, conducted during the main trial phase, will aim to identify the barriers and facilitators to implementation of the intervention. Semi-structured interviews will be conducted with a further sample of participants and clinicians to explore the perceived functioning of the intervention, the organisation of the service and reflective experiences on outcomes.

Qualitative interview data will be recorded and encrypted on a password protected Dictaphone and transferred securely to medical transcription company Dict8 for transcription. Transcriptions will be anonymised. Audio files will be destroyed after transcripts have been checked. Anonymised transcriptions will be analysed and stored on password protected computers and the secure University of Nottingham server.

Qualitative analysis

All qualitative interview data will be initially analysed by the qualitative researcher (KN) using interpretative thematic approaches to coding, and adopt the framework method,(42) with input from the qualitative lead (LT), Chief Investigator (KSa) and PPI leads (CE & AL). NVIVO 12 will be used to manage the qualitative data.

Patient and public involvement

Prior to submission, the proposal was informed by consultations with a person with lived parent/carer experience of CAMHS, including contribution to and review of the proposal, recruitment strategy, participant trial experience and consideration of burden of the intervention, and establishing a PPI workstream.

Following award, the PPI Co-I team recruited two representatives naïve of the study design to provide independent review of the trial via their membership of the Trial Steering Committee (TSC). Both TSC members are persons with lived parent/carer experience of CAMHS.

During study set up, PPI Co-I expertise was utilised to support researcher recruitment via the design and deployment of role plays within interviews.(43) This was to gain insight into candidates' capabilities when dealing with sensitive and challenging participant scenarios. Additionally, they contributed to design of researcher training materials, to support standardised approaches across trial sites. Iterative and creative design PPI activities were integral in the development of the STADIA trial logo and branding to ensure accessibility and acceptability to CYP and parents.

Since study commencement participatory design approaches have seen PPI co-design of the resource use questionnaire, qualitative interviews and the protocol for a Study Within A Trial (SWAT) to support participant engagement with follow-up. Additionally, collaborative working between the PPI and Qualitative workstreams has enabled examination of the qualitative themes using principles of the Framework Method(42) for independent verification of those themes.

Two PPI advisory panels have been established, meeting on average every 3 months since month 9 of the study. "STADIA PPI Panel" has 8 adult members, with lived parent/carer experience of

For peer review only - http://bmjopen.bmj.com/site/about/guidelines.xhtml

CAMHS. “STADIA Labs” has 6 CYP members, aged 15 to 19 at inception, with lived experience of CAMHS. These groups have been involved in many traditional activities such as review of PIS and consent forms, consultation on language and content for participant reminder text messages. PPI co-production activities are also seeing the development of age appropriate study newsletters and the design of STADIA information videos including decision making about video concept, audience, message, aesthetic and content. PPI group members are provided with supplementary training about PPI practices and involvement opportunities. Due to the Covid-19 pandemic, PPI meetings have had to move online and so the PPI team are investing in knowledge transfer and upskilling PPI representatives in different ways of working and collaborating online.

There are a range of planned flexible opportunities for participating in project feedback and dissemination activities including co-facilitating and presenting at the interactive dissemination workshop / consensus meeting, publication authorship as peer researcher and presenting at conferences to showcase the project findings.

ETHICS AND DISSEMINATION

Ethics

The study was reviewed and received favourable opinion from the South Birmingham Research Ethics Committee (Ref. 19/WM/0133) on 12 June 2019; subsequent amendments have been approved. The current, approved protocol is version 4.0 dated 03 February 2021.

Safety

The trial intervention is conceptually similar to usual clinical practice (i.e., CYP referred to CAMHS may be sent questionnaires about their difficulties), therefore the risks of the trial are considered comparable. The DAWBA is widely used in research for data collection therefore, although utilised as an intervention in the STADIA trial, the risks may be regarded as similar to those of an observational/questionnaire study. Data to inform safety oversight will therefore be collected during routine follow-up, from existing outcome measures. There is no separate adverse event or serious adverse event reporting.

The number of participants meeting pre-defined safety outcomes will be reported on an ongoing basis to the Trial Management Group (TMG) and TSC. Data will be presented by arms to the Data Monitoring Committee (DMC).

Trial oversight

Nottinghamshire Healthcare NHS Foundation Trust will undertake role of Sponsor as defined by the UK Policy Framework for Health and Social Care Research.(44) Delegated responsibilities will be assigned to the Chief Investigator, participating NHS Trusts and the trial coordinating centre, Nottingham Clinical Trials Unit (NCTU).

The full co-applicant team and NCTU staff responsible for the day-to-day management of the trial will form the TMG, responsible for monitoring recruitment and retention rates and implementing strategies to ensure targets are met. Independent Trial Steering and Data Monitoring Committees will operate in accordance with trial-specific Charters.

Dissemination

Results of this trial will be reported to the funder and published in full in the HTA Journal series and also submitted for publication in a peer reviewed journal.

For peer review only - http://bmjopen.bmj.com/site/about/guidelines.xhtml

Data Sharing

Anonymised trial data may be shared with researchers external to the trial research team in accordance with the NCTU's data sharing procedure.

Figures

Figure 1: Participant flow

Authors' contributions

FD, LW, AB, BD, CE, JG, MJ, AL, TM, AM, SR, KSp, LT, EB, JL, KN, CP, KSt and KSa made substantial contributions to conception and design or acquisition of data; took part in drafting the article or revising it critically for important intellectual content; agreed to submit to the current journal; gave final approval of the version to be published; and agree to be accountable for all aspects of the work. KSa is guarantor for the paper. FD and LW contributed equally to this paper.

Funding statement

This study was funded as a result of a commissioned call by the National Institute for Health Research (NIHR) Health Technology Assessment programme (Grant Reference Number 16/96/09). The views expressed are those of the authors and not necessarily those of the NIHR or the Department of Health and Social Care.

The funder will have no role in the collection, management, analysis, and interpretation of data; writing of the report; and the decision to submit the report for publication.

Competing interests

The authors declare no competing interests.

Acknowledgements

We would like to acknowledge and thank all the parent/carers and young people participating in the trial and the research sites involved in recruiting participants and data collection. The authors would also like to thank the wider STADIA team for their input, including the PPI Advisory Panels, members of the independent Trial Steering and Data Monitoring Committees, and the Nottingham Clinical Trials Unit, who are the trial coordinating centre.

Finally, thanks to the trial sponsor, Nottinghamshire Healthcare NHS Foundation Trust (researchsponsor@nottshc.nhs.uk).

For peer review only - http://bmjopen.bmj.com/site/about/guidelines.xhtml

REFERENCES

1. Sadler K, Vizard T, Ford T, Marcheselli F, Pearce N, Mandalia D, et al. Mental Health of Children and Young People in England, 2017: NHS Digital; 2018 [Available from: https://digital.nhs.uk/data-and-information/publications/statistical/mental-health-of-children-and-young-people-in-england/2017/2017.

2. Goodyer IM RS, Barrett B, Byford S, Dubicka B, Hill J, Holland F, Kelvin R, Midgley N, Roberts C, Senior R, Target M, Widmer B, Wilkinson P, Fonagy P. Cognitive behavioural therapy and short-term psychoanalytic psychotherapy versus brief psychosocial intervention in adolescents with unipolar major depression (IMPACT): a multicentre, pragmatic, observer-blind, randomised controlled trial. Health Technology Assessment. 2017;21(12).

3. Simmons M, Wilkinson P, Dubicka B. Measurement Issues: Depression measures in children and adolescents. Child and Adolescent Mental Health. 2015;20(4):230-41.

4. Colman IW, MEJ. Croudace, TJ. Jones, PB. Forty-Year Psychiatric Outcomes Following Assessment for Internalizing Disorder in Adolescence. American Journal of Psychiatry. 2007;164(1):126-33.

5. Orchard F, Pass L, Marshall T, Reynolds S. Clinical characteristics of adolescents referred for treatment of depressive disorders. Child and Adolescent Mental Health. 2017;22(2):61-8.

6. Frith E. Access and waiting times in children and young people's mental health services.: Education Policy Institute; 2017 [Available from: https://epi.org.uk/wp-content/uploads/2018/01/EPI_Access-and-waiting-times_.pdf.

7. Michelson D, Rock S, Holliday S, Murphy E, Myers G, Tilki S, et al. Improving psychiatric diagnosis in multidisciplinary child and adolescent mental health services. The Psychiatrist. 2011;35:454-9.

8. O'Connor C, Downs J, McNicholas F, Cross L, Shetty H. Documenting diagnosis in child and adolescent mental healthcare: A content analysis of diagnostic statements in a psychiatric case register. Children and Youth Services Review. 2020;113:104948.

9. Allsopp K, Kinderman P. The use of diagnoses in mental health service eligibility and exclusion criteria. Journal of mental health (Abingdon, England). 2021;30(1):97-103.

10. Martin A-M, Fishman R, Baxter L, Ford T. Practitioners' attitudes towards the use of standardized diagnostic assessment in routine practice: A qualitative study in two Child and Adolescent Mental Health Services. Clinical Child Psychology and Psychiatry. 2011;16(3):407-20.

11. Craddock N, Antebi D, Attenburrow M-J, Bailey A, Carson A, Cowen P, et al. Wake-up call for British psychiatry. British Journal of Psychiatry. 2008;193(1):6-9.

12. National Institute for Health and Clinical Excellence. Depression in Children and Young People: Quality Standard London: National Institute for Health and Clinical Excellence; 2013 [Available from: https://www.nice.org.uk/guidance/cg28.

13. Fitzpatrick C AN-N, Kehoe A, Devlin N, Glackin S, Power L, et al. Do we miss depressive disorders and suicidal behaviours in clinical practice. Clinical Child Psychology and Psychiatry. 2012;17:449-58.

14. National Institute for Health and Clinical Excellence. Depression in Children and Young People: Identification and Management in Primary, Community and Secondary Care – Update London: National Institute for Health and Clinical Excellence,; 2015 [Available from: https://www.nice.org.uk/guidance/cg28.

15. Last A, Henley, W., Norman, S., Goodman, R., Ford, T. Innovations in Practice: Feasibility of the development and well-being assessment as an adjunct to clinical assessment in child and adolescent mental health services. . Child and Adolescent Mental Health. 2014;19:142-6.

16. Reeves K, Charter E, Ford T. Measurement Issues: Is standardised diagnostic assessment feasible as an adjunct to clinical practice? A systematic review. Child and Adolescent Mental Health. 2016;21(1):51-63.

For peer review only - http://bmjopen.bmj.com/site/about/guidelines.xhtml

17. Goodman R, Ford T, Richards H, Gatward R, Meltzer H. The Development and Well-Being Assessment: Description and Initial Validation of an Integrated Assessment of Child and Adolescent Psychopathology. Journal of Child Psychology and Psychiatry. 2000;41(5):645-55.
18. Hall CL, Davies EB, Andrén P, Murphy T, Bennett S, Brown BJ, et al. Investigating a therapist-guided, parent-assisted remote digital behavioural intervention for tics in children and adolescents—'Online Remote Behavioural Intervention for Tics' (ORBIT) trial: protocol of an internal pilot study and single-blind randomised controlled trial. BMJ Open. 2019;9(1):e027583.
19. Pickles A, Le Couteur A, Leadbitter K, Salomone E, Cole-Fletcher R, Tobin H, et al. Parent-mediated social communication therapy for young children with autism (PACT): long-term follow-up of a randomised controlled trial. The Lancet. 2016;388(10059):2501-9.
20. Ford T, Last A, Henley W, Norman S, Guglani S, Kelesidi K, et al. Can standardized diagnostic assessment be a useful adjunct to clinical assessment in child mental health services? A randomized controlled trial of disclosure of the Development and Well-Being Assessment to practitioners. Social Psychiatry and Psychiatric Epidemiology. 2013;48(4):583-93.
21. Aebi M, Kuhn C, Metzke CW, Stringaris A, Goodman R, Steinhausen H-C. The use of the development and well-being assessment (DAWBA) in clinical practice: a randomized trial. European child & adolescent psychiatry. 2012;21(10):559-67.
22. Meltzer H, Gatward R, Goodman R, Ford T. The mental health of children and adolescents in Great Britain London: Office for National Statistics; 2000 [Available from: http://webarchive.nationalarchives.gov.uk/20160105160709/http://www.ons.gov.uk/ons/rel/psychiatric-morbidity/the-mental-health-of-children-and-adolescents-in-great-britain/1999-survey/mental-health-of-children-and-adolescents-in-great-britain.pdf.
23. Green H, McGinnity A, Meltzer H, Ford T, Goodman R. Mental health of children and young people in Great Britain, 2004 Basingstoke, Hamshire: Palgrave Macmillan; 2005 [Available from: https://webarchive.nationalarchives.gov.uk/20180307185414/http://digital.nhs.uk/catalogue/PUB06116.
24. Youth in Mind. Information for researchers and clinicians about the Development and Well-Being Assessment: Youth in Mind; 2012 [Available from: http://www.dawba.info/.
25. Angold A, Costello EJ, Messer SC, Pickles A. Development of a short questionnaire for use in epidemiological studies of depression in children and adolescents. International Journal of Methods in Psychiatric Research. 1995;5(4):237-49.
26. Wood A, Kroll L, Moore A, Harrington R. Properties of the mood and feelings questionnaire in adolescent psychiatric outpatients: a research note. 1995;36:327-34.
27. Daviss WB, Birmaher B, Melhem NA, Axelson DA, Michaels SM, Brent DA. Criterion validity of the Mood and Feelings Questionnaire for depressive episodes in clinic and non-clinic subjects. 2006;47:927-34.
28. Chorpita BF, Yim L, Moffitt C, Umemoto LA, Francis SE. Assessment of symptoms of DSM-IV anxiety and depression in children: a revised child anxiety and depression scale. Behaviour Research and Therapy. 2000;38(8):835-55.
29. Chorpita BF, Moffitt CE, Gray J. Psychometric properties of the Revised Child Anxiety and Depression Scale in a clinical sample. Behaviour Research and Therapy. 2005;43(3):309-22.
30. Goodman R. The Extended Version of the Strengths and Difficulties Questionnaire as a Guide to Child Psychiatric Caseness and Consequent Burden. Journal of Child Psychology and Psychiatry. 1999;40(5):791-9.
31. Yao S, Zhang C, Zhu X, Jing X, McWhinnie CM, Abela JRZ. Measuring Adolescent Psychopathology: Psychometric Properties of the Self-Report Strengths and Difficulties Questionnaire in a Sample of Chinese Adolescents. Journal of Adolescent Health. 2009;45(1):55-62.
32. Goodman R. Psychometric Properties of the Strengths and Difficulties Questionnaire. Journal of the American Academy of Child & Adolescent Psychiatry. 2001;40(11):1337-45.
33. Muris P, Meesters C, van den Berg F. The Strengths and Difficulties Questionnaire (SDQ). European child & adolescent psychiatry. 2003;12(1):1-8.

For peer review only

For peer review only - http://bmjopen.bmj.com/site/about/guidelines.xhtml

34. Lundh LG, Wangby-Lundh, M., & Bjarehed, J. . Self reported emotional and behavioral problems in Swedish 14 to 15-year-old adolescents: A study with the self-report version of the Strengths and Difficulties Questionnaire. Scandinavian Journal of Psychology. 2008;49:523-32.
35. Kroenke K, Spitzer RL, Williams JB. The PHQ-9: validity of a brief depression severity measure. Journal of general internal medicine. 2001;16(9):606-13.
36. Spitzer RL, Kroenke K, Williams JW, Löwe B. A brief measure for assessing generalized anxiety disorder: The gad-7. Archives of Internal Medicine. 2006;166(10):1092-7.
37. Stevens K. Developing a descriptive system for a new preference-based measure of health-related quality of life for children. Quality of Life Research. 2009;18(8):1105-13.
38. Wille N, Badia X, Bonsel G, Burström K, Cavrini G, Devlin N, et al. Development of the EQ-5D-Y: a child-friendly version of the EQ-5D. Quality of life research : an international journal of quality of life aspects of treatment, care and rehabilitation. 2010;19(6):875-86.
39. Herdman M, Gudex C, Lloyd A, Janssen M, Kind P, Parkin D, et al. Development and preliminary testing of the new five-level version of EQ-5D (EQ-5D-5L). Quality of Life Research. 2011;20(10):1727-36.
40. Hall CL, James M, Brown S, Martin JL, Brown N, Selby K, et al. Protocol investigating the clinical utility of an objective measure of attention, impulsivity and activity (QbTest) for optimising medication management in children and young people with ADHD 'QbTest Utility for Optimising Treatment in ADHD' (QUOTA): a feasibility randomised controlled trial. BMJ Open. 2018;8(2):e021104.
41. Hoch JS, Briggs AH, Willan AR. Something old, something new, something borrowed, something blue: a framework for the marriage of health econometrics and cost-effectiveness analysis. Health Economics. 2002;11(5):415-30.
42. Gale NK, Heath G, Cameron E, Rashid S, Redwood S. Using the framework method for the analysis of qualitative data in multi-disciplinary health research. BMC Medical Research Methodology. 2013;13(1):117.
43. Furniss D, Lang AR, Ewart C. Enhancing Patient Ergonomics with Patient and Public Involvement in Research Projects. . In: Holden RJ, Valdez RS, editors. The Patient Factor: A Handbook on Patient Ergonomics: CRC Press; 2001.
44. Health Research Authority. UK Policy Framework for Health and Social Care Research: Health Research Authority; 2017 [Available from: https://www.hra.nhs.uk/planning-and-improving-research/policies-standards-legislation/uk-policy-framework-health-social-care-research/

For peer review only - http://bmjopen.bmj.com/site/about/guidelines.xhtml

Figure 1: Participant flow

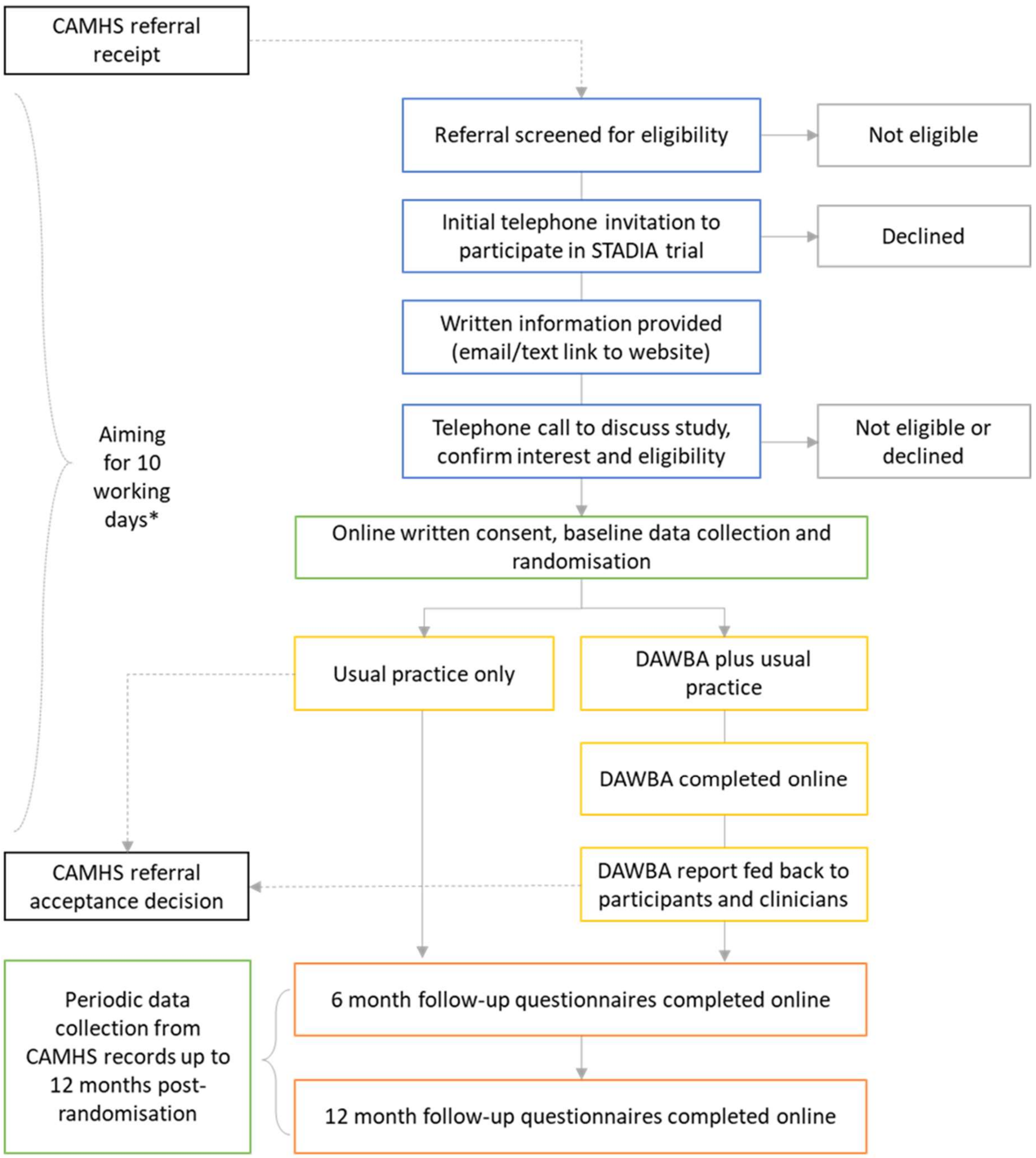

** For sites where the waiting time for the CAMHS acceptance decision usually exceeds 10 working days from referral receipt, recruitment activities may start and/or continue beyond 10 working days from referral receipt, providing the intervention period can be completed prior to the CAMHS referral decision.*

For peer review only

STAndardised DIagnostic Assessment for children and adolescents with emotional difficulties (STADIA): a multi-centre randomised controlled trial

SCREENING

**Site Number:**	
**Screening Number:**	
**Sponsor:**	**Nottinghamshire Healthcare NHS Foundation Trust**
**CRF Version:**	**Final v1.1 – 30 April 2019**

peer review only

Site Number: ☐☐

Screening Number: ☐☐☐

STADIA

REFERRAL SCREENING	
Complete for all referrals screened for eligibility:	
NHS Number<br>*Local use only*	______________________
Trust Number<br>*Local use only*	______________________
Date of referral receipt (dd-mmm-yyyy)	☐☐ - ☐☐☐ - ☐☐☐☐
Date of screening (dd-mmm-yyyy)	☐☐ - ☐☐☐ - ☐☐☐☐
Young person's sex	Male ☐<br>Female ☐
Young person's age<br>*If <5 or >17 do not proceed*	☐☐
Has the young person been previously enrolled and randomised in the STADIA trial?<br>*If yes, do not proceed*	Yes ☐<br>No ☐
Does the referral mention any of the following Covid-19 related words/phrases?<br>*Tick all that apply.*<br>Covid-19 / Covid<br>Coronavirus<br>Lockdown<br>School closure / exams cancelled	 <br>☐<br>☐<br>☐<br>☐
Does the referral mention emotional difficulties*?<br>*If no, do not proceed*	Yes ☐<br>No ☐
Is this an emergency or urgent referral (according to local CAMHS triage / SPA team risk assessment)?<br>*If yes, do not proceed*	Yes ☐<br>No ☐
Does the young person have severe learning disability (e.g., the referral mentions this or that they attend a special school for children with severe learning difficulties)?<br>*If 'yes' do not proceed*<br>*If not known, confirm during telephone eligibility check at enrolment*	Yes ☐<br>No ☐<br>Not known ☐

For peer review only

For peer review only - http://bmjopen.bmj.com/site/about/guidelines.xhtml

Site Number: | | |

Screening Number: | | | |

REFERRAL SCREENING	
If the young person is <16:<br>Does the referral information include contact details for a named parent/carer?<br>*If 'no' await parent/carer contact details before proceeding*	Yes ☐<br>No ☐<br>N/A ☐
If the young person is <16:<br>Is the named parent/carer a local authority representative designated to care for the child/young person?<br>*If 'yes' do not proceed*<br>*If not known, confirm during telephone eligibility check at enrolment*	Yes ☐<br>No ☐<br>Not known ☐<br>N/A ☐
If the young person is aged 16 or 17:<br>Whose contact details are given on the referral form?<br>*If young person contact details are provided, they should be contacted in the first instance*	Young person ☐<br>Parent/carer ☐<br>Both ☐<br>N/A ☐

EMOTIONAL DIFFICULTIES	
**Emotional difficulties may be indicated by the use of any of the following key words or phrases.*<br>*Tick all that apply. If 'other' record details and seek advice from the PI or NCTU before proceeding.*	
None	☐
Agitated / agitation	☐
Anger	☐
Anxiety / anxious / generalised anxiety	☐
Avoids things/people/places	☐
Can't leave the house	☐
Completing rituals / asking parents to carry out rituals	☐
Compulsions	☐
Depressed / depression / low / low mood / sad	☐
Difficulties sleeping	☐
Distress	☐
Fears and worries / fears relating to safety (germs, fire)	☐
Feeling low	☐
Feels flat / empty / blank	☐

For peer review only - http://bmjopen.bmj.com/site/about/guidelines.xhtml

Site Number: | | |

Screening Number: | | | |

EMOTIONAL DIFFICULTIES	
***Emotional difficulties may be indicated by the use of any of the following key words or phrases.** *Tick all that apply. If 'other' record details and seek advice from the PI or NCTU before proceeding.*	
Feels hopeless	☐
Feels worthless / stupid	☐
Flashbacks	☐
Hypervigilance	☐
Irritable	☐
Low motivation	☐
Low self-esteem / Hates self	☐
Mood swings / moody	☐
Negative thoughts	☐
Nightmares (if trauma also present)	☐
No (or loss of) energy	☐
No (or loss of) interest in things / gave up… / lack of wanting to do things	☐
Not going to school / unable to go to school	☐
Not sleeping / poor sleep	☐
Obsessions	☐
OCD	☐
Phobia	☐
Panic / panic attacks	☐
PTSD	☐
Self-harm / DSH / Cutting	☐
Suicidal	☐
Suicidal thoughts / thoughts of ending life / thinks about killing self	☐
Tearful	☐
Thoughts of death	☐

For peer review only

For peer review only - http://bmjopen.bmj.com/site/about/guidelines.xhtml

Site Number: |__|__|

Screening Number: |__|__|__|

EMOTIONAL DIFFICULTIES	
**Emotional difficulties may be indicated by the use of any of the following key words or phrases. Tick all that apply. If 'other' record details and seek advice from the PI or NCTU before proceeding.*	
Tiredness / fatigue	☐
Touching objects	☐
Trauma	☐
Weepy	☐
Withdrawal / withdrawn	☐
Worried / worrying (incl. worries/concerns about their appearance	☐
Other (please specify)	☐

FOR ALL REFERRALS SCREENED, ENTER SUMMARY DATA ON THE SCREENING & ENROLMENT LOG.

IF THE YOUNG PERSON APPEARS TO BE ELIGIBLE PROCEED TO THE INVITATION TELEPHONE CALL (CALL 1) AND ENTER DETAILS ON THE TRIAL DATABASE.

SIGN-OFF STATEMENT	
Completed by the researcher conducting the referral screening.	
To the best of my knowledge, I confirm that I have made every reasonable effort to ensure that ALL of the data in this Case Record Form is a true, accurate and complete report.	
Print Name	
Signature	
Date	|__|__| - |__|__|__| - |__|__|__|__|

For peer review only

For peer review only - http://bmjopen.bmj.com/site/about/guidelines.xhtml

ADD LOCAL HEADER

STandardised DIagnostic Assessment for children and young people with emotional difficulties (STADIA)

Informed Consent Form for the Parent/Carer

Final v2.0 13 August 2020

Name of Principal Investigator: [add local PI name]

IRAS Project ID: 255635

Participant Trial ID:
(To be completed after randomisation)

We are doing this research to find out how to make sure children and young people get the help they need when they are referred to CAMHS. We have invited you to take part in this research because a young person you care for has been referred to CAMHS. You can decide whether or not to take part in this research.
If you agree to take part in the STADIA Trial, please read and acknowledge each of the following statements.

A drop-down menu will be provided within the online electronic Informed Consent Form so that the person providing consent has the option to acknowledge/agree to each of the following statements.	
**1.**	I confirm that I have read and understand the Participant Information Sheet, Version <insert current PIS version number and date > for the above research. (*Only for the parent/carer of children/young people aged 11-15*) [My child and] I have had the opportunity to consider the information, ask questions and have had these answered satisfactorily.
**2.**	*Only for the parent/carer of children/young people aged 11-15*<br>I have spoken to my child about the research and they are aware of the study.
**3.**	I understand that mine and my child's participation is voluntary and that I am free to withdraw at any time, without giving any reason, and without my child's medical care or legal rights being affected. I understand that should I withdraw, then the information collected so far cannot be deleted and that this information may still be used in the research.
**4.**	I understand that relevant sections of my child's CAMHS records and data collected in the trial may be looked at by authorised individuals from the Nottingham Clinical Trials Unit (University of Nottingham), the Sponsor (Nottinghamshire Healthcare NHS Foundation Trust), NHS bodies, the trial research group and regulatory authorities where it is relevant to taking part in this study. I give permission for these individuals to have access to these records and for my consent form to be retained by the Nottingham Clinical Trials Unit.
**5.**	I give permission for the Nottingham Clinical Trials Unit, the Sponsor and the trial research group to collect, store, analyse and publish information obtained from mine and my child's participation in this trial. I understand that our personal details will be kept confidential.
**6.**	I understand that the Nottingham Clinical Trials Unit and the trial research group will be provided with mine and my child's personal details to send questionnaires by email and study-related correspondence during the trial. I give my permission for this information to be kept and for these individuals to contact me.
**7.**	I understand that if I fill out the DAWBA, I will receive a copy of the DAWBA report and a copy will also be provided to the CAMHS team and kept in my child's CAMHS records.
**8.**	I agree to my child's GP being informed of their participation in this trial.
**9.**	I understand that the anonymised information collected about me and my child may be used to support other research in the future and may be shared with other researchers.
**10.**	I agree to take part in the above trial.

For peer review only

For peer review only - http://bmjopen.bmj.com/site/about/guidelines.xhtml

Please also answer yes or no to the following options.

A drop-down menu will be provided within the online electronic Informed Consent Form so that the person providing consent has the option to answer yes or no to each of the following optional statements.			
**1.**	*Interviews about your experiences*<br>I agree to be contacted about the STADIA interview study. I understand that there is no obligation to take part and I will just be informed of what the study will involve.	Yes	No
**2.**	*Future studies*<br>I agree to be contacted about other research studies in the future. I understand that there is no obligation to take part and I will just be informed of what the future research would involve.	Yes	No
**3.**	*Results of the STADIA study*<br>I would like to receive a summary of the results at the end of the STADIA study.	Yes	No
**4.**	*Only for the parent/carer of children/young people aged 11-15*<br>*Questionnaires*<br>I agree to my child being invited to complete questionnaires about their mood and feelings for the research.	Yes	No
**5.**	I consent to [INSERT NHS TRUST NAME] passing identifiable data (my child's NHS number, name and date of birth) to the organisations that are responsible for health information including NHS Digital. This will be used to request data from the Children and Young People's Health Services Data Set and the Mental Health Services Data Set.	Yes	No

Type your name here:

________________________ _______________

Name of parent/carer Date [system generated]

Type the name of your child here:

________________________ _______________

Name of child/young person Date [system generated]

System use only:

________________________ _______________

Name of person taking consent Date [system generated]
(You must be on the delegation log)

NB. Signatures will not be collected as consent will be obtained online. Participants will be asked to complete the eICF and write their name before submitting the online form; the date will be system-generated. The name of the researcher who provided the study information and the date the eICF was generated will also be recorded within the online system.

The online electronic Informed Consent Form (eICF) will be retained within the trial database. Printable (PDF) copies will be generated and retained within the Investigator Site File and CAMHS records. A copy will be sent by email to the participant.

For peer review only - http://bmjopen.bmj.com/site/about/guidelines.xhtml

ADD LOCAL HEADER

STandardised DIagnostic Assessment for children and young people with emotional difficulties (STADIA)

Assent form for young people aged 11-15

Final v1.0 28-Mar-2019

Name of Principal Investigator: [add local PI name]

IRAS Project ID: 255635

Participant Trial ID: | | | | | |

(To be completed after randomisation)

We are doing this research to find out how to make sure people get the help they need when they are referred to CAMHS. We are asking you to help with this research but you can decide whether or not to take part.

If you agree to help with the STADIA Trial please answer the following questions.

A drop-down menu will be provided within the online electronic Assent Form so that the young person providing assent has the option to acknowledge/agree to each of the following statements.			
**1.**	Have you read the information about the research or has someone explained it to you?	Yes	No
**2.**	Do you understand what the research is about?	Yes	No
**3.**	Have you been able to ask all the questions you want?	Yes	No
**4.**	Do you understand that it's your choice whether or not to take part and it's OK to stop taking part at any time?	Yes	No
**5.**	Do you want to help with the research by completing some questionnaires about your mood and feelings?	Yes	No

Type your name here:

_________________________ _______________

Name of child/young person Date [system generated]

System use only:

_________________________ _______________

Name of person taking consent Date [system generated]

(You must be on the delegation log)

NB. Signatures will not be collected as consent will be obtained online. Participants will be asked to complete the eICF and write their name before submitting the online form; the date will be system-generated. The name of the researcher who provided the study information and the date the eICF was generated will also be recorded within the online system. The online electronic Informed Consent Form (eICF) will be retained within the trial database. Printable (PDF) copies will be generated and retained within the Investigator Site File and CAMHS records. A copy will be sent by email to the participant.

For peer review only - http://bmjopen.bmj.com/site/about/guidelines.xhtml

DAWBA Report

The DAWBA collects information about a range of common emotional and behavioural difficulties, and uses this information to produce a report to highlight the level of difficulties.

How to understand the ratings

These ratings compare your responses with the responses from large numbers of other parents and young people across the UK. Many parents and young people find this sort of comparison helpful, but it is just a guide and not the same as a face-to-face assessment with a specialist.

To make it easier to read, we have grouped the ratings into four categories. Each category is different. This shows how your [child's] *(delete as appropriate)* difficulties compare with other children / young people:

For Peer Review

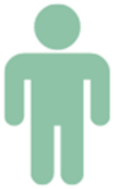

Close to average

In the general population most children/ young people (roughly 80 out of 100) are in the "close to average" category.

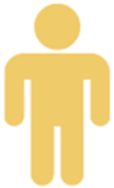

Slightly raised

If the ratings are in the "slightly raised" category this means the difficulties are slightly higher than average. Roughly 10 out of 100 children / young people are in this category.

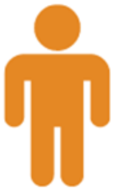

High

Around 5 in 100 children / young people score in the "high" category. This means that the difficulties are more severe than average.

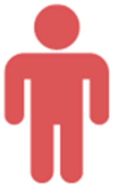

Very high

Around 5 in 100 children score in the "very high" category. This means that the difficulties appear to be more severe than we find in 95 out of every 100 children / young people.

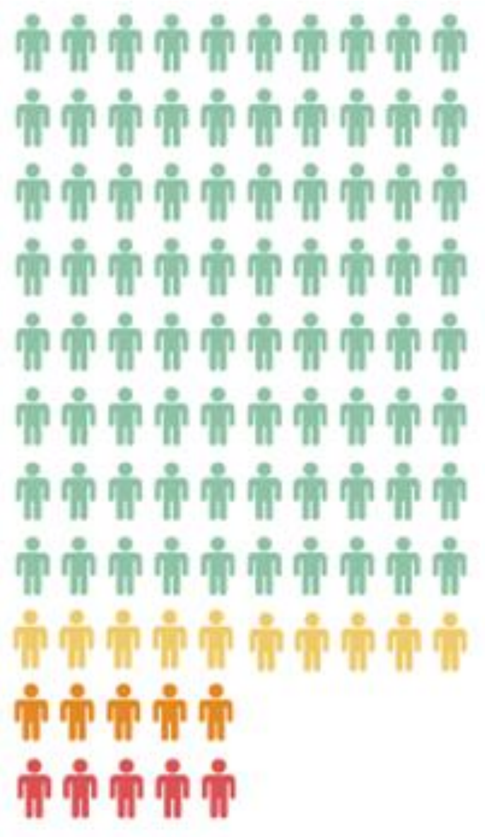

The rating is only a rough guide. As high ratings can be a "false alarm", please use your own judgement. Not all difficulties need treating. Some difficulties get better by themselves, particularly if they are mild or if they have only been there for a short time.

Most strengths and difficulties lie on a scale. There will be children / young people at each end of the scale but most children / young people will fall somewhere in between.

For peer review only - http://bmjopen.bmj.com/site/about/guidelines.xhtml

Your [child's] *(delete as appropriate)* **ratings:**

- **Close to average / Slightly raised / High / Very high** for worrying a lot about different things (general fears and worries)
- **Close to average / Slightly raised / High / Very high** for worries about separation from key "attachment figures" such as parents (separation anxiety)
- **Close to average / Slightly raised / High / Very high** for specific fears (specific phobia)
- **Close to average / Slightly raised / High / Very high** for social fears (social anxiety)
- **Close to average / Slightly raised / High / Very high** for panic attacks
- **Close to average / Slightly raised / High / Very high** for fears of crowds, public places, open spaces etc (agoraphobia)
- **Close to average / Slightly raised / High / Very high** for stress linked to particularly frightening events (post-traumatic stress)
- **Close to average / Slightly raised / High / Very high** for obsessions or compulsions
- **Close to average / Slightly raised / High / Very high** for depression or loss of interest
- **Close to average / Slightly raised / High / Very high** for disruptive and uncooperative behaviours (troublesome behaviour)
- **Close to average / Slightly raised / High / Very high** for antisocial or aggressive behaviours that can get people into serious trouble (troublesome behaviour)

For peer review only

For peer review only - http://bmjopen.bmj.com/site/about/guidelines.xhtml

Appendix 5. Eligible emotional disorder diagnoses

Anxiety disorder
Separation anxiety disorder
Specific phobia (any)
Social phobia or Social anxiety disorder
Agoraphobia
Panic disorder (DSM5 additionally has Panic Attack with a specifier)
Phobic anxiety disorder (unspecified)
Selective mutism
Generalized anxiety disorder
Obsessive-compulsive and related disorders
Body dysmorphic disorder
Acute stress reaction
Acute Stress Disorder
Post-traumatic stress disorder
Adjustment Disorder
Other anxiety disorder
Mixed anxiety and depressive disorder
Depression
Depressive episode (any / mild / moderate / severe)
Depressive disorder
Recurrent depressive disorder (any / mild / moderate / severe)
Major Depressive disorder
Persistent Depressive disorder
Other depressive episode
Persistent mood (affective) disorder (including cyclothymic disorder / dysthymic disorder)
Other / Unspecified mood (affective) disorder
Bipolar disorder
Bipolar affective disorder
Manic episode
Childhood emotional disorder unspecified (F93.9)

only

STAndardised DIagnostic Assessment for children and adolescents with emotional difficulties (STADIA): A multi-centre randomised controlled trial

OUTCOME DEFINITION & ADJUDICATION PLAN

Final 1.0 – 25 February 2020

For peer review only

EMOTIONAL DISORDER DIAGNOSES RECORDED IN THE 12 MONTHS POST-RANDOMISATION

CONSTITUTES A CLINICAL DIAGNOSIS	REFER FOR ADJUDICATION	DOES NOT CONSTITUTE A CLINICAL DIAGNOSIS
- The presence of an eligible diagnosis within the diagnosis tab of the clinical record.<br>- The presence of an eligible diagnosis in the clinical record preceded by the heading 'diagnosis'.<br>- The presence of an eligible diagnosis in the clinical record preceded by a heading such as 'current difficulties' or 'presenting problems', except where this has been documented in the write up of the first appointment or in reference to the information received at referral (as this may simply reflect a pre-existing or referrer-made diagnosis).<br>- A clear confirmatory statement including use of an eligible diagnosis, for example:<br>*Meets the diagnostic criteria for…*<br>*Presentation is explained by a diagnosis of…*	- The presence of similar diagnostic terms within the diagnosis tab of the clinical record.<br>- The presence of an eligible diagnosis preceded by a heading such as 'current difficulties' or 'presenting problems', documented in the write up of the first appointment or in reference to the information received at referral.<br>- The presence of similar diagnostic terms preceded by a heading such as 'diagnosis', 'current difficulties' or 'presenting problems'.<br>- Reference to an eligible diagnosis or similar diagnostic terms, but where the context does not provide a clear confirmatory statement, for example:<br>*?...*<br>*Possible…*<br>*Assessed for…*<br>*…-type symptoms / behaviour*<br>*…-like symptoms / behaviour*<br>*Symptoms of…*<br>*History of…*	- No reference to an eligible diagnosis or similar diagnostic terms.<br>- A clear statement about the absence of an eligible diagnosis or similar diagnostic terms, for example:<br>*No evidence of…*<br>*…not meeting criteria for disorder*
Data collection and entry: instructions for researchers		
Use the checklist of eligible emotional disorder diagnoses.	*Document these as other emotional disorders.*	*Check 'none of the above' in the checklist of emotional disorder diagnoses and answer 'no' to 'other emotional disorder diagnoses'.*

Note: For definition of underlined terms see the Glossary below.

For peer review only - http://bmjopen.bmj.com/site/about/guidelines.xhtml

NO EMOTIONAL DISORDER

If there are no emotional disorder diagnoses documented in the CAMHS notes in 12 months post-randomisation, researchers will select one of the following options:

1. A clinician has documented the absence of emotional disorder.
2. Uncertainty about the presence of an emotional disorder is documented in the notes (for example, reflecting ongoing assessment / investigation).
3. There is no diagnostic information relating to emotional disorders documented in the CAMHS record.

A clinician has documented the absence of emotional disorder.	Uncertainty about the presence of an emotional disorder is documented in the notes (for example, reflecting ongoing assessment / investigation).	There is no diagnostic information relating to emotional disorders documented in the CAMHS record.
- Nothing in the clinical record is assessed to constitute a documented clinical diagnosis, AND<br>- There is a clear statement about the absence of one or more of the eligible diagnoses or similar diagnostic terms, for example:<br><br>*No evidence of…*<br>*…not meeting criteria for disorder*	- Nothing in the clinical record is assessed to constitute a documented clinical diagnosis, AND<br>- Reference to an eligible diagnosis or similar diagnostic terms, but where the context does not provide a clear confirmatory statement, for example:<br><br>*?...*<br>*Possible…*<br>*Assessed for…*<br>*…-type symptoms / behaviour*<br>*…-like symptoms / behaviour*<br>*Symptoms of…*<br>*History of…*	- Nothing in the clinical record is assessed to constitute a documented clinical diagnosis, AND<br>- There is no reference to an eligible diagnosis or similar diagnostic terms.*<br>- If emotional difficulties are identified they are described only by reference to the presenting symptoms with no attempt made to link these to an eligible diagnosis, for example:<br><br>*Presenting issue - Mood swings*<br>*Describing examples of ruminating thoughts.*<br><br>* Note that this includes children/young people who have not been seen by CAMHS in the 12-months post-randomisation.
Document these as absence of emotional disorder.	**REFER FOR ADJUDICATION**	**MAY REQUIRE ADJUDICATION**

EMOTIONAL DISORDER DIAGNOSIS ADJUDICATION OUTCOME	
The Adjudication Committee will first consider whether the record:<br>1) Constitutes a clinical diagnosis<br>2) Does not constitute a clinical diagnosis	If (1) then the Adjudication Committee will determine which of the eligible emotional disorder diagnoses apply.<br><br>If (2) then the Adjudication Committee will determine whether the record constitutes:<br>a) Absence of emotional disorder<br>b) Uncertainty about the presence of emotional disorder<br>c) No diagnostic information

For peer review only - http://bmjopen.bmj.com/site/about/guidelines.xhtml

TREATMENTS / INTERVENTIONS GIVEN

CONSTITUTES A TREATMENT / INTERVENTION	REFER FOR ADJUDICATION
- The presence of an eligible treatment / intervention documented within the clinical record.	- Documented intervention by CAMHS where the description does not include an eligible treatment / intervention.
Data collection and entry: instructions for researchers	
Use the checklist of eligible treatments / interventions.	*Document these as other treatments / interventions.*

TREATMENTS / INTERVENTIONS ADJUDICATION OUTCOME	
The Adjudication Committee will first consider whether the record:<br>1) Constitutes a treatment / intervention<br>2) Does not constitute a treatment / intervention	If (1) then the Adjudication Committee will determine whether the record should be categorised:<br>a) As an existing treatment / intervention<br>b) As an 'other' treatment / intervention

GLOSSARY	
Eligible diagnosis	One of the pre-specified diagnoses listed on the data collection form. These should be considered present only when the exact phrase and/or corresponding ICD/DSM code is documented.
Similar diagnostic terms	Words or phrases which are similar to the eligible diagnoses, but without use of the exact wording or corresponding ICD/DSM code (e.g., separation anxiety WITHOUT use of the term disorder) or where the exact words are used alongside additional phrases (e.g., OCD-type behaviour or OCD-like symptoms).
Eligible treatment / intervention	One of the pre-specified treatments / interventions listed on the data collection form.
Adjudication Committee	The Adjudication Committee will comprise the clinician members of the Trial Management Group. A minimum of two clinicians will review terms referred for adjudication, with a third consulted if a consensus is not reached.<br>The Adjudication Committee will be blinded to treatment allocation for the purposes of adjudication.

For peer review only - http://bmjopen.bmj.com/site/about/guidelines.xhtml

Appendix 7. Summary of assessments

**Time-point**	**Maximum 10 working days from referral receipt[1]**					**6 months post-randomisation**	**12 months post-randomisation**
**Activity**	**Screening and invitation**	**Eligibility and enrolment**	**Consent and baseline**	**Randomisation**	**Intervention**<br><br>DAWBA in addition to usual practice<br><br>Or<br><br>Usual practice only	**Follow-Up**	
Initial eligibility screen of referral information	X						
Telephone invitation to participate	X						
Verbal agreement to participate		X					
Confirm eligibility		X					
Obtain enrolment data		X					
Participant enrolment		X					
Written informed consent/assent (online)			X				
Baseline demographics (parent/carer and CYP aged 16 & 17)			X				
Mood and Feelings Questionnaire (MFQ)			X			X	X
Revised Child's Anxiety Depression Scale (RCADS)			X			X	X
Strengths and Difficulties Questionnaire (SDQ)[2]			X			X	X
Child Revised Impact of Events Scale (CRIES-8)(42)[3]			X			X	X
CYP self-report self-harm measure			X			X	X
Patient Health Questionnaire (PHQ-9) - parent/carer only			X			X	X
Generalised Anxiety Disorder Assessment (GAD-7) - parent/carer only			X			X	X
Child Health Utility 9D (CHU9D)			X			X	X
EuroQol-5D youth (EQ-5D-Y)			X			X	X
EuroQol-5D five level (EQ-5D-5L)			X			X	X
Resource Use Questionnaire - parent/carer and CYP aged 16 & 17			X			X	X
Data collection from records[4]			X			X	X

[1] For sites where the waiting time for the CAMHS acceptance decision usually exceeds 10 working days from referral receipt, recruitment activities may start and/or continue beyond 10 working days from referral receipt, providing the intervention period can be completed prior to the CAMHS referral decision.
[2] For participants in the intervention arm, the baseline SDQ will be collected as part of the DAWBA, completed post-randomisation.
[3] Additional data collection undertaken to explore post-traumatic stress disorder symptoms in CYP during the Covid-19 pandemic
[4] Data collection from records will be completed periodically throughout the 12 month follow-up period.

For peer review only

Reporting checklist for protocol of a clinical trial.

Based on the SPIRIT guidelines.

Instructions to authors

Complete this checklist by entering the page numbers from your manuscript where readers will find each of the items listed below.

Your article may not currently address all the items on the checklist. Please modify your text to include the missing information. If you are certain that an item does not apply, please write "n/a" and provide a short explanation.

Upload your completed checklist as an extra file when you submit to a journal.

In your methods section, say that you used the SPIRITreporting guidelines, and cite them as:

Chan A-W, Tetzlaff JM, Gøtzsche PC, Altman DG, Mann H, Berlin J, Dickersin K, Hróbjartsson A, Schulz KF, Parulekar WR, Krleža-Jerić K, Laupacis A, Moher D. SPIRIT 2013 Explanation and Elaboration: Guidance for protocols of clinical trials. BMJ. 2013;346:e7586

		Reporting Item	Page Number (line)
**Administrative information**			

For peer review only

Title	#1	Descriptive title identifying the study design, population, interventions, and, if applicable, trial acronym	1(1-2)
Trial registration	#2a	Trial identifier and registry name. If not yet registered, name of intended registry	2 (68)
Trial registration: data set	#2b	All items from the World Health Organization Trial Registration Data Set	Throughout
Protocol version	#3	Date and version identifier	19 (511)
Funding	#4	Sources and types of financial, material, and other support	20 (556-559)
Roles and responsibilities: contributorship	#5a	Names, affiliations, and roles of protocol contributors	1 (5-9)
Roles and responsibilities: sponsor contact information	#5b	Name and contact information for the trial sponsor	20 (574-575)
Roles and responsibilities: sponsor and funder	#5c	Role of study sponsor and funders, if any, in study design; collection, management, analysis, and interpretation of data; writing of the report; and the decision to submit the report for publication, including whether they will have ultimate authority over any of these activities	20 (556-562)

For peer review only

Roles and responsibilities: committees	#5d	Composition, roles, and responsibilities of the coordinating centre, steering committee, endpoint adjudication committee, data management team, and other individuals or groups overseeing the trial, if applicable (see Item 21a for data monitoring committee)	19 (532-535)
**Introduction**			
Background and rationale	#6a	Description of research question and justification for undertaking the trial, including summary of relevant studies (published and unpublished) examining benefits and harms for each intervention	3-4 (91-148)
Background and rationale: choice of comparators	#6b	Explanation for choice of comparators	3-4, 7-8 (129-148)
Objectives	#7	Specific objectives or hypotheses	4 (150-176)
Trial design	#8	Description of trial design including type of trial (eg, parallel group, crossover, factorial, single group), allocation ratio, and framework (eg, superiority, equivalence, non-inferiority, exploratory)	4 (182-183)
**Methods: Participants,**			

For peer review only

**interventions, and outcomes**			
Study setting	#9	Description of study settings (eg, community clinic, academic hospital) and list of countries where data will be collected. Reference to where list of study sites can be obtained	5 (190-193)
Eligibility criteria	#10	Inclusion and exclusion criteria for participants. If applicable, eligibility criteria for study centres and individuals who will perform the interventions (eg, surgeons, psychotherapists)	5-6 (see table 1)
Interventions: description	#11a	Interventions for each group with sufficient detail to allow replication, including how and when they will be administered	7-8 (269-303)
Interventions: modifications	#11b	Criteria for discontinuing or modifying allocated interventions for a given trial participant (eg, drug dose change in response to harms, participant request, or improving / worsening disease)	n/a
Interventions: adherance	#11c	Strategies to improve adherence to intervention protocols, and any procedures for monitoring adherence (eg, drug tablet return; laboratory tests)	7-8 (269-303)
Interventions: concomitant care	#11d	Relevant concomitant care and interventions that are permitted or prohibited during the trial	7-8 (269-303)

For peer review only

Outcomes	#12	Primary, secondary, and other outcomes, including the specific measurement variable (eg, systolic blood pressure), analysis metric (eg, change from baseline, final value, time to event), method of aggregation (eg, median, proportion), and time point for each outcome. Explanation of the clinical relevance of chosen efficacy and harm outcomes is strongly recommended	8-15 (316-337)
Participant timeline	#13	Time schedule of enrolment, interventions (including any run-ins and washouts), assessments, and visits for participants. A schematic diagram is highly recommended (see Figure 1)	5-7 (197-239) and see figure 1
Sample size	#14	Estimated number of participants needed to achieve study objectives and how it was determined, including clinical and statistical assumptions supporting any sample size calculations	8 (306-312)
Recruitment	#15	Strategies for achieving adequate participant enrolment to reach target sample size	5 (197-212)
**Methods: Assignment of interventions (for controlled trials)**			

For peer review only

Allocation: sequence generation	#16a	Method of generating the allocation sequence (eg, computer-generated random numbers), and list of any factors for stratification. To reduce predictability of a random sequence, details of any planned restriction (eg, blocking) should be provided in a separate document that is unavailable to those who enrol participants or assign interventions	7 (242-246)
Allocation concealment mechanism	#16b	Mechanism of implementing the allocation sequence (eg, central telephone; sequentially numbered, opaque, sealed envelopes), describing any steps to conceal the sequence until interventions are assigned	7 (248-252)
Allocation: implementation	#16c	Who will generate the allocation sequence, who will enrol participants, and who will assign participants to interventions	7 (244-252)
Blinding (masking)	#17a	Who will be blinded after assignment to interventions (eg, trial participants, care providers, outcome assessors, data analysts), and how	7 (254-260)
Blinding (masking): emergency unblinding	#17b	If blinded, circumstances under which unblinding is permissible, and procedure for revealing a participant's allocated intervention during the trial	7 (254-256)
**Methods: Data collection,**			

For peer review only

**management, and analysis**			
Data collection plan	#18a	Plans for assessment and collection of outcome, baseline, and other trial data, including any related processes to promote data quality (eg, duplicate measurements, training of assessors) and a description of study instruments (eg, questionnaires, laboratory tests) along with their reliability and validity, if known. Reference to where data collection forms can be found, if not in the protocol	16 (368-375)
Data collection plan: retention	#18b	Plans to promote participant retention and complete follow-up, including list of any outcome data to be collected for participants who discontinue or deviate from intervention protocols	16 (369-375)
Data management	#19	Plans for data entry, coding, security, and storage, including any related processes to promote data quality (eg, double data entry; range checks for data values). Reference to where details of data management procedures can be found, if not in the protocol	16 (379-387)
Statistics: outcomes	#20a	Statistical methods for analysing primary and secondary outcomes. Reference to where other	17 (389-408)

For peer review only

		details of the statistical analysis plan can be found, if not in the protocol	
Statistics: additional analyses	#20b	Methods for any additional analyses (eg, subgroup and adjusted analyses)	17 (389-408)
Statistics: analysis population and missing data	#20c	Definition of analysis population relating to protocol non-adherence (eg, as randomised analysis), and any statistical methods to handle missing data (eg, multiple imputation)	17 (390-392)
**Methods: Monitoring**			
Data monitoring: formal committee	#21a	Composition of data monitoring committee (DMC); summary of its role and reporting structure; statement of whether it is independent from the sponsor and competing interests; and reference to where further details about its charter can be found, if not in the protocol. Alternatively, an explanation of why a DMC is not needed	19 (534-535)
Data monitoring: interim analysis	#21b	Description of any interim analyses and stopping guidelines, including who will have access to these interim results and make the final decision to terminate the trial	19 (527-535)
Harms	#22	Plans for collecting, assessing, reporting, and managing solicited and spontaneously reported adverse events and other unintended effects of trial interventions or trial conduct	19 (514-524)

For peer review only

Auditing	#23	Frequency and procedures for auditing trial conduct, if any, and whether the process will be independent from investigators and the sponsor	19 (532-535)
**Ethics and dissemination**			
Research ethics approval	#24	Plans for seeking research ethics committee / institutional review board (REC / IRB) approval	19 (509-511)
Protocol amendments	#25	Plans for communicating important protocol modifications (eg, changes to eligibility criteria, outcomes, analyses) to relevant parties (eg, investigators, REC / IRBs, trial participants, trial registries, journals, regulators)	19 (509-511)
Consent or assent	#26a	Who will obtain informed consent or assent from potential trial participants or authorised surrogates, and how (see Item 32)	6 (see table 2)
Consent or assent: ancillary studies	#26b	Additional consent provisions for collection and use of participant data and biological specimens in ancillary studies, if applicable	6 (table 2)
Confidentiality	#27	How personal information about potential and enrolled participants will be collected, shared, and maintained in order to protect confidentiality before, during, and after the trial	16 (384-387)

For peer review only

Declaration of interests	#28	Financial and other competing interests for principal investigators for the overall trial and each study site	20 (565)
Data access	#29	Statement of who will have access to the final trial dataset, and disclosure of contractual agreements that limit such access for investigators	16 (384-387)
Ancillary and post trial care	#30	Provisions, if any, for ancillary and post-trial care, and for compensation to those who suffer harm from trial participation	n/a
Dissemination policy: trial results	#31a	Plans for investigators and sponsor to communicate trial results to participants, healthcare professionals, the public, and other relevant groups (eg, via publication, reporting in results databases, or other data sharing arrangements), including any publication restrictions	19 (538-539)
Dissemination policy: authorship	#31b	Authorship eligibility guidelines and any intended use of professional writers	20 (549-553)
Dissemination policy: reproducible research	#31c	Plans, if any, for granting public access to the full protocol, participant-level dataset, and statistical code	20 (542-543)
**Appendices**			

For peer review only

Informed consent materials	#32	Model consent form and other related documentation given to participants and authorised surrogates	Supplementary materials 6 & 7
Biological specimens	#33	Plans for collection, laboratory evaluation, and storage of biological specimens for genetic or molecular analysis in the current trial and for future use in ancillary studies, if applicable	n/a

None The SPIRIT Explanation and Elaboration paper is distributed under the terms of the Creative Commons Attribution License CC-BY-NC. This checklist can be completed online using https://www.goodreports.org/, a tool made by the EQUATOR Network in collaboration with Penelope.ai

For peer review only
